# Blockage of the Epithelial-to-Mesenchymal Transition Is Required for Embryonic Stem Cell Derivation

**DOI:** 10.1016/j.stemcr.2017.08.006

**Published:** 2017-09-14

**Authors:** Mehdi Totonchi, Seyedeh-Nafiseh Hassani, Ali Sharifi-Zarchi, Natalia Tapia, Kenjiro Adachi, Julia Arand, Boris Greber, Davood Sabour, Marcos J. Araúzo-Bravo, Jörn Walter, Mohammad Pakzad, Hamid Gourabi, Hans R. Schöler, Hossein Baharvand

**Affiliations:** 1 Department of Stem Cells and Developmental Biology, Cell Science Research Center, Royan Institute for Stem Cell Biology and Technology, ACECR, Tehran, Iran; 2 Department of Genetics, Reproductive Biomedicine Research Center, Royan Institute for Reproductive Biomedicine, ACECR, Tehran, Iran; 3 Department of Developmental Biology, University of Science and Culture, Tehran, Iran; 4 Chitsaz Lab, Department of Computer Science, Colorado State University, Fort Collins 80523, CO, USA; 5 Institute of Biomedicine of Valencia, Spanish National Research Council, Jaime Roig 11, 46010 Valencia, Spain; 6 Max Planck Institute for Molecular Biomedicine, Röntgenstrasse 20, 48149 Münster, Germany; 7 University of Saarland, FR 8.3, Biological Sciences, Genetics/Epigenetics, Campus A2.4, 66123 Saarbrücken, Germany; 8 Chemical Genomics Centre of the Max Planck Society, Dortmung, Germany; 9 Group of Computational Biology and Systems Biomedicine, Biodonostia Health Research Institute, 20014 San Sebastián, Spain

**Keywords:** mouse, ESC derivation, EMT

## Abstract

Pluripotent cells emanate from the inner cell mass (ICM) of the blastocyst and when cultivated under optimal conditions immortalize as embryonic stem cells (ESCs). The fundamental mechanism underlying ESC derivation has, however, remained elusive. Recently, we have devised a highly efficient approach for establishing ESCs, through inhibition of the MEK and TGF-β pathways. This regimen provides a platform for dissecting the molecular mechanism of ESC derivation. Via temporal gene expression analysis, we reveal key genes involved in the ICM to ESC transition. We found that DNA methyltransferases play a pivotal role in efficient ESC generation. We further observed a tight correlation between ESCs and preimplantation epiblast cell-related genes and noticed that fundamental events such as epithelial-to-mesenchymal transition blockage play a key role in launching the ESC self-renewal program. Our study provides a time course transcriptional resource highlighting the dynamics of the gene regulatory network during the ICM to ESC transition.

## Highlights

• Transcriptome data indicated a dynamic gene expression during ICM-ESC transition
• DNA methyltransferases play a prominent role in efficient ESC generation
• ESCs maintain pre-Epiblast cell identity
• EMT blockage is required for ESC derivation

## Teaser

In this article, Baharvand and colleagues provided a platform to discover influential genes and cellular events involved in transition from the ICM cells to embryonic stem cells (ESCs) by applying gene expression profiling from the ICM cells, various stages of ICM outgrowths, and early to further passages of ESCs.

## Introduction

Pluripotency is initiated in cells of the inner cell mass (ICM) and lapses shortly after implantation, coincident with the rise in lineage commitment. *In vitro* culture of ICM permits the generation of stable self-renewing pluripotent embryonic stem cells (ESCs) (Evans and Kaufman, 1981). However, ESC derivation is largely dependent upon the culture conditions. Under conventional medium, containing fetal calf serum and either feeder cells or leukemia inhibitory factor (LIF), only embryos from 129/Sv strain can efficiently give rise to ESCs and most strains of mice are refractory to ESC generation (Brook and Gardner, 1997). It is shown that the 129/Sv strain has intrinsically more preimplantation epiblast (preEpi) cells than primitive endoderm (PE) cells when compared with refractory strains such as C57BL/6 or CBA (Batlle-Morera et al., 2008). So, preventing the formation of PE cells by induction of embryonic diapause (Brook and Gardner, 1997) or use of chemical substances that inhibit Fgf4 signaling (Ying et al., 2008) led to the formation of preEpi cells with efficient capability to generate ESCs even in serum- and feeder-free culture conditions. Also, when pre-blastocyst embryos have been used for ESC derivation, it can be assumed that these embryonic stages develop mainly into preEpi cells that subsequently develop into ESCs (Nichols and Smith, 2011). But, as pluripotent preEpi cells do not exhibit self-renewability, per se, the mechanism underlying this *in vivo* to *in vitro* conversion remains controversial (Loh et al., 2015).

To address the mechanisms underlying ICM to ESC conversion in the conventional culture condition, single-cell RNA sequencing (RNA-seq) analysis showed dramatic transcriptional and epigenetic gene expression changes during ICM to ESC transition (Tang et al., 2010). These changes include the simultaneous downregulation of *Pramel5/6/7*, *Gata6*, and *Cdx2* and upregulation of *Dnmt3a* and *Hdac5*, reflecting the higher expression of epigenetic modifiers during this cell transition process (Tang et al., 2010). This study suggested that reprogramming is a major event in the cell fate conversion of ICM to ESCs. However, this mechanism may be overshadowed by the low efficiency of generating ESCs from refractory mouse strains under serum-based culture conditions, as well as the tendency of the generated cells to undergo differentiation and their propensity to heterogeneously express key naive pluripotency transcription factors (TFs) (Hassani et al., 2012).

Recent single-cell transcriptome analyses of mouse early embryos have demonstrated a high similarity between preEpi cells and ESCs grown in 2i (chemical inhibitors of MEK and GSK3) (Boroviak et al., 2015). The 2i condition supports the ground state of pluripotency, and triggers the efficient derivation and long-term maintenance of transcriptionally homogeneous ESCs. It has thus been suggested that 2i-grown ESCs are the actual self-renewing counterpart of naive preEpi cells, and that all preEpi cells are probably capable of becoming ESCs under 2i treatment (Boroviak et al., 2015).

We have recently introduced another efficient method for establishing ESCs from different refractory and non-permissive mouse strains by using PD0325901 and SB431542, chemical inhibitors of MEK, and transforming growth factor β (TGF-β) signaling pathways, respectively, which we named R2i (Hassani et al., 2014b). We showed that in the serum-free condition, R2i could support the ground state of pluripotency. Also, as well as from blastocysts, R2i could support the highly efficient ESC derivation from a wider developmental window, from single blastomeres of two to eight cell-stage mouse embryos with an efficiency of more than 2-fold higher than the 2i condition (Hassani et al., 2014a). Moreover, when cells underwent long-term passaging, they exhibited more genomic stability with R2i support compared with 2i (Hassani et al., 2014b). These salient features of R2i prompted us to evaluate the molecular mechanism(s) that underlie ICM to ESC transition. By conducting a gene expression profile at serial time points during the transition from ICM to ESCs, we discovered key regulatory genes and cellular events that are involved in this process. Besides the expression of preEpi-related genes, we found that the upregulation of DNA methyltransferases and an epithelial-to-mesenchymal transition (EMT) blockage probably have major impacts on the efficient generation of ESCs. This study reveals mechanistic insight into how transient pluripotent cells from the preimplantation embryo can perpetuate as self-renewing ESCs.

## Results

### Sampling Strategy for Gene Expression Profiling during the Transition from ICM to ESCs

**Figure 1:**
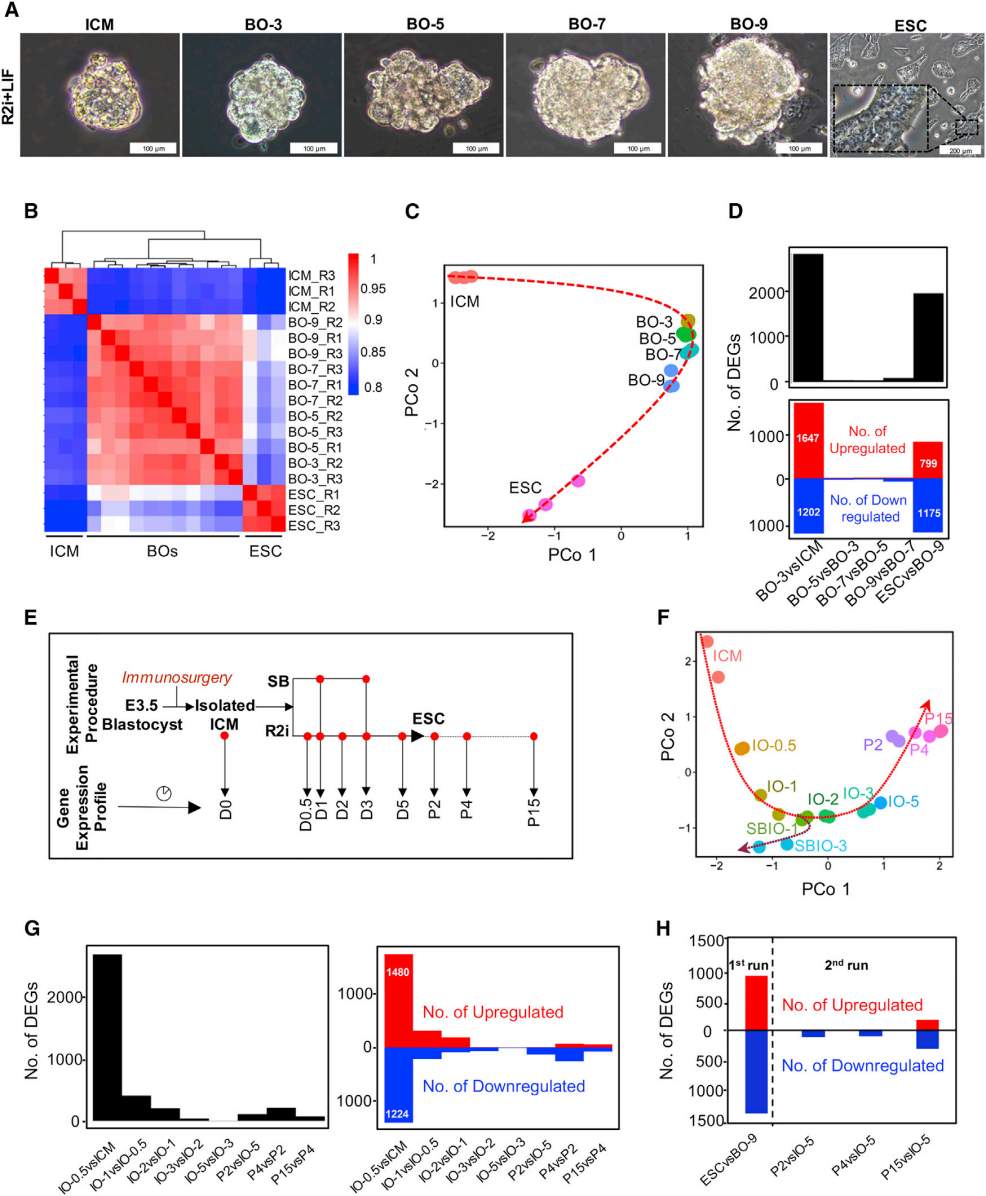
High-Resolution Transcriptome Trajectories from ICM to ESCs (A) Isolated ICM and whole-blastocyst outgrowths (BOs) on days 3, 5, 7, and 9, and ESCs (in passage 20). (B) Unsupervised hierarchical clustering of time course gene expression profiles for ESC derivation. Three independent biological replicates (nearly 20–30 ICMs and BOs for each replicate) were used except for BO-3 with two biological replicates. Transcript levels are mean-centered log_2_ scale values. (C) Principal coordinate analysis (PCoA) of time course gene expression profiles. The red arrow shows the trajectory of cell expression profiles during ESC derivation. (D) The number of differentially expressed genes (DEGs) between each of the consecutive time points. Red and blue bars indicate up- and downregulated genes, respectively. (E) Schematic illustration of the overall sample collection time points for gene expression profiling of ESC line derivation for the second run of microarray analysis. Two culture conditions were used: treatment (R2i) and control (SB). The samples (two biological replicates) included immunosurgically isolated ICMs (day 0), ICM outgrowths (IOs) on days 0.5, 1, 2, 3, and 5, and ESCs of early (P2 and P4) and late (P15) passages. (F) PCoA of the time course transcriptome profiles. The red dotted arrows show the cell-state trajectories and the bifurcation point between R2i and SB groups. (G) The number of DEGs during the indicated time points is shown on the left and significantly upregulated (red bars) and downregulated (blue bars) genes between each pair of consecutive time points are shown on the right. (H) Comparison of the number of DEGs in the first and second run of microarray for BOs versus ESCs (in the first run), and for IOs versus ESCs (in the second run).

To establish a gene expression signature throughout the course of ESC derivation, we initially collected samples from immunosurgically isolated ICMs from blastocysts at embryonic day (E) 3.5 and whole-blastocyst outgrowths (BOs) on days 3, 5, 7, and 9 post-plating, as well as from ESCs growing under R2i + LIF (R2i) conditions (Figure 1A). The mRNA extracted from these cells was pre-amplified and used for whole-genome analysis using the Illumina platform. Pairwise Pearson coefficient of the gene expression profiles of samples indicated a significant difference between the ICM cells and ESCs. Hierarchical clustering revealed three major clusters of ICMs, BOs, and ESCs (Figure 1B). Dimension reduction of gene expression data by principal coordinate analysis (PCoA) depicted a molecular trajectory that reflected the transitional cascades from ICM cells to ESCs (Figure 1C). Next, we examined the differentially expressed genes (DEGs) at the indicated time points. We found 2,849 DEGs that indicated alterations (1,647 up- and 1,202 downregulated) between ICM cells and BO-3, and 1,974 DEGs (799 up- and 1,175 downregulated) between BO-9 and ESCs (≥2-fold expression change, adjusted p ≤ 0.05) (Figure 1D). We performed an unsupervised time course of gene clustering followed by gene set analysis using the Enrichr web tool (http://amp.pharm.mssm.edu/Enrichr/; Figure S1A). We found evidence for pluripotency and the establishment of self-renewal in ESCs, as indicated by related functional annotation such as PluriNetWork and mitotic cell cycle in clusters IV and V, respectively. However, our data documented an alteration in gene expression between two major phases of the ICM to ESC transition. The first phase was detectable in the early stage of the process, between ICM cells and BO-3, whereas the second phase occurred in the late stage, between BO-9 and ESCs (Figures 1B and 1C).

We assumed that a 3-day gap between ICM cells and the first collected BOs almost hindered accurate interpretation of the extracted data. Furthermore, even though trophectoderm cells were not expanded (Figure 1A), the few such cells that existed probably resulted in gene alterations from BO-9 to ESCs. This result convinced us that a higher-resolution time course analysis from isolated ICM outgrowths (IOs) would give us better insight into the molecular cascades occurring during the cellular transition. Therefore, we collected immunosurgically isolated ICM cells and IOs on days 0.5, 1, 2, 3, and 5 post-plating (Figure 1E). In these analyses, we limited the period of study to day 5 due to a high similarity between the different time points of BO samples in the first run of the microarray. In addition, we used ESCs of different passage numbers (P2, P4, and P15) in our analyses. The negative control group for these experiments was the cultured IOs under SB + LIF (SB) conditions on days 1 and 3 post-plating (SBIO-1 and SBIO-3, respectively; Figure 1E). This control group was based on our previous observation that each component of R2i (PD or SB) in combination with LIF supports the long-term maintenance of ESCs. However, the efficiency of deriving ESCs under SB + LIF conditions was negligible or low, whereas it approximated 60% under PD + LIF (Hassani et al., 2014b). PCoA of the time course data depicted a parabolic trajectory of the ICM to ESC derivation process that bifurcated under SB conditions (Figure 1F). This analysis showed that the profile in SBIO-1 was close to the ICM to ESC trajectory, whereas SBIO-3 significantly differed from IO-3. We observed the highest number of DEGs in the sequential time points between IO-0.5 and ICM cells, at 2,704 altered genes (1,480 upregulated and 1,224 downregulated; adjusted p < 0.01; fold change >2; Figure 1G). This tangible gene alteration between ICM cells and IO-0.5 was reminiscent of the high number of DEGs between the ICM cells and BO-3 in the first run of our experiments. We observed that the alternations among consecutive samples are biologically relevant rather than a fluctuation among replicates (Figure S1B). Therefore, our study denoted a significant expression fluctuation from *in vivo* to *in vitro*, even in the short time period after this cultivation. However, the number of DEGs between late-stage outgrowths and ESCs of different passage numbers was dramatically dropped in the second set of experiments (Figure 1H). Overall, this new design for sampling provided a convenient platform for assessing the key events involved in the generation of ESCs from ICM cells.

### A Gradual Molecular Transition from ICM to ESCs

**Figure 2:**
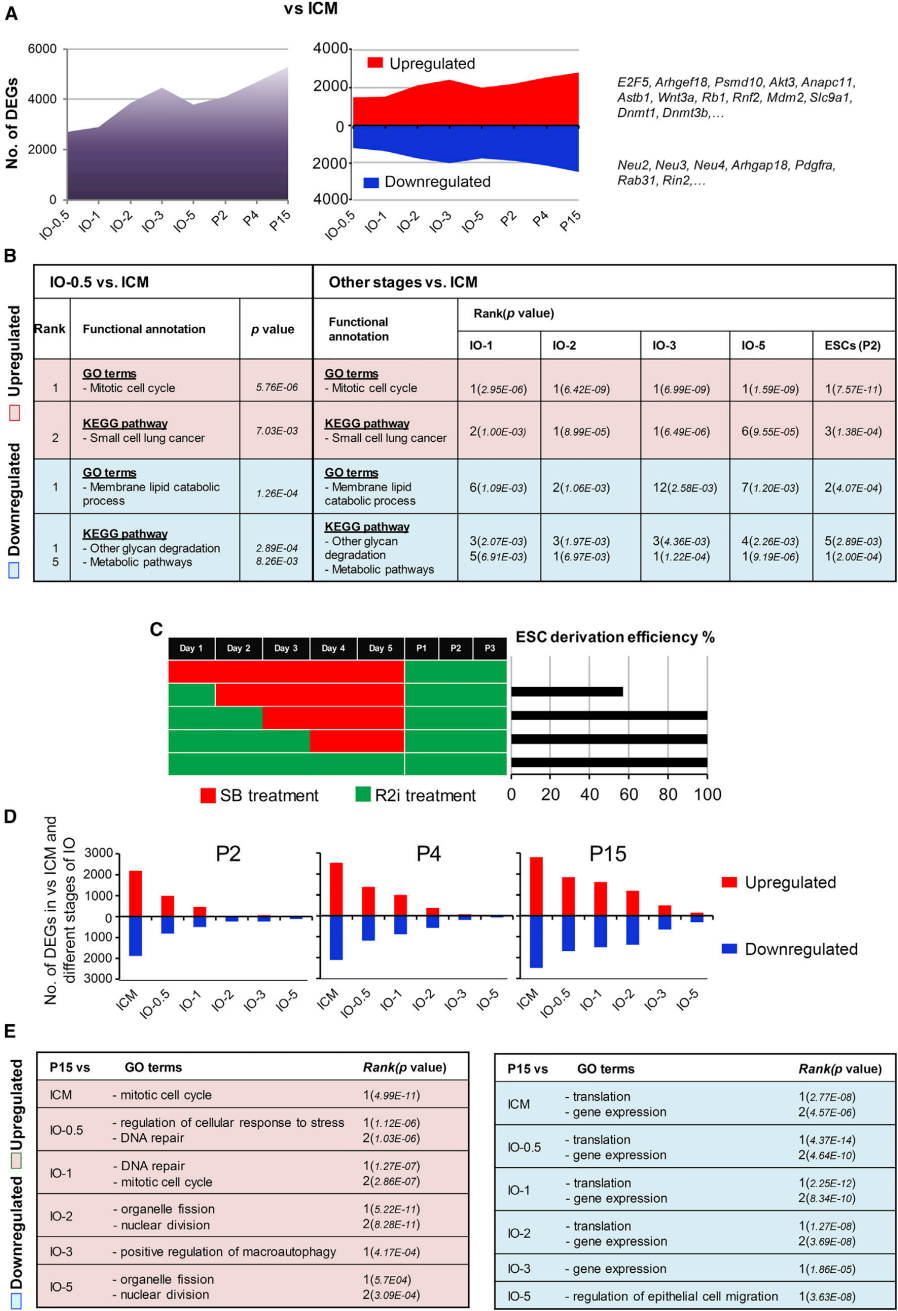
Transition from ICM to ESCs Is a Gradual Process (A) The representation of DEGs between different stages of IOs and ESCs versus ICM. (B) Functional annotation of up- and downregulated genes between IOs and ESCs of different stages versus ICM. (C) Experimental schematic and the results of time course dependency of the derived ESCs under R2i culture conditions. For each experiment (rows), the red and green bars indicate the duration of time the cells were cultured in the negative control and R2i media, respectively. The efficiency of deriving ESCs is based on the number of Nanog-positive colonies derived from ten isolated ICM. (D) The number of up- and downregulated genes between ESCs and R2i-treated IOs. (E) Functional annotation of up- and downregulated genes between P15 and IOs of different stages.

Our first priority was to uncover the reason for the tremendous difference in the number of DEGs between the ICM cells and IO-0.5. Gene set analysis showed the reasonable enrichment score for genes involved in the mitotic cell cycle and membrane lipid catabolism for up- and downregulated genes, respectively (Figures 2A, 2B, and S2A). We sought to determine whether the gene expression alteration in the first interval could cause the establishment of ESCs, or only represented a stochastic fluctuation of gene expression during *in vivo* to *in vitro* transition. Therefore, we compared the expression profile of IOs from the other designated time points with the ICM cells (Figure 2A). This analysis revealed an upward trend in the number of DEGs for IOs with an increased time point interval and the ICM cells (Figure 2A). However, most of the altered genes revealed the same functional annotation identified in the comparison between ICM cells and IO-0.5, as well as pathways for small-cell lung cancer and relevant metabolism with regard to the up- and downregulated genes, respectively (Figures 2B and S2A). This representation of biological processes during the ICM to ESC transition appeared to be consistent with the acquisition of ESC self-renewal capability.

As the early evidence of self-renewal capability was found during ICM to IO-0.5 transition (Figures 2A and 2B), we sought to determine whether ESC identity was acquired quickly after ICM expansion *in vitro*. Therefore, we examined the temporal dependency on R2i culture conditions to determine the minimal time required for the efficient derivation of ESCs. We found that 1-day treatment with R2i led to the derivation of ESCs at approximately 60% efficiency, and that 2-day treatment induced ESCs at maximal derivation efficiency (Figure 2C). This finding revealed that the gene expression changes taking place during the first days of ICM culture supported the efficient establishment of ESCs. In the next step, we compared the expression profile of ESCs of different passages (P2, P4, and P15) with ICM cells and all the IOs (Figures 2D and 2E). The results showed that, with increasing passage number, ESCs are closer to late-stage IOs than to early-stage IOs (Figures 2D and 2E). Functional analysis of DEGs between IOs and ESCs at different passage numbers indicated the upregulation of nuclear division-related genes and the downregulation of genes involved in translation and gene expression processes. However, these differences declined as the interval between ESCs and IOs was reduced (Figures 2E and S2B–S2D). Nearly similar functional annotations were enriched by analyzing the differences in gene expression between ESCs of different passages (Figure S2E). Despite the high similarity between ESCs and late-stage IOs, functional analysis for downregulated genes highlighted the importance of pathways related to epithelial cell differentiation, organ formation, extracellular matrix organization, and regulation of epithelial cell migration (Figures S2B–S2D and 2E). This result suggested that preventing the differentiation or migration of epithelial cells should be considered for successfully establishing an immortal ESC line. Therefore, it appeared that, although key changes in gene expression occurred quite quickly during the conversion of ICMs to ESCs, cells had assumed an ESC identity rather gradually. These findings suggested the existence of a distinct core regulatory circuitry for the establishment of ESCs. This circuitry would form and be active promptly during the *in vitro* ICM culture on one hand, but would induce cells to acquire ESC identity gradually on the other hand.

### Identification of Transcriptional Signature in IOs

**Figure 3:**
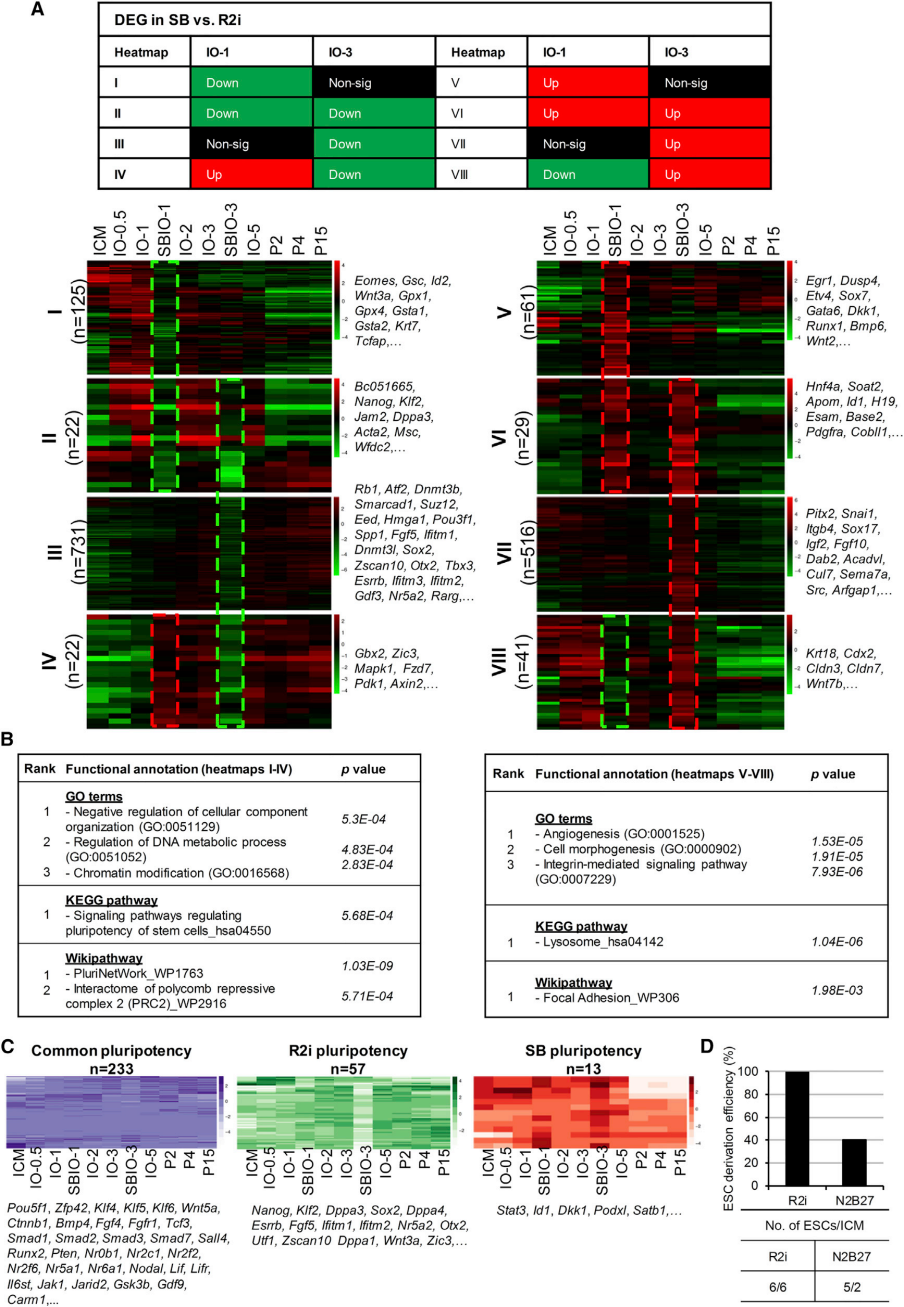
Transcriptome Signature of IOs (A) Categorization of DEGs for IOs on days 1 and 3 between the R2i and SB groups (IO-1 versus IOSB-1 and IO-3 versus IOSB-3). The upper panels show the pattern of this classification for the below heatmaps. Heatmaps I–IV represent the pattern of genes upregulated in R2i. Heatmaps V–VIII represent the pattern of genes downregulated in R2i versus SB. The related genes for each cluster are shown. Green and red dashes show significant down- and upregulated genes in SB versus R2i, respectively. (B) Functional annotation of heatmaps I–IV (R2i-specific genes) and V–VIII (SB-specific genes). The high ranking of gene ontology (GO) analysis, overrepresented KEGG pathways and WikiPathway are shown. (C) A set of pluripotency-related genes shared between R2i- and SB-treated IOs (common pluripotency) or specific to R2i pluripotency and SB pluripotency. (D) Efficiency of deriving ESCs upon *Nanog* overexpression. A Tet-On Nanog-inducible 3.5-day blastocyst from F1 hybrid × OG2 mice was used for this study.

To identify the genes that play a key role in the ICM to ESC transition process, we categorized DEGs in both the R2i and control (SB) groups. We generated the gene expression profile of the SB group at two serial time points (SBIO-1 and SBIO-3) and subsequently compared their DEGs with the R2i group at the same days (IO-1 and IO-3). The outcome consisted of eight heatmaps (Figure 3A, Table S1). Heatmaps I–IV showed the list of genes that were downregulated in at least one of the aforementioned time points in the SB versus R2i groups, while heatmaps V–VIII showed the upregulated genes (Figure 3A). Pathway enrichment analysis indicated that most genes in heatmaps I–IV were associated with signaling pathways that regulated pluripotency, chromatin modification, and DNA metabolism. Specifically, heatmap III contained the largest number of DEGs, which revealed several pluripotency markers such as *Esrrb, Sox2*, *Tbx3*, and *Rarg*, and epigenetic modifiers including *Wdr3*, *Suz12*, *Ezh2*, *Eed*, *Nr5a2*, *Dnmt3b*, and *Dnmt3l* (Figures 3A, 3B, and S3A). Thereafter, we denoted the list of genes in heatmaps I–IV as R2i-specific and downregulated in SB groups, suggesting that these genes play an important role on the road to pluripotency. In contrast, heatmaps V–VIII (SB-specific gene list) highlighted mainly developmental pathways that included cell morphogenesis, cell-cell adhesion, and protein processing. In these gene lists, the greatest numbers of DEGs represented in heatmap VII contained some key upregulated factors such as *Pitx2*, *Snai1*, *Itgb4*, *Sox17*, *Igf2*, *Fgf10*, and *Dab2*, suggesting that the SB-treated IOs exited from the path of pluripotency (Figures 3A, 3B, and S3A).

Interestingly, our result depicted that the upregulation of some relevant pluripotency-related markers was observed in the SB-specific gene list, which included *Stat3*, *Dusp9*, and *Id1*, downstream of LIF and BMP4, respectively. This finding raised the question of whether TGF-β inhibition could sustain the expression of some pluripotency-related genes during the ICM to ESC transition. To address this question, we classified the expression profile of a comprehensive list of pluripotency-related genes (366 genes extracted from the PluriNetWork and the literature; Table S2) into common pluripotency, R2i-specific pluripotency, and SB-specific pluripotency groups (Figure 3C). A total of 233 well-known TFs that regulated pluripotency was put in the common pluripotency list, which included *Pou5f1* (*Oct4*), *Zfp42* (*Rex1*), *Klf4*, *Ctnnb1* (β*-Catenin*), and *Sall4*. In addition, 13 of 366 genes were in the SB-specific pluripotency list, which included *Stat3*, *Id1*, *Dkk1*, and *Podxl*. This analysis demonstrated that TGF-β inhibition, like fibroblast growth factor (FGF) signaling inhibition, plays a pivotal role in supporting the dynamic expression of pluripotency-related markers, while SB alone was insufficient for ESC derivation. It has been proposed that some of the pluripotency factors induce differentiation and play a central role in the determination of different cell states by orchestrating different gene expression profiles (Loh et al., 2015). Consistent with this notion, co-occupation of various sites with Smad2/3 and different master TFs has been found to direct different responses in ESCs, myoblasts, and B cells (Mullen et al., 2011). In contrast, R2i-specific pluripotency markers, such as *Nanog*, *Klf2*, *Dppa3*, *Sox2*, *Esrrb*, *Utf1*, and *Nr5a2*, have unveiled key regulators in the path to efficiently deriving ESCs from ICM. We showed that 5 (*Esrrb*, *Gbx2*, *Sox2*, *Klf2*, and *Nanog*) of 12 essential TFs for naive pluripotency (Dunn et al., 2014) were in the R2i-specific pluripotency list, suggesting that they play key roles during ESC derivation (Figure S3B). To test this finding, we overexpressed *Nanog* in the ICM cells of E3.5 blastocysts obtained from *Nanog*-inducible mice in order to generate ESCs in N2B27 medium in the absence of SB or PD, resulting in 40% ESC derivation efficiency (Figure 3D). These data indicated that our strategy to classify the expression of pluripotency markers during the expansion of ICM could point to the key players involved in ESC derivation.

### Transition in DNA Methylation Level during the ICM to ESC Conversion

**Figure 4:**
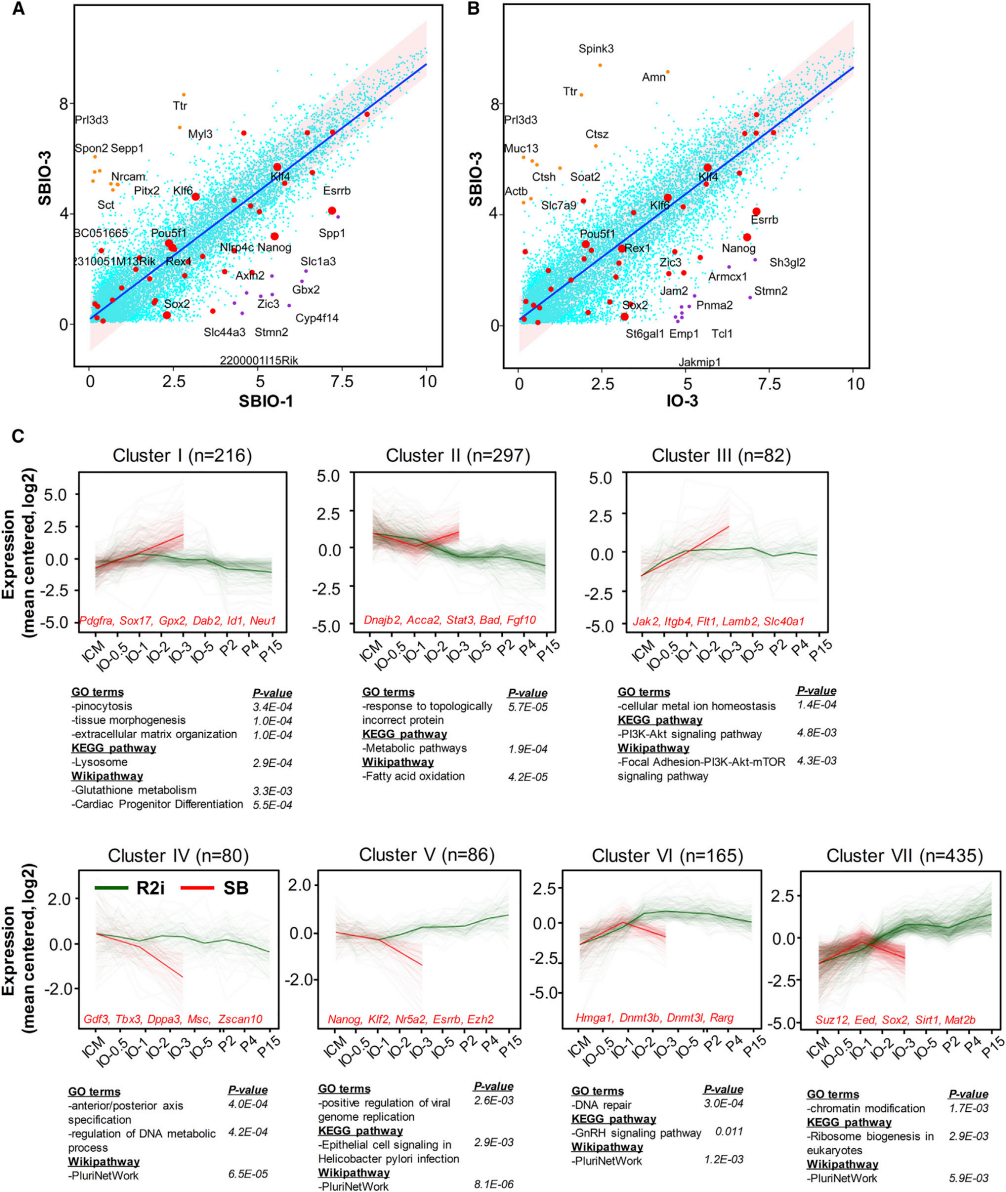
Day 3 Outgrowths Represented the Highest Difference between R2i and SB Groups (A and B) Scatterplots showing the gene expression profiles of (A) SBIO-3 versus SBIO-1 and (B) SBIO-3 versus IO-3. The trend is shown as a blue line. The pink ribbon shows genes that have less than 2-fold expression changes between the two samples (p < 0.05). (C) Unsupervised clustering of gene expression for DEGs for IOs on day 3 between treatment (R2i) and control (SB) groups. Clusters I–III represent the pattern of genes that are downregulated in IO-3 versus SBIO-3. Clusters IV–VII represent the pattern of genes that are upregulated in IO-3 versus SBIO-3.

**Figure 5:**
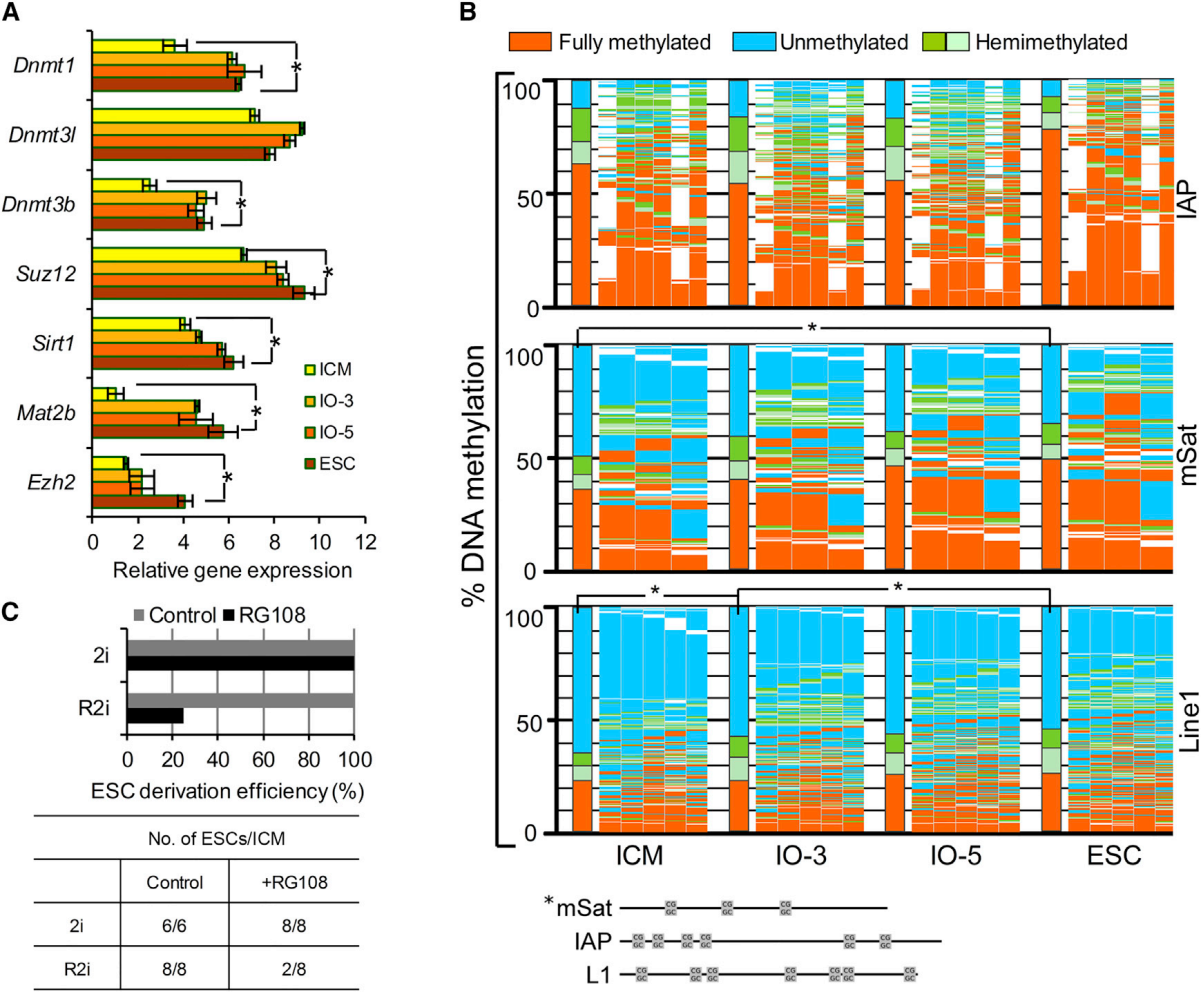
DNA Methylation Changes during ESC Derivation (A) Time course expression of epigenetic modifier genes during ESC derivation using qRT-PCR. Three biological replicates were used for this experiment. Error bars represent the SD. Here, ^∗^ represents significant DEGs between ICM and ESCs, p ≤ 0.01. Statistical analysis was performed by one-way ANOVA and *post hoc* Tukey test. (B) Hairpin-bisulfite amplicon sequencing of CpG dyads at retrotransposable elements (L1Md_tf, IAP-LTR1, and mSat). The bars sum up the DNA methylation status of all CpG dyads. The map next to the bar represents the distribution of methylated sites. Each column shows neighboring CpG dyads and each line represents one sequence read. The reads in the map are sorted first by fully methylated sites, then by hemi-mCpG dyads. Red, fully methylated; light green and dark green, hemi-mCpG; blue, unmethylated CpG. Two biological replicates for IAP-LTR1 and mSat and one biological replicate for L1Md_tf were used and ^∗^ represents significant difference of DNA methylation between ICM, IOs, and ESCs, p ≤ 0.01. The numbers of reads and methylation state are shown in Table S3. (C) Efficiency of ESC derivation upon inhibition of DNA methyltransferases by using RG108 in R2i- and 2i-treated IOs.

As R2i-specific pluripotency markers significantly presented in day 3 (Figures 4A and 4B), we sought to classify the gene expression patterns by performing an unsupervised time course clustering on DEGs between SBIO-3 and IO-3 (Figure 4C). Clusters I–III represented the upregulated genes in SBIO-3 compared with IO-3, which exhibited different expression patterns in the R2i-treated group. Gene ontology analysis highlighted the increased activities of several biological processes that were associated mainly with tissue morphogenesis, metabolic pathways, and focal adhesion (Figure 4C). In contrast, clusters IV–VII represented downregulated genes in the SBIO-3. Gene enrichment analysis revealed that the genes in clusters IV–VII were associated with PluriNetWork, the DNA metabolic process, and chromatin modification. Clusters IV (80 genes) and V (86 genes) contained some key TFs for pluripotency (*Tbx3*, *Esrrb*, and *Nanog*), which were most likely expressed in a steady-state pattern from ICM cells to ESCs. Clusters VI (165 genes) and VII (435 genes) consisted of parabolic and gradually upregulated patterns of genes from ICM cells to ESCs, which included some epigenetic modifiers such as *Dnmt3b*, *Dnmt3l*, *Chd8*, *Mtss1, Suz12*, *Eed*, *Wdr3*, and *Mat2b* (Figure 4C). To better understand the expression dynamics of epigenetic modifier genes during the derivation of ESCs, we conducted qRT-PCR analysis to quantify the expression of *Dnmt1*, *Dnmt3b*, *Dnmt3l*, *Sirt1*, *Ezh2*, *Suz12*, and *Mat2b* (Figure 5A). This experiment confirmed that the expression of *Dnmt1*, *Dnmt3b*, and their partners, was upregulated in this cell transition process. To examine the DNA methylation pattern, we measured the methylation level of the individual CpG sites for three classes of repetitive elements by deep hairpin-bisulfite sequencing. As approximately 40% of the genome consists of repetitive elements, we selected major Satellites (mSat), the 5′ UTR of L1Md_Tf (L1), and a class of LTR-retrotransposons (IAP-LTR1) (Arand et al., 2012). Our data showed that the DNA hyper-methylation during ESC derivation was due mainly to increased amounts of hemi-methylation at CpG positions (Figure 5B). We observed the highest amount of non-CpG methylation at mSat in R2i-treated IOs (Figure S4; Table S3). Non-CpG methylation depended on the presence of Dnmt1 mediated by Dnmt3a/3b in ESCs under serum conditions (Arand et al., 2012). This finding showed that early changes in DNA methylation probably occurred in tandem with an increased activity in *de novo* methyltransferases.

Next, we sought to determine the impact of increased global DNA methylation levels on the transition between the *in vivo* to *in vitro* pluripotent cell states. Inhibition of DNA methyltransferases by RG108 led to reduced efficiency of deriving ESCs under R2i conditions, but had no adverse effect under 2i conditions (Figure 5C). It had previously been reported that 2i could induce global hypomethylation (Ficz et al., 2013, Leitch et al., 2013); however, our results indicated the upregulation of *de novo* methyltransferases for the efficient establishment of ESCs in R2i. Unexpectedly, this finding implied that blockage of the ERK pathway, in parallel with TGF-β inhibition, led to a high expression of epigenetic modifiers and DNA methylation-related genes in the transition from ICM to ESCs. This analysis revealed a major difference between 2i- and R2i-grown ESCs, suggesting the existence of a different pluripotency circuitry of these two naive representative conditions.

### Blocking EMT Is Required for ESC Derivation

**Figure 6:**
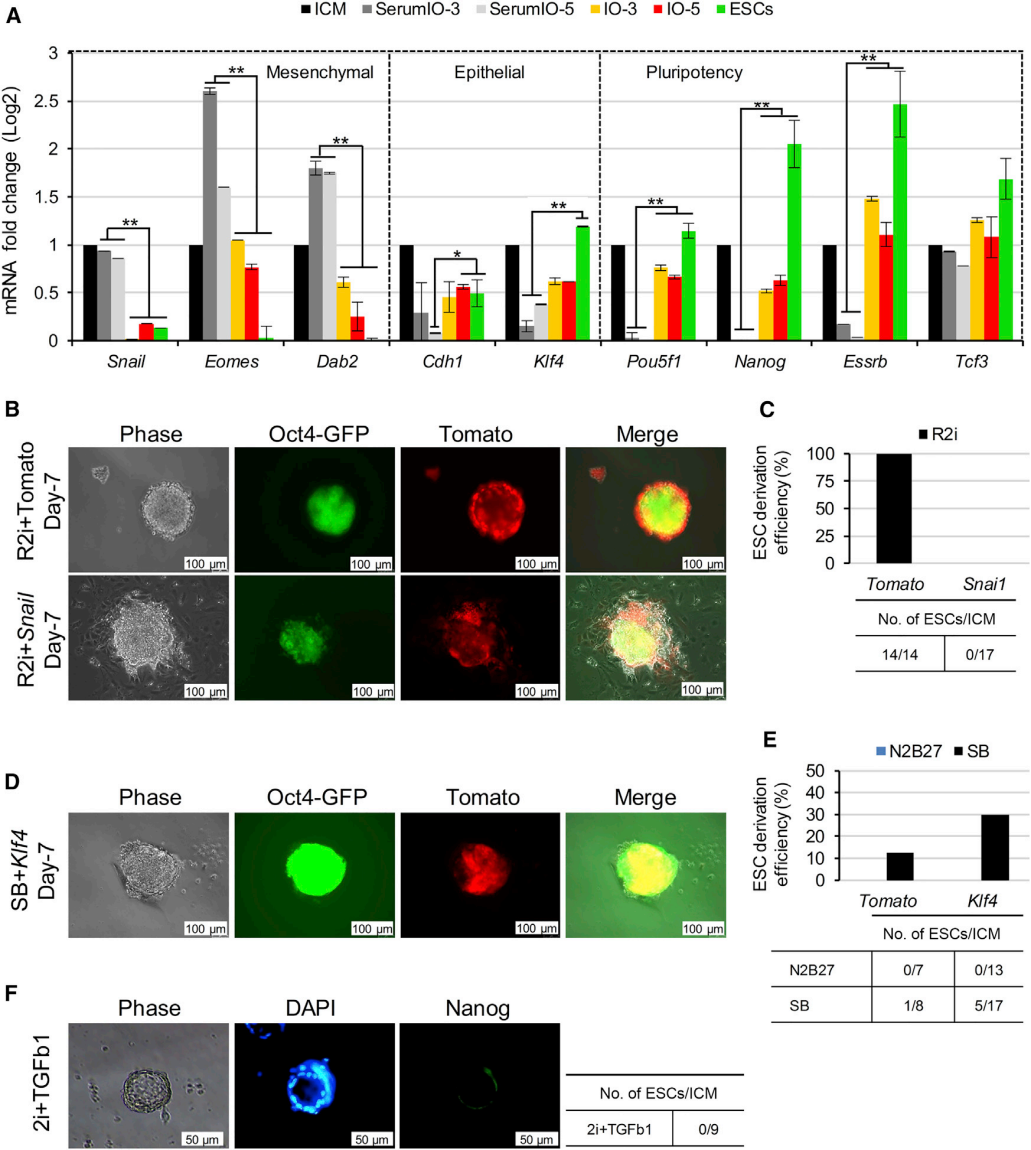
Blocking EMT Is Required for ESC Derivation (A) Time course expression of EMT and MET gene markers was measured by qRT-PCR in R2i- and serum + LIF-treated IOs. All data were calibrated to ICM, which is considered 1. Error bars reflect SD of results derived from three biological replicates. Here, ^∗^ represents significant deferentially expressed genes in R2i- and serum + LIF-treated IOs as well as ESCs. ^∗^p ≤ 0.05, ^∗∗^p ≤ 0.01, error bar, ± SD. (B) Overexpression of *Snail*-2A-tdTomato in OG2 ICM, which contain an *Oct4*-GFP reporter transgene, with R2i treatment. A construct with tdTomato alone was used as a negative control. Induction of EMT through the forced expression of *Snail* impaired ICM to ESC transition. tdTomato expression correlates with the transduction rate and expression of the gene of interest. (C) Efficiency of ESC derivation upon *Snail* overexpression. (D) Overexpression of *Klf4*-2A-tdTomato on E3.5 ICM (B6 × C3H) F1 in N2B27 supplemented with SB + LIF prevents EMT and supports the derivation of ESC lines. (E) Efficiency of ESC derivation upon *Klf4* overexpression on SB-treated IOs. (F) The impact of TGF-β1 on the generation of ESCs was assessed in 2i-treated IOs after NANOG immunostaining. Blue, DAPI.

Our gene expression profile analysis highlighted a difference between ESCs and IOs in epithelial differentiation and migration (Figures 2E and S2B–S2D). Interestingly, a gene set enrichment analysis between DEGs from R2i- and SB-treated IOs displayed an enrichment of EMT-related genes (Figure S5A). Thus, we sought to assess whether EMT might play a critical role in ESC derivation. To this end, the expression levels of genes involved in EMT and mesenchymal-to-epithelial transition (MET), such as *Snail*, *Eomes*, *Dab2*, *Cdh1*, and *Klf4*, as well as key pluripotency genes, were analyzed in R2i-treated IOs using qRT-PCR, and serum + LIF-treated IOs were used as a negative control (Figure 6A). Our results showed an upregulation of epithelial markers and a downregulation of mesenchymal markers in R2i-treated IOs compared with the negative control. Indeed, *Snail*, *Eomes*, and *Dab2* exhibited higher expression levels in the presence of serum, correlating with a decrease in ESC derivation efficiency and an increase in differentiating IOs (Figure S5B). Next, we decided to ascertain whether blocking EMT is required for ESC derivation. To this end, we overexpressed *Snail*-2A-Tdtomato (EMT inducer) and *Klf4*-2A-Tdtomato (MET inducer) in R2i- and SB-treated IOs. In the presence of Snail, IOs started to differentiate and ESCs could not be derived under R2i conditions (Figures 6B and 6C). In contrast, 14 of 14 ESC lines were established from R2i-treated IOs overexpressing Tdtomato alone as a control. Conversely, *Klf4* overexpression led to a 30% efficiency in ESC derivation in SB-treated IOs (Figures 6D, 6E, and S5C), whereas no ESC lines were derived in the Tdtomato control under the same conditions. Finally, we noticed that supplementation of 2i with TGF-β1 impeded the transition from ICM cells to ESCs (Figure 6F). Taken together, these findings demonstrate the crucial role of EMT blockage for the ICM to ESC transition. Contrary to what was expected, we showed that TGF-β inhibition induced EMT, while with the synergistic effect of MEK inhibition blocked EMT. Overall, our data demonstrate that blocking EMT is an essential step in the ESC derivation process. When EMT blocking occurs, a TGF-β inhibitor (SB) combined with LIF could support the long-term maintenance of ESC self-renewal (Hassani et al., 2014b).

### R2i Could Maintain preEpi Cell Identity in IOs

**Figure 7:**
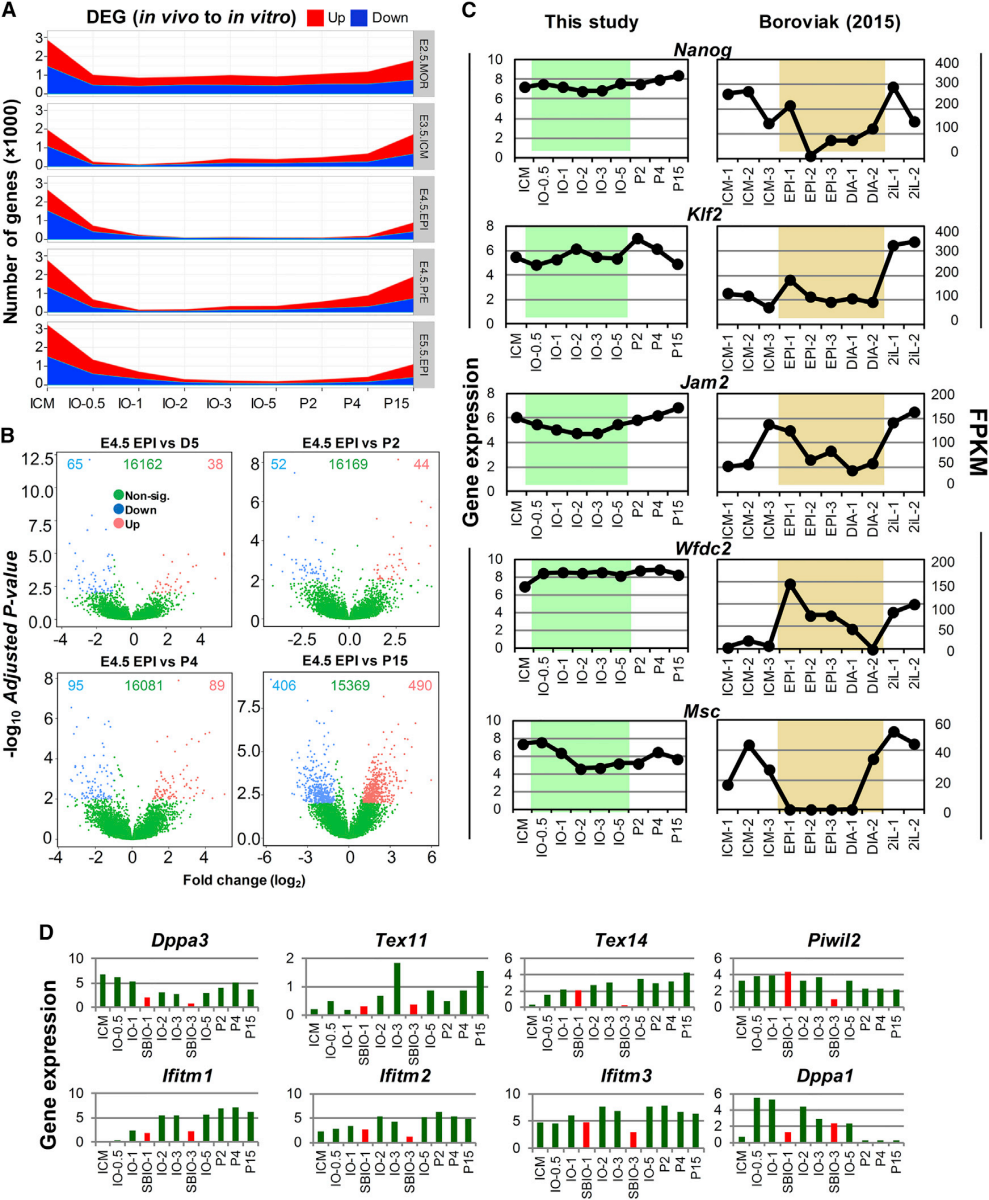
R2i-Treated IOs Exhibited preEpi Cell Characteristics (A) DEGs between *in vivo* embryonic samples (Boroviak et al., 2015) and ICM to ESC time course samples for this study (≥2-fold expression change, adjusted p ≤ 0.01). (B) Volcano plots show fold change in gene expression (x axis, log_2_ fold change, adjusted p ≤ 0.01) between E4.5 preEpi cells (Boroviak et al., 2015), IO-5, and ESCs of different passages (this study). The y axis is the negative log_10_ of Benjamini-Hochberg adjusted p value. Blue, green, and red dots represent downregulated, non-significant, and upregulated genes, respectively. (C) The comparability of the gene expression for some heatmap II-specific genes (from Figure 3) between *in vitro* IOs (this study) and single-preEpi cells (EPI1-3) or single-diapaused cells (DIA1-2; Boroviak et al., 2015). (D) The expression of PGC-related genes in R2i- and SB-treated IOs.

We also conducted a parallel transcriptional study on the development of ICM cells in both *in vitro* and *in vivo* conditions. Recently, Boroviak et al. (2015) reported single-cell RNA-seq data for the lineage-specific profile of naive pluripotency in early embryogenesis. However, those authors had adopted their protocol to small group of cells, instead of single cells, to be able to detect scarce transcripts, which provided a good substrate for comparison with our bulk transcriptome data. We observed a range of different early embryo lineage-feature gene expressions, from the ICM to post-implantation cells in R2i-treated IOs (Figure 7A; Table S4). Our study revealed a maximum similarity of different IOs and early-passage ESCs (P2 and P4) to E4.5 preEpi cells. Late-passage ESCs (P15) showed slightly less similarity to the E4.5 preEpi cells (Figure 7B), while functional analysis of up- and downregulated genes in this comparison highlighted the similar pathways (Figure S6A) that were enriched between P15 ESCs and different IOs (Figures 2E and S2D).

A comparison of R2i and SB treatment revealed that the number of ICM-related genes decreased in R2i- versus SB-treated IOs, while the number of gene markers related to preEpi and postEpi cells increased (Figure S6B). This analysis indicated the exclusive expression of diapaused-specific markers, such as *Tcfap2c*, *Gbx2*, *Utf1*, *Sox2*, *Esrrb*, *Tbx3*, *Klf2*, *Nanog*, *Dppa3*, and *Msc*, in R2i-specific clusters (Figures S6B and S6C). We also found that the expression of nearly one-third of the genes in heatmap II in Figure 3A were comparable with preEpi- and diapaused-related indicators, suggesting that the expression of these genes is important throughout the entire process of ESC derivation (Figure 7C).

We then compared our finding with a previously reported dynamic single-cell transcriptional dataset for identifying markers during the preEpi and PE bifurcation of the ICM (Gerovska and Arauzo-Bravo, 2016). We found that R2i-treated IOs noticeably represented the expression profile of E4.5 preEpi cells, while SB-treated IOs showed the different expressed genes such as *Sox17*, *Sox7*, and *Gata6*, which indicated an expression pattern of E4.5 PE (Figures S6D and S6E). In particular, one-third of genes in cluster VI, which were downregulated in R2i versus SB on both days 1 and 3, represented the PE transcriptome profile. A comparison of different staged IOs and early-stage embryos revealed significantly expressed PGC markers, which included *Ifitm3*/*Fragilis*, *Dppa3*/*Stella*, *Zp3*, *Ifitm1*, and *Ifitm2* (Boroviak et al., 2015, Gerovska and Arauzo-Bravo, 2016), as well as *Tex11*, *Tex14*, *Piwil2*, and *Dppa1* (Figure 7D). This observation not only elucidated the preEpi cell origin of ESCs but also magnified the role of PGC-related genes during the ICM to ESC transition. Overall, this dataset depicted that ICM-derived ESCs proceeded through the preEpi stage, and that the successful derivation of ESCs relied on FGF inhibition. However, epigenetic modifications and EMT blockage were required for the perpetual self-renewal capability of preEpi cells.

## Discussion

An optimized regimen is required for dissecting the molecular mechanism of ESC derivation. Dual inhibition of FGF and TGF-β creates high genomic stability and an efficient ICM to ESC transition (Hassani et al., 2014b). We have employed this system to produce a temporal transcriptome profile. In addition, a negative control group (SB), which not only maintains self-renewal but also has similar components with R2i, helped us to develop an appropriate strategy for detecting regulatory genes that play a key role in ESC derivation.

This analysis has identified a group of significantly expressed TFs and epigenetic modifiers in the ICM, different IOs, and ESCs. Our result was consistent with previously reported ICM-specific genes such as *Pramel4*, *Pramel5*, and *Pramel7* (Tang et al., 2010). In a systemic data analysis, we observed approximately 3,000 DEGs between the ICM and day 0.5 (Figure 2A). Instead of a fluctuated gene expression stemming from the *in vivo* to *in vitro* transition, we observed that these large numbers of DEGs could establish the path toward the generation of ESCs. We demonstrated that only 2 days were needed for deriving ESCs at maximal efficiency under R2i conditions (Figure 2C). However, we have suggested that ICM cells do not suddenly assume an ESC identity; the longer the cultivation period, the more likely it is that the cells will acquire ESC characteristics. We observed that, while the number of DEGs decreased between the late-stage IOs and established ESCs, ESCs showed downregulation of the EMT biological process compared with these IOs (Figure 2E). Despite the expression of common pluripotency factors, SB-treated IOs exited from pluripotency likely because they followed the EMT program. Our results are consistent with this notion, and have suggested that the ectopic expression of *Snail*, an EMT inducer, could impede the establishment of ESCs (Figure 6B). Normally, the corresponding protein presents quickly after the onset of gastrulation (Hemavathy et al., 1997). However, *Snail* surprisingly presents in SB-treated IOs as a downstream protein of the TGF-β signaling pathway. This data has illustrated that inhibition of TGF-β without suppression of FGF signaling could not block EMT and allow embryonic cells to exit from pluripotency. It has been reported that MEK suppression activates Klf4 through a phosphorylation pathway (Kim et al., 2012) and consequently induces MET (Chen et al., 2011). Accordingly, we observed that forced expression of *Klf4* in SB-treated IOs led to the generation of ESCs (Figure 6D). We also detected the downregulation of epithelial markers (*Cdh1* and *Klf4*) and the upregulation of mesenchymal markers (*Snail*, *Eomes*, and *Dab2*) under conventional conditions, i.e., serum + LIF (Figure 6A). Therefore, we concluded that the lack of EMT blockage was the main reason for the inefficient generation of ESCs from a refractory cell strain under serum or SB culture conditions. When EMT blockage occurs, SB or serum could support the long-term maintenance of ESC self-renewal.

We believe that the strategies developed in this study can help identify the most important gene regulators for deriving pluripotent ESCs. Based on significant changes in gene expression patterns and morphological changes between treatment (R2i) and the control (SB) groups on day 3 (Figure 4), we postulated that DEGs between these two groups was crucial for ICM to ESC transition. The data showed that pluripotency factors such as *Pou5f1*, *Zfp42*, *Klf4*, *Sall4*, *Ctnnb1*, and *Klf6* were not significantly altered between the treatment and control groups up to the third day of ESC derivation. We classified these 233 pluripotency markers as common pluripotency factors. However, the pluripotency markers that were significantly upregulated in R2i compared with SB, including *Nanog*, *Sox2*, *Klf2*, *Esrrb*, *Gbx2*, *Jam2*, *Dppa3*, *Msc*, and *Wfdc2*, could be the genes driving the establishment of ESCs (Figure 3C). Of all the TFs that play an essential role in maintaining pluripotency (Dunn et al., 2014), half were included in the R2i-specific gene list (Figure S3B). Therefore, we proposed that the overexpression of these genes, such as *Nanog*, would undermine the necessity of a special regimen, such as R2i and 2i (Figure 3D).

In this study, we also found a dramatic difference in the DNA methylation patterns between ICM cells and ESCs. The passive and active demethylation of maternal and paternal genomes resulted in a hypomethylated epigenetic landscape for ICM cells (Liao et al., 2015). It has been shown that ESCs have higher DNA methylation levels than ICM cells under conventional conditions, as opposed to 2i conditions (Leitch et al., 2013). DNA methylation and DNA methyltransferases expression have been broadly implicated in early embryo development and long-term maintenance of pluripotent cells (Arand et al., 2012, Ficz et al., 2013, Habibi et al., 2013, Marks et al., 2012, Smith et al., 2012), but the exact epigenetic events that occur during the derivation of ESCs have remained elusive (Tang et al., 2010). Here, we have observed that the expression of epigenetic modifier genes, such as *Dnmt3b*, *Dnmt3l*, *Chd8*, *Mtss1*, *Suz12*, *Eed*, *Wdr3*, and *Mat2b*, was significantly increased in the intermediate stages of ESC derivation, and followed an upward trend during the course of the culture (Figure 4C). Next, we measured the level of DNA methylation in the CpG dyads of IAP-LTR1 and L1Md_tf retrotransposable elements and mSat. Consistent with the results of previous studies (Ficz et al., 2013, Habibi et al., 2013, Smith et al., 2012), this entire dataset has highlighted the role of epigenetic modifiers and DNA methylation, pointing toward a reconstruction process that shapes a different epigenetic landscape for ESCs. In contrast with previous observations (Ficz et al., 2013, Okashita et al., 2014), blockage of the ERK pathway was probably required for a high expression of epigenetic modifiers and DNA methylation-related genes during ICM to ESC transition. Interestingly, we found that RG108-mediated inhibition of DNA methylation impeded the efficient derivation of ESCs in the presence of R2i, yet had no adverse effects under 2i conditions. This analysis highlighted a major difference between the R2i and 2i conditions in the regulation of pluripotency. It has previously been demonstrated that the augmented BMP4 signaling pathway plays a key role in R2i pluripotency, whereas it does not make a difference in the 2i condition (Gomes Fernandes et al., 2016, Hassani et al., 2014b). Despite the high degree of similarity between the R2i and 2i conditions, as well as the strength of R2i in supporting pluripotency, i.e., in establishing and maintaining ESCs from single blastomeres (Hassani et al., 2014a) and embryonic germ cells from the PGCs of mice and rats (Attari et al., 2014, Mohammadi et al., 2015), these two conditions work in different ways.

To determine the state of R2i pluripotent cells in comparison with *in vivo* pluripotent cells, we compared our transcriptome data with that of early embryonic cells (Figure 7). Although a considerable number of preEpi- and PE-specific markers demonstrated concurrent expression patterns in both the R2i- and SB-related clusters, the enrichment of substantial preEpi- and PE-specific genes in the R2i and SB groups, would suggest a specified path for the generation of ESCs from ICM cells. We observed that R2i treatment could support the expression of diapause- and PGC-related genes, which were associated with the preEpi cell state. A comparison of 2i-grown cells with early embryonic cells indicated that there was greater similarity with them and E4.5 preEpi cells (Boroviak et al., 2015). In this study, we also demonstrated that R2i could sustain the preEpi-specific gene expression during the process of establishing ESCs from the ICM. IOs and early-passage ESCs were more similar to preEpi cells than were P15 ESCs. This was consistent with data by Boroviak et al. Therefore, despite certain differences between 2i and R2i for maintaining pluripotency, both conditions produced ESCs that exhibited high similarity to E4.5 preEpi cells. However, our results demonstrated that the establishment of ESCs requires a reprogramming phenomenon such as DNA methylation regulation and EMT blockage.

To the best of our knowledge, this is the first study reporting that EMT prevention is required for the derivation of ESCs from the ICM. In addition to the maintenance of preEpi-specific marker gene expression, prevention of TGF-β and MAPK under R2i conditions tended to inhibit the EMT process in normal development of the early embryo and to maintain pluripotency *in vitro*. From the perspective of human ESCs, it would be of tremendous interest to investigate the role of EMT or cell adhesion in the derivation of primed and naive human ESCs.

## Experimental Procedures

### ESC Derivation and Sample Collection

For ESC derivation, blastocysts/isolated ICMs were prepared as described in the Supplemental Experimental Procedures. The isolated ICMs were washed twice in PBS, and then selected for microarray analysis. ESCs were derived by transferring blastocysts/isolated ICMs on gelatin-coated plates (0.1%, Sigma-Aldrich) that contained N2B27 defined medium supplemented with R2i (which included 1 μM PD0325901 [Stemgent] and 10 μM SB431542 [Sigma-Aldrich]) and 1,000 U/mL LIF (ESGRO, Millipore). This time was designated as day 0. For the first microarray analysis, the samples included isolated ICMs (day 0), BOs on days 3, 5, 7, and 9 (BO3–9) after plating, and ESCs on passage 20, in three biological replicates. We chose and pooled approximately 20–30 isolated ICMs and BOs for each biological replicate.

For the second microarray analysis, we collected new samples in a time resolution experiment that included immunosurgically isolated ICM, IOs, on days 0.5, 1, 2, 3, and 5 (IO0.5–5), and passages 2, 4, and 15 of ESCs (P2–15). In addition, IOs which were cultivated in N2B27 supplemented with SB431542 + LIF on days 1 (SBIO-1) and 3 (SBIO-3) considered as the negative controls. Approximately 30–40 ICM or IOs were picked and pooled in two biological replicates.

## Author Contributions

M.T., S.N.-H., and A.S. developed the study. N.T. performed some of the EMT experiments and discussed the EMT results. K.A. discussed the EMT results. J.A. and J.W. performed the DNA methylation experiments. B.G. discussed the gene expression results. D.S. assisted with animal and embryo manipulations. M.A. analyzed the microarray data. M.P. performed the time course derivation and supplemental experiments. H.G. supervised the project, provided financial support, and discussed the results. H.S. designed the experiments, discussed the results, and approved the manuscript. H.B. provided financial support, designed and analyzed experiments, discussed the results, and approved the manuscript.

## Accession Numbers

Gene profiling data was submitted to the GEO repository with accession number GEO: GSE87793.

## Supplemental Information

Document S1. Supplemental Experimental Procedures, Figures S1–S6, and Table S3

Table S1. The List of Genes and the Expression Value of Represented Heatmaps Shown in Figure 3A, Related to Figure 3

Table S2. The List of Common Pluripotency, R2i Pluripotency, and SB Pluripotency-Related Genes Based on Published Data, Related to Figure 3

Table S4. Comparison of the Expressed Genes for the *In Vivo* Embryonic Sample (Boroviak et al., 2015) and *In Vitro* Cultured ICM to ESC Samples of This Study, Related to Figure 7 Up, Up-regulated; Down, Down-regulated; NS, Non-significant

Document S2. Article plus Supplemental Information
