# Supplementary material for: Blockage of the Epithelial-to-Mesenchymal Transition Is Required for Embryonic Stem Cell Derivation
Source: Stem Cell Reports. 2017 Sep 14;9(4):1275–90. doi: 10.1016/j.stemcr.2017.08.006 (PMC5639184; doi:10.1016/j.stemcr.2017.08.006)
Supplement: Document S1. Supplemental Experimental Procedures, Figures S1–S6, and Table S3 [file mmc1.pdf]

Stem Cell Reports, Volume *9*

Supplemental Information

Blockage of the Epithelial-to-Mesenchymal Transition Is Required for Embryonic Stem Cell Derivation

Mehdi Totonchi, Seyedeh-Nafiseh Hassani, Ali Sharifi-Zarchi, Natalia Tapia, Kenjiro Adachi, Julia Arand, Boris Greber, Davood Sabour, Marcos J. Araúzo-Bravo, Jörn Walter, Mohammad Pakzad, Hamid Gourabi, Hans R. Schöler, and Hossein Baharvand

Supplemental Information

Blockage of the Epithelial-to-Mesenchymal Transition Is Required for Embryonic Stem Cell Derivation

Mehdi Totonchi, Seyedeh-Nafiseh Hassani, Ali Sharifi-Zarchi, Natalia Tapia, Kenjiro Adachi, Julia Arand, Boris Greber, Davood Sabour, Marcos J. Araúzo-Bravo, Jörn Walter, Mohammad Pakzad, Hamid Gourabi, Hans R. Schöler, Hossein Baharvand

INVENTORY OF SUPPLEMENTARY INFORMATION

I. Supplementary Data

Figure S1, related Figure 1.

Figure S2, related Figure 2.

Figure S3, related Figure 3.

Figure S4, related Figure 5.

Figure S5, related Figure 6.

Figure S6, related Figure 7

Table S1, related Figure 3

Table S2, related Figure 3

Table S3, related Figure 5

Table S4, related Figure 7

II. Supplemental Experimental Procedure

III. Supplemental References

Supplemental Figures

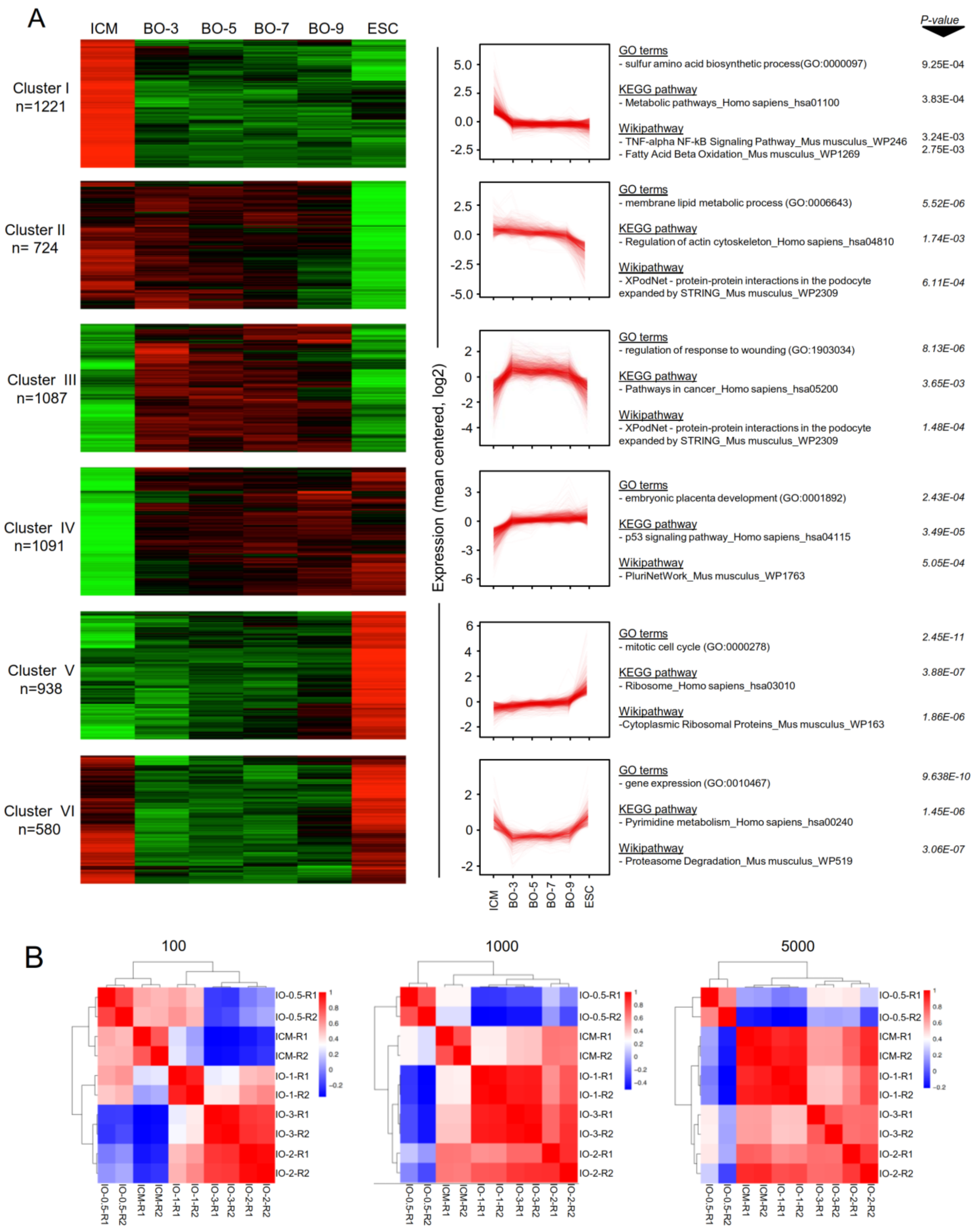

Figure S1. Temporal morphology and time-course transcriptome profiling of ESC derivation, related to Figure 1.

(A) Unsupervised hierarchical clustering and functional annotation of time-course gene expression profiles for ESC derivation. We used three independent biological replicates, except for BO-3, which consisted of two biological replicates. Between 20 and 30 ICMs were used for each replicate. Transcript levels are mean-centered log2 scaled values.

(B) Spearman correlation heatmap analysis among the first timepoints (ICM, IO-0.5, IO-1, IO-2 and IO-3) to measure the differences among replicates vs. timepoints. Since the expression levels of a minority of the genes have significant alternations among samples, this analysis was limited to 100, 1000 and 5000 microarray probes that had the maximum expression variance among these samples. The biological replicates are clustered together in this analysis, which showed the differences among consecutive samples are larger than the observed fluctuation among replicates. This shows that the alternations among consecutive samples are biologically relevant.

vs ICM

A

Up-regulated

IO-0.5

GO Term	*P-val.*
mitotic cell cycle (GO:0000278)	5.76E-06
**KEGG pathway**	***P-val.***
Ubiquitin mediated proteolysis_Homo sapiens_hsa04120	5.98E-04
Adrenergic signaling in cardiomyocytes_Homo sapiens_hsa04261	3.09E-03
TNF signaling pathway_Homo sapiens_hsa04668	4.13E-03
Glutathione metabolism_Homo sapiens_hsa00480	4.05E-03
Hepatitis C_Homo sapiens_hsa05160	4.82E-03
Cell cycle_Homo sapiens_hsa04110	5.41E-03
Small cell lung cancer_Homo sapiens_hsa05222	7.03E-03

IO-1

GO Term	*P-val.*
mitotic cell cycle (GO:0000278)	2.95E-06
Ras protein signal transduction (GO:0007265)	4.56E-06
**KEGG pathway**	***P-val.***
Cysteine and methionine metabolism_Homo sapiens_hsa00270	9.34E-04
Small cell lung cancer_Homo sapiens_hsa05222	1.00E-03

IO-2

GO Term	*P-val.*
mitotic cell cycle (GO:0000278)	6.41E-09
Ras protein signal transduction (GO:0007265)	4.44E-07
**KEGG pathway**	***P-val.***
Small cell lung cancer_Homo sapiens_hsa05222	8.99E-05

IO-3

GO Term	*P-val.*
mitotic cell cycle (GO:0000278)	6.99E-09
Ras protein signal transduction (GO:0007265)	1.50E-06
negative regulation of cell cycle (GO:0045786)	1.24E-05
**KEGG pathway**	***P-val.***
Small cell lung cancer_Homo sapiens_hsa05222	6.49E-06
Pancreatic cancer_Homo sapiens_hsa05212	1.77E-05

IO-5

GO Term	*P-val.*
mitotic cell cycle (GO:0000278)	1.58E-09
negative regulation of cell cycle (GO:0045786)	1.60E-06
response to ionizing radiation (GO:0010212)	2.89E-06
Ras protein signal transduction (GO:0007265)	3.43E-06
response to radiation (GO:0009314)	1.19E-05
transforming growth factor beta receptor signaling pathway (GO:0007179)	7.50E-06
**KEGG pathway**	***P-val.***
p53 signaling pathway_Homo sapiens_hsa04115	1.63E-05
Cell cycle_Homo sapiens_hsa04110	1.36E-05
FoxO signaling pathway_Homo sapiens_hsa04068	4.27E-05
Pancreatic cancer_Homo sapiens_hsa05212	7.69E-05
Ubiquitin mediated proteolysis_Homo sapiens_hsa04120	3.03E-05
Small cell lung cancer_Homo sapiens_hsa05222	9.55E-05

P2

GO Term	*P-val.*
mitotic cell cycle (GO:0000278)	7.57E-11
**KEGG pathway**	***P-val.***
Ubiquitin mediated proteolysis_Homo sapiens_hsa04120	2.74E-06
Neurotrophin signaling pathway_Homo sapiens_hsa04722	9.22E-05
Small cell lung cancer_Homo sapiens_hsa05222	1.38E-04

Down-regulated

IO-0.5

GO Term	*P-val.*
membrane lipid catabolic process (GO:0046466)	1.26E-04
glycolipid catabolic process (GO:0019377)	1.93E-04
**KEGG pathway**	***P-val.***
Other glycan degradation_Homo sapiens_hsa00511	2.89E-04
Glutamatergic synapse_Homo sapiens_hsa04724	4.53E-03
Glycine, serine and threonine metabolism_Homo sapiens_hsa00260	4.17E-03
Protein digestion and absorption_Homo sapiens_hsa04974	3.97E-03
Metabolic pathways_Homo sapiens_hsa01100	8.26E-03

IO-1

GO Term	*P-val.*
hexose biosynthetic process (GO:0019319)	3.69E-05
regulation of uterine smooth muscle contraction (GO:0070472)	1.48E-04
monosaccharide biosynthetic process (GO:0046364)	9.99E-05
regulation of blood circulation (GO:1903522)	2.31E-04
response to isoquinoline alkaloid (GO:0014072)	4.58E-04
membrane lipid catabolic process (GO:0046466)	1.09E-03
**KEGG pathway**	***P-val.***
Glycine, serine and threonine metabolism_Homo sapiens_hsa00260	1.82E-03
Glutamatergic synapse_Homo sapiens_hsa04724	3.51E-03
Other glycan degradation_Homo sapiens_hsa00511	2.07E-03
Protein digestion and absorption_Homo sapiens_hsa04974	2.7E-03
Metabolic pathways_Homo sapiens_hsa01100	6.91E-03

IO-2

GO Term	*P-val.*
cellular amino acid metabolic process (GO:0006520)	3.22E-05
membrane lipid catabolic process (GO:0046466)	1.06E-03
glycolipid catabolic process (GO:0019377)	1.05E-03
**KEGG pathway**	***P-val.***
Metabolic pathways_Homo sapiens_hsa01100	6.97E-03
Sphingolipid metabolism_Homo sapiens_hsa00600	9.62E-03
Other glycan degradation_Homo sapiens_hsa00511	1.97E-03

IO-3

GO Term	*P-val.*
pyrimidine nucleobase metabolic process (GO:0006206)	1.52E-03
cellular amino acid metabolic process (GO:0006520)	7.45E-04
cellular component disassembly (GO:0022411)	4.65E-04
T cell proliferation (GO:0042098)	8.61E-04
protein targeting (GO:0006605)	1.48E-03
single-organism carbohydrate catabolic process (GO:0044724)	1.15E-03
hexose biosynthetic process (GO:0019319)	1.34E-03
cellular amino acid biosynthetic process (GO:0008652)	5.55E-04
glial cell differentiation (GO:0010001)	9.61E-04
mononuclear cell proliferation (GO:0032943)	1.11E-03
lymphocyte proliferation (GO:0046651)	8.72E-04
membrane lipid catabolic process (GO:0046466)	2.58E-03
**KEGG pathway**	***P-val.***
Metabolic pathways_Homo sapiens_hsa01100	1.22E-04
Amino sugar and nucleotide sugar metabolism_Homo sapiens_hsa00520	4.85E-03
Other glycan degradation_Homo sapiens_hsa00511	4.36E-03

IO-5

GO Term	*P-val.*
cellular component disassembly (GO:0022411)	7.94E-05
hexose biosynthetic process (GO:0019319)	1.39E-04
protein targeting (GO:0006605)	2.52E-04
pyrimidine nucleobase metabolic process (GO:0006206)	6.05E-04
monosaccharide biosynthetic process (GO:0046364)	3.75E-04
single-organism carbohydrate catabolic process (GO:0044724)	5.41E-04
membrane lipid catabolic process (GO:0046466)	1.2E-03
**KEGG pathway**	***P-val.***
Metabolic pathways_Homo sapiens_hsa01100	9.19E-06
Ribosome_Homo sapiens_hsa03010	6.52E-04
Glycine, serine and threonine metabolism_Homo sapiens_hsa00260	1.5E-03
Other glycan degradation_Homo sapiens_hsa00511	2.26E-03

P2

GO Term	*P-val.*
glycolipid catabolic process (GO:0019377)	2.88E-04
membrane lipid catabolic process (GO:0046466)	4.07E-04
**KEGG pathway**	***P-val.***
Metabolic pathways_Homo sapiens_hsa01100	2.0E-04
Glycine, serine and threonine metabolism_Homo sapiens_hsa00260	2.12E-03
Glycerophospholipid metabolism_Homo sapiens_hsa00564	1.84E-03
Protein digestion and absorption_Homo sapiens_hsa04974	2.4E-03
Other glycan degradation_Homo sapiens_hsa00511	2.89E-03

B

P2 vs

Up-regulated

	GO term	P-val.
ICM	mitotic cell cycle	1.98E-10
IO-0.5	regulation of protein homooligomerization	3.41E-05
IO-1	renal tubule morphogenesis	5.42E-04
	regulation of coenzyme metabolic process	4.35E-04
IO-2	positive regulation of acute inflammatory response	9.14E-04
	intrinsic apoptotic signaling pathway in response to endoplasmic reticulum stress	8.49E-04
IO-3	intrinsic apoptotic signaling pathway in response to endoplasmic reticulum stress	2.12E-04
	regulation of gene expression, epigenetic	1.87E-04

Down-regulated

	GO term	P-val.
ICM	cellular amino acid metabolic process	5.41E-05
IO-0.5	lipid biosynthetic process	2.01E-06
	phospholipid metabolic process	2.51E-06
IO-1	organic acid transport	1.13E-06
	carboxylic acid transport	9.94E-07
	extracellular matrix organization	3.21E-06
IO-2	epithelial cell differentiation	2.18E-06
	organic acid transport	2.27E-06
	carboxylic acid transport	2.06E-06
	smooth muscle cell differentiation	3.19E-05
IO-3	epithelial cell differentiation	6.75E-08
	smooth muscle cell differentiation	3E-05
	definitive hemopoiesis	3.58E-05
	organ formation	6.83E-05
IO-5	organ formation	4.99E-06
	epithelial cell differentiation	1.27E-06
	smooth muscle cell differentiation	3.25E-05
	Angiogenesis	8.62E-06
	regulation of epithelial cell migration	1.05E-05

C

P4 vs

Up-regulated

	GO term	P-val.
ICM	mitotic cell cycle	3.05E-13
	negative regulation of cell cycle	4.01E-08
	cell cycle phase transition	1.47E-08
	mitotic cell cycle phase transition	4.24E-08
IO-0.5	regulation of response to DNA damage stimulus	6.78E-08
	regulation of cellular response to stress	4.42E-07
	DNA repair	3.76E-06
	regulation of DNA metabolic process	4.18E-06
IO-1	intraciliary transport	2.96E-04
	positive regulation of histone methylation	1.89E-03
	regulation of cellular response to stress	3.96E-04
	negative regulation of cell cycle	2.02E-03
IO-2	endosome organization	1.74E-03
IO-3	male gonad development	1.85E-05
	reproductive structure development	6.82E-05
	gonad development	7.68E-05

Down-regulated

	GO term	P-val.
ICM	cellular amino acid metabolic process	2.62E-05
	membrane lipid catabolic process	3.5E-04
IO-0.5	mannosylation	2.1E-04
	glutathione derivative metabolic process	6.99E-05
IO-1	mannosylation	7.11E-06
	endocytosis	1.6E-05
	glutathione derivative metabolic process	7.68E-05
	glutathione derivative biosynthetic process	7.68E-05
	post-translational protein modification	1.89E-05
	Glycosylation	6.55E-05
IO-2	extracellular matrix organization	5.42E-06
	extracellular structure organization	5.69E-06
	protein N-linked glycosylation via asparagine	2.12E-05
	peptidyl-asparagine modification	2.34E-05
	protein N-linked glycosylation	4.52E-05
IO-3	extracellular matrix organization	1.72E-08
	extracellular structure organization	1.79E-08
	regulation of epithelial cell migration	2.1E-06
	epithelial cell differentiation	6.59E-06
	smooth muscle cell differentiation	2.65E-05
	tissue morphogenesis	9.43E-06
IO-5	extracellular matrix organization	1.5E-07
	extracellular structure organization	1.54E-07
	response to decreased oxygen levels	2.96E-06
	response to hypoxia	2.6E-06
	response to oxygen levels	4.59E-06
	trabecula formation	8.71E-06
	epithelial cell differentiation	2.03E-06
	regulation of epithelial cell migration	3.55E-06

D

P15 vs

Up-regulated

	GO term	P-val.
ICM	mitotic cell cycle	4.99E-11
	negative regulation of cell cycle	3.44E-07
	organelle fission	1.96E-07
	nuclear division	1.2E-07
	proteolysis involved in cellular protein catabolic process	7.58E-07
	modification-dependent macromolecule catabolic process	8.07E-07
	modification-dependent protein catabolic process	9.66E-07
	cell cycle phase transition	7.01E-07
IO-0.5	regulation of cellular response to stress	1.12E-06
	DNA repair	1.03E-06
	regulation of DNA metabolic process	6.44E-07
	mitotic cell cycle	5.78E-07
	chromatin modification	4.07E-06
	regulation of response to DNA damage stimulus	4.57E-06
	organelle fission	1.32E-05
	nuclear division	1.18E-05
	cell cycle phase transition	2.12E-05
IO-1	DNA repair	1.27E-07
	mitotic cell cycle	2.86E-07
	organelle fission	1.00E-06
	nuclear division	2.35E-06
	chromatin modification	4.74E-06
	cell cycle checkpoint	5.84E-06
	regulation of DNA metabolic process	1.53E-05
IO-2	organelle fission	5.22E-11
	nuclear division	8.28E-11
	mitotic cell cycle	4.62E-10
	mitotic nuclear division	3.31E-09
	chromatin modification	8.76E-07
	histone modification	5.8E-06
	covalent chromatin modification	7.15E-06
IO-3	positive regulation of macroautophagy	4.17E-04
	proteolysis involved in cellular protein catabolic process	2.74E-04
	modification-dependent macromolecule catabolic process	1.25E-04
	modification-dependent protein catabolic process	2.9E-04
	ubiquitin-dependent protein catabolic process	5.55E-04
	organelle fission	5.29E-04
	nuclear division	5.49E-04
	mitotic cell cycle	4.15E-04
IO-5	organelle fission	5.7E-04
	nuclear division	3.09E-04

Dow-regulated

	GO term	P-val.
ICM	translation	2.77E-08
	gene expression	4.57E-06
	protein localization to membrane	3.18E-06
	negative regulation of protein modification process	1.51E-05
	single-organism cellular localization	1.35E-05
	cellular component disassembly	2.12E-05
	protein targeting	2.75E-05
IO-0.5	translation	4.37E-14
	gene expression	4.64E-10
	translational initiation	4.70E-07
	electron transport chain	2.86E-07
	respiratory electron transport chain	5.13E-07
	protein targeting to ER	1.25E-06
	protein localization to endoplasmic reticulum	1.27E-06
IO-1	translation	2.25E-12
	gene expression	8.34E-10
	protein localization to endoplasmic reticulum	8.17E-07
	protein localization to membrane	2.33E-05
	translational initiation	1.24E-05
	protein targeting to ER	6.89E-06
	protein N-linked glycosylation via asparagine	1.92E-05
IO-2	translation	1.27E-08
	gene expression	3.69E-08
	negative regulation of transferase activity	9.38E-05
	protein N-linked glycosylation via asparagine	6.57E-05
	peptidyl-asparagine modification	7.44E-05
IO-3	gene expression	1.86E-05
	establishment or maintenance of epithelial cell apical/basal polarity	5.96E-04
	smooth muscle cell differentiation	8.39E-04
	epithelial cell differentiation	7.46E-05
IO-5	regulation of epithelial cell migration	3.63E-08
	smooth muscle cell differentiation	1.29E-05
	muscle cell differentiation	2.71E-05
	organ formation	2.53E-04
	positive regulation of locomotion	1.11E-04
	response to hypoxia	1.38E-04
	response to decreased oxygen levels	1.62E-04
	positive regulation of cell migration	1.64E-04
	tissue morphogenesis	6.06E-05
	positive regulation of epithelial cell migration	5.2E-05

E

P4 vs P2

Up-regulated

GO term	P-val.
endosome organization	2.01E-03

Dow-regulated

GO term	P-val.
regulation of translational elongation	8.15E-04

P15 vs P4

Up-regulated

GO term	P-val.
chromosome organization involved in meiosis	5.257E-05

Dow-regulated

GO term	P-val.
lysosome localization	4.67E-04

Figure S2. Functional annotation for up- and down-regulated genes during the transition from ICM cells to ESCs, related to Figure 2.

(A) Functional annotation for DEG in the ICM versus different IOs/ESCs. The pathways significantly enriched by the GO term and KEGG pathways are shown. The analysis has been performed by the Enrichr web tool.

(B-D) Functional annotation for DEG in ESCs of different passages (P) versus the ICM. Tables show the statistically significant enriched pathways for ESCs of P2 **(B)**, P4 **(C)**, and P15 **(D)** versus ICM/different IOs.

(E) Functional annotation of DEG between ESCs of different passages.

A

Heatmap I

GO Term	P-val.
regulation of fat cell differentiation (GO:0045598)	1.9E-04
glutathione metabolic process (GO:0006749)	2.46E-04
skeletal muscle cell differentiation (GO:0035914)	2.12E-04
**KEGG pathway**	**P-val.**
Glutathione metabolism_Homo sapiens_hsa00480	5.5E-04
**Wikipathway**	**P-val.**
Glutathione metabolism_Homo sapiens_WP100	5.5E-04

Heatmap II

GO Term	P-val.
anion transport (GO:0006820)	1.35E-04
**Wikipathway**	**P-val.**
Preimplantation Embryo_Homo sapiens_WP3527	3.52E-03

Heatmap III

GO Term	P-val.
chromatin modification (GO:0016568)	7.49E-06
DNA repair (GO:0006281)	2.49E-05
DNA catabolic process (GO:0006308)	1.92E-05
regulation of DNA metabolic process (GO:0051052)	1.38E-04
DNA-templated transcription, initiation (GO:0006352)	8.91E-05
mitotic cell cycle (GO:0000278)	1.41E-04
transcription initiation from RNA polymerase II promoter (GO:0006367)	1.03E-04
chromatin remodeling (GO:0006338)	8.18E-05
**KEGG pathway**	**P-val.**
Ribosome biogenesis in eukaryotes_Homo sapiens_hsa03008	1.44E-03
Melanoma_Homo sapiens_hsa05218	4.19E-03
RNA transport_Homo sapiens_hsa03013	7.72E-03
**Wikipathway**	**P-val.**
PluriNetWork_Mus musculus_WP1763	8.23E-06

Heatmap V

GO Term	P-val.
regulation of cofactor metabolic process (GO:0051193)	1.22E-04
**KEGG pathway**	**P-val.**
Signaling pathways regulating pluripotency of stem cells_Homo sapiens_hsa04550	2.08E-05
**Wikipathway**	**P-val.**
PluriNetWork_Mus musculus_WP1763	8.80E-04

Heatmap V

GO Term	P-val.
endoderm formation (GO:0001706)	2.18E-05
**Wikipathway**	**P-val.**
Endoderm Differentiation_Homo sapiens_WP2853	1.4E-04

Heatmap VI

GO Term	P-val.
lipid homeostasis (GO:0055088)	1.01E-05
plasma lipoprotein particle assembly (GO:0034377)	2.81E-04
sterol homeostasis (GO:0055092)	9.7E-05
protein destabilization (GO:0031648)	4.63E-04
cholesterol homeostasis (GO:0042632)	9.26E-05
**KEGG pathway**	**P-val.**
Rap1 signaling pathway_Homo sapiens_hsa04015	7.91E-03
**Wikipathway**	**P-val.**
Cardiac Progenitor Differentiation_Homo sapiens_WP2406	1.22E-03

Heatmap VII

GO Term	P-val.
response to topologically incorrect protein (GO:0035966)	3.69E-07
integrin-mediated signaling pathway (GO:0007229)	6.26E-07
response to unfolded protein (GO:0006986)	8.46E-07
regulation of cell morphogenesis (GO:0022604)	2.33E-05
cellular response to topologically incorrect protein (GO:0035967)	8.35E-06
angiogenesis (GO:0001525)	1.6E-05
ER-nucleus signaling pathway (GO:0006984)	1.02E-05
endoplasmic reticulum unfolded protein response (GO:0030968)	1.73E-05
cellular response to unfolded protein (GO:0034620)	2.34E-05
activation of signaling protein activity involved in unfolded protein response (GO:0006987)	6.69E-05
vasculogenesis (GO:0001570)	9.21E-05
positive regulation of nuclease activity (GO:0032075)	8.29E-05
**KEGG pathway**	**P-val.**
Lysosome_Homo sapiens_hsa04142	1.69E-07
Amino sugar and nucleotide sugar metabolism_Homo sapiens_hsa00520	4.17E-05
**Wikipathway**	**P-val.**
Fatty acid oxidation_Mus musculus_WP2318	4.1E-04
Glutathione metabolism_Mus musculus_WP164	2.11E-03
TGF-beta Signaling Pathway_Homo sapiens_WP366	1.54E-03

Heatmap VIII

GO Term	P-val.
hepatocyte apoptotic process (GO:0097284)	2.01E-04
calcium-independent cell-cell adhesion via plasma membrane cell-adhesion molecules (GO:0016338)	9.95E-04
**KEGG pathway**	**P-val.**
Alzheimer's disease_Homo sapiens_hsa05010	8.82E-03

Heatmaps I-V

GO terms	P-val.
negative regulation of cellular component organization (GO:0051129)	5.3E-04
regulation of DNA metabolic process (GO:0051052)	4.84E-04
chromatin modification (GO:0016568)	2.84E-04
pattern specification process (GO:0007389)	5.21E-04
regulation of collateral sprouting (GO:0048670)	6.85E-04
negative regulation of cell development (GO:0010721)	6.72E-04
negative regulation of cell-substrate adhesion (GO:0010812)	6.88E-04

KEGG pathway	P-val.
Signaling pathways regulating pluripotency of stem cells_Homo sapiens_hsa04550	5.68E-04
B cell receptor signaling pathway_Homo sapiens_hsa04662	5.33E-03
Melanoma_Homo sapiens_hsa05218	4.52E-03
Ribosome biogenesis in eukaryotes_Homo sapiens_hsa03008	6.13E-03
Dorso-ventral axis formation_Homo sapiens_hsa04320	8.3E-03

Wikipathway	P-val.
PluriNetWork_Mus musculus_WP1763	1.03E-09
Interactome of polycomb repressive complex 2 (PRC2) _Homo sapiens_WP2916	5.71E-04
Preimplantation Embryo_Homo sapiens_WP3527	2.12E-03

Heatmaps V-VIII

GO terms	P-val.
angiogenesis (GO:0001525)	1.53E-05
cell morphogenesis (GO:0000902)	1.91E-05
integrin-mediated signaling pathway (GO:0007229)	7.93E-06
response to topologically incorrect protein (GO:0035966)	8.42E-06
response to unfolded protein (GO:0006986)	1.6E-05
vasculogenesis (GO:0001570)	2.42E-05

KEGG pathway	P-val.
Lysosome_Homo sapiens_hsa04142	1.04E-06
Amino sugar and nucleotide sugar metabolism_Homo sapiens_hsa00520	2.19E-04
Protein processing in endoplasmic reticulum_Homo sapiens_hsa04141	1.35E-03

Wikipathway	P-val.
Focal Adhesion_Homo sapiens_WP306	1.97E-03
Focal Adhesion_Mus musculus_WP85	1.45E-03
Primary Focal Segmental Glomerulosclerosis FSGS_Homo sapiens_WP2572	1.75E-03
Fatty acid oxidation_Mus musculus_WP2318	1.04E-03

B

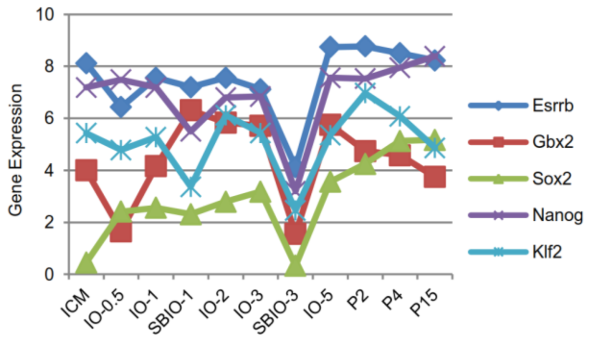

Figure S3. Transcriptome signature of R2i-treated IOs, related to Figure 3.

(A) Functional annotation for the represented heatmaps in Figure 3. Heatmaps I-IV show the up-regulated genes in R2i versus SB in IO-1 and IO-3, and we refer to these genes as R2i specific. Heatmaps V-VIII represent the down-regulated genes in R2i versus SB on days 1 and 3, and we consider these genes as SB specific.

(B) The expression patterns of 5 of 12 essential transcription factors for naïve pluripotency showed significant differences between IO-3 and SBIO-3. The expression of the other genes was not significantly different between R2i- and SB-treated IOs.

A

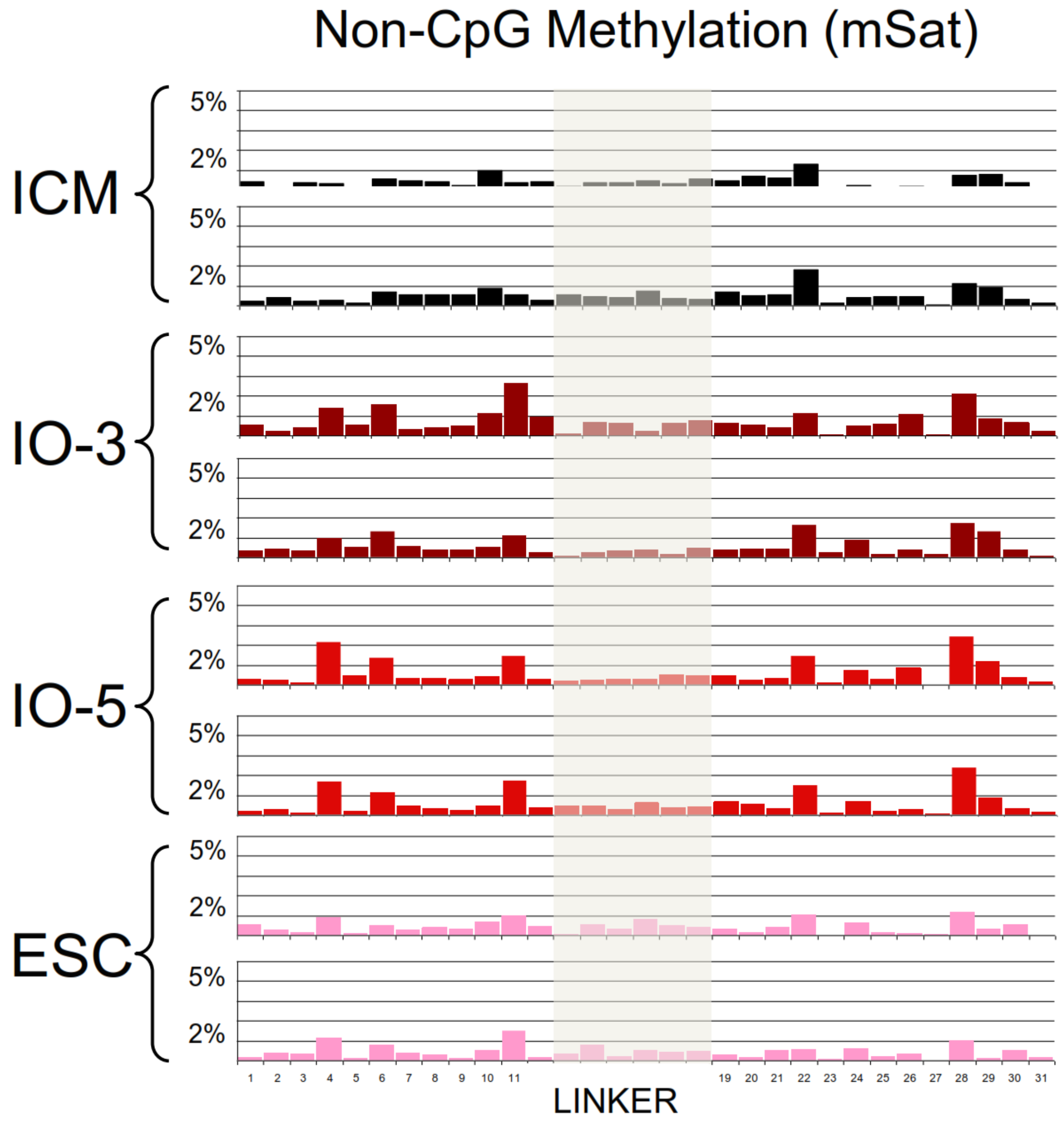

Figure S4. DNA methylation changes during the ICM to ESC transition, related to Figure 5.

Hairpin-bisulfite amplicon sequencing of non-CpG methylation of mSat. The bars sum up the DNA methylation status of all non-CpG positions.

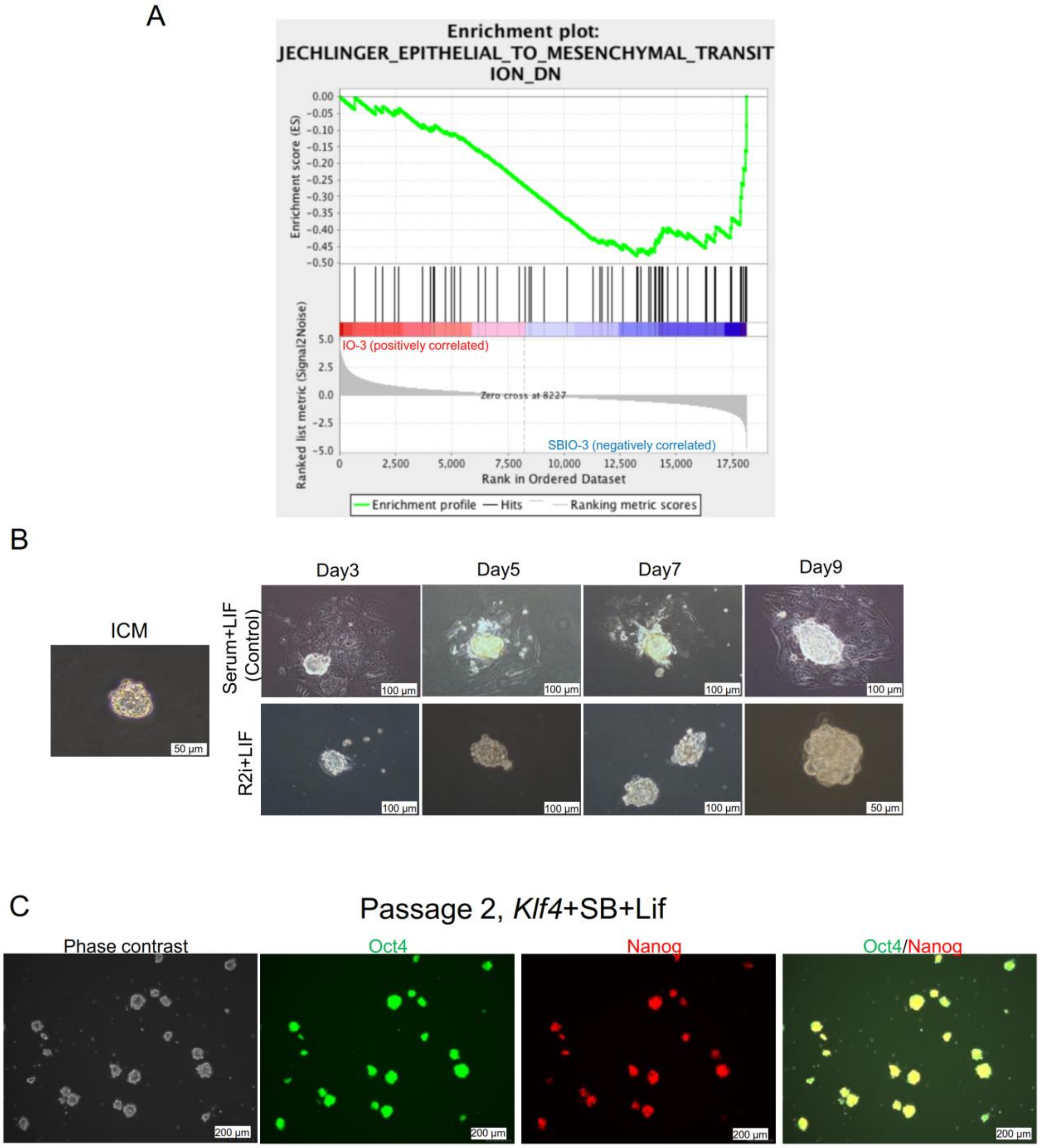
A
Enrichment plot:
JECHLINGER_EPITHELIAL_TO_MESENCHYMAL_TRANSITION_DN
Enrichment score (ES)
IO-3 (positively correlated)
Zero cross at 8227
SBIO-3 (negatively correlated)
Ranked list metric (Signal2Noise)
Rank in Ordered Dataset
Enrichment profile
Hits
Ranking metric scores
B
ICM
Day3
Day5
Day7
Day9
Serum+LIF (Control)
R2i+LIF
50 μm
100 μm
C
Passage 2, Klf4+SB+Lif
Phase contrast
Oct4
Nanog
Oct4/Nanog
200 μm

Figure S5. EMT blockage is required for the establishment of ESCs, related to Figure 6.

(A) GSEA indicated that SB-related genes were enriched in the EMT biological process.

(B) Morphological comparison between serum/LIF- and R2i-treated IOs. Note the expanded cell migration around the serum/LIF-treated IOs.

(C) Phenotype of established ESCs after *Klf4* induction in SB-treated IOs.

A

P15 vs E4.5 preEpi

Up-regulated

GO Term	P-val.
male meiosis I (GO:0007141)	5.05E-03
**KEGG pathway**	**P-val.**
Vasopressin-regulated water reabsorption_Homo sapiens_hsa04962	0.01
Proteoglycans in cancer_Homo sapiens_hsa05205	0.01
FoxO signaling pathway_Homo sapiens_hsa04068	0.01
p53 signaling pathway_Homo sapiens_hsa04115	9.97E-03
**Wikipathway**	**P-val.**
TOR Signaling_Homo sapiens_WP1471	4.94E-03
Alzheimers Disease_Mus musculus_WP2075	0.01
p53 signaling_Mus musculus_WP2902	6.83E-03

Dow-regulated

GO Term	P-val.
translation (GO:0006412)	2.69E-20
gene expression (GO:0010467)	2.78E-15
**KEGG pathway**	**P-val.**
Ribosome_Homo sapiens_hsa03010	4.6E-10
Oxidative phosphorylation_Homo sapiens_hsa00190	7.13E-07
**Wikipathway**	**P-val.**
Electron Transport Chain_Mus musculus_WP295	3.68E-07

B

Heatmaps		E3.5 ICM	E3.5 ICM/E4.5 EPI	E4.5 EPI	E4.5 EPI/E5.5 EPI	E5.5 EPI	Diapaused
Non-Sig		204	97	67	113	123	17
R2i	I	11	3	0	0	2	1
R2i	II	1	3	0	1	0	4
R2i	III	9	18	21	26	29	6
R2i	IV	2	0	1	3	1	1
SB	V	2	3	3	1	2	0
SB	VI	3	0	2	0	0	0
SB	VII	43	15	3	5	7	0
SB	VIII	3	0	1	0	3	0
Total		278	139	98	149	167	29
Boroviak (2015)		335	169	135	190	207	30

D

Clusters		E3.5 EPI	E3.5-E4.5 EPI	E4.5 EPI	E3.5 PE	E3.5-E4.5 PE	E4.5 PE
0		6	3	133	10	10	174
R2i	I	2	1	2	0	0	1
R2i	II	1	2	2	0	0	1
R2i	III	3	1	56	1	0	2
R2i	IV	0	0	2	0	0	0
SB	V	0	0	3	1	3	10
SB	VI	0	0	0	1	1	10
SB	VII	0	0	2	0	3	56
SB	VIII	0	0	2	0	0	3
Total		12	7	202	13	17	257
Gerovska (2016)		16	8	278	17	19	359

C

	ICM	ICM/Pre Epi 4.5	Pre Epi 4.5	Pre Epi/Post Epi	Post Epi	Diapaused
I	Eomes,Gsc,2610528J11Rik,Sri,Lgals4,Gm2a,Psap,AA467197,Serpinb6c	Zfhx2,Ostf1,Gpx1			Fxyd6,Cmtm8	Tcfap2c
II	BC053393	Jam2,Nanog,Klf2		Wfdc2		Klf2,Nanog,Dppa3,Msc
III	Upp1,Uhrf1,Serpina3m,Tuba4a,Ly6g6e,Mgmt,Fzd5,Arhgef19,Rfx2	Itgb7,Spp1,Ephx2,Tdgf1,Socs2,Ly6e,Aoah,Gdf3,Syt9,Sh3gl2,Oas1g,Vegfc,Zfp819,Tcl1,Ddx58,Stat4,Mybl2	Fez1,8430410A17Rik,Trh,Laptm5,Zfp428,Gsta4,Dnmt3l,Gpc4,Fstl1,Lrrc34,Tubb2b,Enox1,2610305D13Rik,Smtnl2,Pdcl,Hibadh,Dnd1,Esrrb,D14Ertd668e,Csrnp2,Cdc42ep3	Utf1,Sox2,Slc7a3,Zscan10,L1td1,Eml4,Nup210,Etv5,Sec14l1,Prdx1,Vim,Tmem40,Stmn2,Ifitm1,Pla2g1b,Bhlhb9,Ifitm3,Dek,Tpd52l1,Ak1,Ifitm2,Pipox,Zfp532,Psme4,Thy1,Tcea3	Igfbp2,Irf1,Hmga1,Cbr3,Pdk3,Rab34,Crabp2,Ina,Cd81,Pim2,Col18a1,Dab1,Fgf5,Phc1,Myb,Acot1,Sort1,Dtx1,Elmo1,Pou3f1,Klk8,Dnmt3b,Sall2,Enpp2,Cd276,Armcx2,Plekha1,2810417H13Rik,Otx2	Utf1,Sox2,Gdf3,Tdgf1,Esrrb,Tbx3
IV	Gna14,Axin2		Robo4	Zic3,Aire,Armcx1	Fzd7	Gbx2
V	Pnliprp2,Scmh1	Egr1,Dkk1,Col4a1	Zfp36l1,Htra1,Etv4	6330407J23Rik	Podxl,Emb	
VI	Aqp8,Pdgfra,Hnf4a				H19	
VII	Fhod1,Junb,Gpx2,Cpt2,Fgf10,Zyx,Zdhhc12,Rhou,Egfl7,Gss,Sgpl1,Acaa2,Polg,Serpinb6a,Ly6a,Nuak1,Ralb,Fbxo2,Amdhd2,Pvrl2,Ppap2c,Erp29,Elovl1,Cap1,Grina,Txndc12,Hexa,Gas2l1,Xist,Commd3,Adam15,Mfsd1,Gabarapl1,Tcf23,Myd88,Ap1m2,Stat3,Pdia5,Gdpd5,Plekhf1,Fbxo6,Tmbim1,Inadl	Serpinh1,Plod2,Mfge8,Rhox5,Rhoc,Col4a2,Apoc1,Retsat,Ext2,Pde1b,Srgn,Amn,Pga5,Ctgf,Cst3	Wdr1,S100a6,Ddah1	Obsl1,Bex2,Itm2b,Amfr,Txndc5	Car4,Cul7,Pxdn,Dusp9,Efs,2310021P13Rik	
VIII	Cldn3,Arpc1b,Wnt7b		Ndufs4		Tmem54,Tpm2,Lzts2	

E

	E3.5 EPI	E3.5 & E4.5 EPI	E4.5 EPI	E3.5 PE	E3.5 & E4.5 PE	E4.5 PE
I	Tcfap2c,Spic	Cldn4	Lgals3,AU018091			Serpinb6c
II	Slc1a1	Nanog,Klf2	Wfdc2,Pla2g10			Pdzk1
III	Sgk1,Ubxn2a,Liph	Zp3	Hmga1,Gsta4,Sox2,Slc7a3,Angptl4,Pim2,Smpdl3b,Rfx2,Sap30,Cdyl,Col18a1,Mkrn1,Socs2,Sirt1,Trh,Gtsf1l,Vegfc,Nptx2,Phc1,Stmn2,8430410A17Rik,Gprasp1,Zfp428,Tcea3,Mtss1,Tmem40,Otx2,Plekha4,Cth,Rcc2,Klhl7,Etv5,Dppa4,Capsl,Lrp11,Fntb,Nkrf,A830080D01Rik,Zfp532,Pcyt1b,Ddx58,Enox1,Esrrb,Ankrd6,Ulk1,Mbtd1,Gdf3,Bcat1,Tdgf1,Notum,Skil,Igfbp2,Mybl2,Trim37,Mical1,Btbd11	Dnmt3l		Sgk3,Pls3
IV			Spred1,Axin2			
V			Etv4,6330407J23Rik,Bcl2l11	Dusp4	Cpn1,Dkk1,Col4a1	Emb,Htra1,Tcfec,Rcn3,Gata6,Col4a1,Dnajc10,AA986860,Bmp6,P4ha1
VI				Tfpi	Pdgfra	Spink3,Aqp8,Ctsh,Pla2g12b,Ctdspl,Hnf4a,Pdgfra,Klb,Cobll1,Soat2
VII			Acaa2,Fbp2		P4ha2,Sox17,Serpinh1	Txndc12,Plod3,Cotl1,Tmed3,Cryab,Serping1,Plod2,Neu1,Tmem218,Zyx,Srgn,Atp6v0a1,Ctsz,Amn,Galnt10,Kdelr3,Pcbd1,Timd2,Rhox6,Mttp,Gaa,Nus1,Dab2,Pga5,Sox17,Polg,Hs3st1,Txndc5,Col4a2,Gdpd5,BC017158,Elovl1,Tmem144,Lrpap1,Galnt2,Agpat4,0610007C21Rik,2310021P13Rik,Gpr137b,Commd3,Dusp9,Myo6,Slc9a6,Ralb,Leprel1,Grina,Uap1l1,Xist,Nostrin,Lgmn,Cited1,Pdzd3,Gba,Myo5b,Coq2,Fut8
VIII			Ldoc1,Lasp1			Plac1,Krt8,Cldn7

Figure S6. R2i-treated IO indicated preEpi cell characteristics, related to Figure 7.

(A) Functional annotation for up- and down-regulated genes in ESCs of P15 versus E4.5 preEpi cells (Boroviak et al.).

(B) A comparison of gene expression shown in R2i- and SB-specific heatmaps (in Figure 3A) and those identified by Boroviak et al., which were associated with different lineages of the early mouse embryo.

(C) List of genes related to (B).

(D). A comparison of gene expression shown in R2i- and SB-specific heatmaps (in Figure 3A) and those genes identified by Gerovska and Arauzo-Bravo as associated with the epiblast (EPI) and primitive endoderm (PE).

(E) List of genes related to (D).

Supplemental Experimental Procedures

Mouse strains, E3.5 blastocyst collection

All mice were maintained on a 12-hour light/dark schedule. We used the following mice and embryo strains in this study: BALB/c (for the first run of the microarray and DNA methylation sequencing), NMRI (For RG108 treatment and R2i time-dependency experiment), F1 hybrids (B6 × C3H), (C57BL/6 × C3H) F1 female × CD1 male mice, and OG2 × F1 hybrids (B6 × C3H) (for the second run of the microarray and EMT/MET induction). All E3.5 blastocysts were collected by flushing the uterus after superovulation according to a previously described protocol (Hassani et al., 2012).

OG2 are transgenic mice in which GFP is expressed under the control of a Pou5F1 promoter and distal enhancer. This GFP reporter is expressed in the ICM of blastocysts on E3.5, the epiblast on E4.5, and in ESCs (Yeom et al., 1996). We used the Tet-On Nanog inducible blastocyst from F1 (C57BL/6 × C3H) × OG2 (Fischedick et al., 2014).

All procedures that used animals were in strict accordance with the approval of the Royan Institutional Review Board and Institutional Ethical Committee.

Culture medium

Serum-free N2B27 supplemented medium was the base medium for all experiments except where otherwise indicated. N2B27 supplemented medium consisted of DMEM/F12 (Invitrogen) and neurobasal (Invitrogen) in a 1:1 ratio, 1% N2 supplement (Invitrogen), 1% B27 supplement (Invitrogen), 1% nonessential amino acids (Invitrogen), 2 mM L-glutamine (Invitrogen), 100 U/ml penicillin and 100 mg/ml streptomycin (Invitrogen), 0.1 mM β-mercaptoethanol (Sigma-Aldrich), and 5 mg/mL BSA (Sigma-Aldrich). Mouse ESC (serum) medium consisted of

knockout Dulbecco's modified Eagle's medium (Invitrogen), 15% fetal bovine serum (FBS, HyClone), 1% nonessential amino acids, 2 mM L-glutamine, 100 U/ml penicillin, 100 mg/ml streptomycin, 0.1 mM β-mercaptoethanol, and 1000 U/ml mouse LIF.

Derivation of ESCs and sample collection

For isolation of the ICM, the zona of E3.5 blastocysts was removed using acidic Tyrode's solution (pH=2.2), and blastocysts were incubated with mouse trophoblast antibody for 40 min. Next, blastocysts were treated with guinea pig complement in 50 µl droplets under oil for 10 min. After bubbling and lysis of the trophectoderm cells, ICM cells were removed by pipetting. The isolated ICMs were washed twice in PBS, and then selected for microarray analysis. The procedure of deriving ESCs from early-stage mouse embryos was described previously in detail (Hassani et al., 2014b). Briefly, zona-free E3.5 blastocysts/isolated ICMs were plated on gelatin-coated plates (0.1%, Sigma-Aldrich) containing N2B27 defined medium supplemented with R2i (consisting of 1 µM PD0325901 [Stemgent] and 10 µM SB431542 [Sigma-Aldrich]) as well as 1000 U/ml LIF (ESGRO, Millipore). The day that E3.5 blastocysts/isolated ICMs were transferred into the ESC culture was designated as day 0. For the first microarray analysis, the samples included isolated ICMs (day 0), blastocyst outgrowths (BOs) on days 3, 5, 7, and 9 (BO3-9) after plating, and ESCs of passage 20, in three biological replicates. We chose and pooled approximately 20 to 30 isolated ICMs and BOs for each biological replicate.

For the second microarray analysis, we collected new samples in a time resolution experiment that included immunosurgically isolated ICMs, IOs on days 0.5, 1, 2, 3, and 5 (IO 0.5-5), and ESCs of passages 2, 4, and 15 (P2-15). In addition, we collected IOs cultivated in N2B27

supplemented with SB431542+LIF on days 1 (SBIO-1) and 3 (SBIO-3) as the negative controls. Approximately 30 to 40 ICMs or IOs were picked and pooled in two biological replicates.

RNA extraction, cDNA pre-amplification, and microarray profiling

Isolated ICMs, IOs, and ESCs were collected and preserved at -80°C until RNA extraction. Total RNA was isolated using the AllPrep DNA/RNA Micro Kit (QIAGEN). Integrity and quality of RNA samples was checked using a RNA 2100 Bioanalyzer (Agilent). RNA samples (RIN > 9) were subjected to a two-round amplification performed using the TargetAmp 2-Round Biotin-aRNA Amplification Kit 3.0 (Epicentre) according to the manufacturer's instructions. Purified and labeled cRNA was used for each hybridization reaction onto BeadChip Array Mouse WG-6 and MouseRef-8 v2.0 (Illumina, San Diego, CA, USA), and scanning was performed using the iScan reader (Illumina, San Diego, CA, USA).

Real-time PCR

Total RNA was isolated using the AllPrep DNA/RNA Micro Kit (QIAGEN). For quantitative RT-PCR, cDNA was generated using QuantiTect Whole Transcriptome Kit (QIAGEN) according to the manufacturer's protocol. All amplified cDNA samples were diluted 1 to 50, and 2 μl of each mixture was used for each qRT-PCR reaction. Gene expression experiments were performed using the SYBR green master mix and 7900HT Sequence Detection System (Life Science) in triplicate and two independent biological replicates. The amount of mRNA was normalized to the *Gapdh* and *Actb* housekeeping genes. Relative quantification of gene expression was calculated using the ΔΔCt method. All the primer sequences are listed as below:

Gene Symbol	Sequence	Size (bp)	Annealing Temperature (°C)	Accession number
Pou5f1(Oct4)	F: 5' gcg ttc tct ttg gaa agg tg 3'<br>R: 5' cgg ttc tca atg cta gtt cg 3'	204	61	NM_013633.2
Nanog	F: 5' ctg att ctt cta cca gtc cca 3'<br>R: 5' aaa cca ggt ctt aac ctg ctt at 3'	235	61	NM_028016.2
Esrrb	F: 5' agg ctc tca ttt ggg cct agc 3'<br>R: 5' atc ctt gcc tgc cac ctg tt 3'	102	61	NM_001159500.1
Tcf3	F: 5' cag cag tga cca gaa cag t 3'<br>R: 5' gaa gcc agc ctg act caa g 3'	195	61	NM_001164147.1
Klf4	F: 5' tgt gtc gga gga aga gga agc 3'<br>R: 5' acg act cac caa gca cca tca 3'	76	61	NM_010637
Cdh1	F: 5' cgg ata acc aga aca aag acc a 3'<br>R: 5' agc agg atc aga atc agc ag 3'	168	61	NM_009864.2
Dab2	F: 5' caa caa agc aga aga gaa tgg aag 3'<br>R:5' act att tag gtc agg agg tgt aga 3'	126	61	NM_023118.5
Eomes	F:5' gct tca aca taa acg gac tca 3'<br>R:5' cat ctt att gcc ctg cat gtt att 3'	145	61	NM_010136
Snail	F:5' tct gaa gat gca cat ccg aa 3'<br>R:5' act ggt atc tct tca cat ccg a 3'	208	61	NM_011427
Dnmt1	F:5' gga tga gag gga gga gaa gag 3'<br>R:5' cag gtt agg gtc gtc tag gt 3'	171	62	NM_001199433
Dnmt3l	F:5' cat cca gca ttac gtc ctc at 3'<br>R:5' tct cca ggt cca agg ttt caa 3'	159	61	NM_019448.4
Dnmt3b	F: 5' gtt tat atg agg gca cag gaa 3'<br>R: 5' gcc aca aca ttc tcg aac at 3'	114	62	NM_001003961
Suz12	F: 5' gcc ttt gag aaa cca aca cag 3'<br>R: 5' cag gac ttc cag ggt aac ag 3'	296	61	NM_199196
Sirt1	F: 5' gca cta att cca agt tct ata ccc 3'<br>R: 5' cac cac cta gcc tat gac ac 3'	141	61	NM_019812
Mat2b	F: 5' ggc aga gca gtt tac aaa gag 3'<br>R: 5' cac tat gac atg agg ctg ga 3'	152	61	NM_001199274
Ezh2	F: 5' tgc ttc cta cat ccc ttc ca 3'<br>R: 5' gtg gtg tct tta tac gct cag 3'	159	61	NM_007971

DNA methylation analysis by pyrosequencing

Genomic DNA from the ICM, ICM outgrowths on days 3 and 5, and ESCs was extracted using an AllPrep DNA/RNA Micro Kit (QIAGEN). The protocol for deep hairpin-bisulfite sequencing (DHBS) was previously described (Arand et al., 2012). Briefly, DNA was digested with an element-specific restriction enzyme followed by ligation of a hairpin oligonucleotide linker for major Satellites (mSat), the 5′ untranslated region of L1Md_Tf (L1), and a class of LTR-

retrotransposons (IAP-LTR1). Then, bisulfite-treated DNA was amplified by an element-specific PCR and sequenced with the standard 454-sequencer. BiQAnalyzerHT (Lutsik et al., 2011) was used to determine the methylation status of the CpG dyads and CNG positions.

Viral vectors

We examined the impact of EMT/MET induction on the derivation of ESCs by transducing isolated ICMs with a lentivirus that encoded mesenchymal or epithelial-related genes. To generate viral particles, we cloned *Snail-*, *Klf4-*, *Cdh1-2a-Tomato*, and *ShCdh1* into a pLVTHM (Han et al., 2011) vector backbone. As the control, we used a vector that encoded *Tomato*. Gene constructs were then transformed into DH5α bacteria, and plasmid DNA was extracted with a HiSpeed Plasmid Maxi Kit (QIAGEN). Next, plasmids were transfected into 293T packaging cells using the Fugene6 transfection reagent. After 6 hours, the medium was replaced by ESC medium. Supernatants were harvested for ICM infection after 24 hours. ICMs were cultured and transfected in the N2B27 base medium supplemented with 1 µM PD0325901 (Stemgent), 10 µM SB431542 (Sigma-Aldrich), and 1000 U/ml LIF (ESGRO®, Millipore) with 1 µg/ml polybrene (Sigma-Aldrich). The ICM cells were washed and replated after 24 hours. During this time, the medium was renewed every other day. Five days after ICM infection, ICM outgrowths were picked and disaggregated by dispase (1 mg/ml, Gibco). The dissociated cells were cultured in R2i. Once ESC colonies emerged, they were expanded for three passages to calculate the efficiency of deriving ESCs.

Immunofluorescence analysis

For immunofluorescence analysis, ICM outgrowths and ESCs were fixed in 4% paraformaldehyde (Sigma-Aldrich) for 20 minutes and then permeabilized with 0.2% Triton X-100 for 30 minutes. The procedure was followed by blocking the cells in 10% goat serum in PBS for 1 hour at room temperature and an incubating them overnight with primary antibodies at 4°C. Next, ICM outgrowths and ESCs were washed and incubated with NANOG secondary antibodies. Counterstaining of the nuclei was performed using 2 µg/ml DAPI (Sigma-Aldrich). A fluorescent microscope (Olympus, Japan) was used to visualize the cells.

Statistical and bioinformatics analysis

RAW expression values were extracted from Illumina GenomeStudio. Data was background corrected and quantile normalized by the “neqc” function of the limma R/Bioconductor package (Ritchie et al., 2015). Differentially expressed genes were identified by the Empirical Bayesian method of the limma package. We used the custom R program for visualization and data analysis. The pairwise correlation heatmaps for samples were generated based on Pearson Correlation Coefficients (PCC) using the R/Bioconductor package pheatmap. Principal Coordinates Analysis (PCA) was performed with the Multidimensional Scaling (MDS) method of package limma, and visualized using the package ggplot2. The numbers of differentially expressed genes between consecutive time points were calculated by 1 and 0.01 as the cutoffs for the absolute log2 fold change and Benjamini-Hochbert adjusted *p*-values, respectively. The same criteria were used for other differential expression analyses between our samples. The Gene Ontology (GO) and pathway analysis of differentially expressed genes were performed using Enrichr (Chen et al., 2013). Represented heatmaps of the genes in Figure 3A were created using the differential expression analysis of each gene for IO-1 versus SBIO-1 as well as for IO-3

versus SBIO-3. The clustering of the genes in Figure 4C was done using the K-means unsupervised clustering algorithm, as implemented in R. We used Gene Set Enrichment Analysis (GSEA) to analyze the enrichment of the curated gene sets from Molecular Signature Database (MSigDB) v5.2 among differentially expressed genes (Subramanian et al., 2005). The gene expression profiles of the early mouse embryo were obtained from a published study (Boroviak et al., 2015). We merged the expression values for the genes in that study with our samples based on their Entrez IDs. We used the ComBat algorithm of the R/Bioconductor package “sva” for Batch-effect removal (Leek et al., 2012). The values of 1 and 0.01 were the absolute log2-fold change and FDR thresholds for differential expression analyses, respectively, between the *in vivo* (early embryo samples) and *in vitro* (our samples).

Accession numbers

Raw and analyzed microarray data have been deposited in the NCBI Gene Expression Omnibus (GEO) as accession number GSE87793.

Supplemental Tables

Table S1. The list of genes and the expression value of represented heatmaps shown in Figure 3A, related to Figure 3.

Table S2. The list of common pluripotency, R2i pluripotency, and SB pluripotency–related genes based on published data, related to Figure 3.

Table S3. The number of reads and pattern of DNA methylation state between ICM, IOs, and ESC.

Table S4. Comparison of the expressed genes for the *in vivo* embryonic sample (Boroviak et al. 2015) and *in vitro*–cultured ICM-to-ESC samples of this study. Up: Up-regulated; Down: Down-regulated; NS: Non-significant, related to Figure 7.

Table S3. The number of reads and pattern of DNA methylation state between ICM, IOs, and ESC								
Sample	Amplicon	#reads	#CpGs	#mCpG/ mCpG	#mCpG/ CpG	#CpG/ mCpG	#CpG/ CpG	Conversion rate (linker)
ICM	IAPLTR	284	1303	820	129	191	163	0.993
IO-3	IAPLTR	564	2611	1416	373	397	425	0.996
IO-5	IAPLTR	492	2297	1280	344	288	385	0.994
ESC	IAPLTR	510	2329	1815	172	167	175	0.994
ICM	mSat	2514	6809	2443	448	568	3350	0.982
ICM	mSat	2465	6683	2340	369	422	3552	0.976
IO-3	mSat	1624	4393	1761	360	474	1798	0.967
IO-3	mSat	1967	5387	2315	551	493	2028	0.965
IO-5	mSat	2069	5623	2582	437	436	2168	0.967
IO-5	mSat	2338	6307	2932	416	517	2442	0.976
ESC	mSat	2151	5792	2838	378	537	2039	0.969
ESC	mSat	1907	5152	2476	329	464	1883	0.959
ICM	L1	1692	7960	1825	517	460	5158	0.986
ICM	L1	2431	11655	2446	776	546	7887	0.986
IO-3	L1	2177	10444	2354	1077	987	6026	0.984
IO-3	L1	793	3640	907	411	363	1959	0.97
IO-5	L1	2565	12285	3140	1187	1027	6931	0.985
IO-5	L1	1022	4903	1192	509	389	2813	0.988
ESC	L1	1777	8483	2195	942	724	4622	0.985
ESC	L1	2383	11411	2912	1223	1034	6242	0.984

Supplemental References

Arand, J., Spieler, D., Karius, T., Branco, M.R., Meilinger, D., Meissner, A., Jenuwein, T., Xu, G., Leonhardt, H., Wolf, V., Walter, J., 2012. In vivo control of CpG and non-CpG DNA methylation by DNA methyltransferases. PLoS Genet. 8. doi:10.1371/journal.pgen.1002750

Boroviak, T., Loos, R., Lombard, P., Okahara, J., Behr, R., Sasaki, E., Nichols, J., Smith, A., Bertone, P., 2015. Lineage-Specific Profiling Delineates the Emergence and Progression of Naive Pluripotency in Mammalian Embryogenesis. Dev. Cell 35, 366–382. doi:10.1016/j.devcel.2015.10.011

Chen, E.Y., Tan, C.M., Kou, Y., Duan, Q., Wang, Z., Meirelles, G.V., Clark, N.R., Ma'ayan, A., 2013. Enrichr: interactive and collaborative HTML5 gene list enrichment analysis tool. BMC Bioinformatics 14, 128. doi:10.1186/1471-2105-14-128

Fischedick, G., Wu, G., Adachi, K., Araúzo-Bravo, M.J., Greber, B., Radstaak, M., Köhler, G., Tapia, N., Iacone, R., Anastassiadis, K., Schöler, H.R., Zaehres, H., 2014. Nanog induces hyperplasia without initiating tumors. Stem Cell Res. 13, 300–315. doi:10.1016/j.scr.2014.08.001

Han, D.W., Greber, B., Wu, G., Tapia, N., Araúzo-Bravo, M.J., Ko, K., Bernemann, C., Stehling, M., Schöler, H.R., 2011. Direct reprogramming of fibroblasts into epiblast stem cells. Nat. Cell Biol. 13, 66–71. doi:10.1038/ncb2136

Leek, J.T., Johnson, W.E., Parker, H.S., Jaffe, A.E., Storey, J.D., 2012. The SVA package for removing batch effects and other unwanted variation in high-throughput experiments. Bioinformatics 28, 882–883. doi:10.1093/bioinformatics/bts034

Lutsik, P., Feuerbach, L., Arand, J., Lengauer, T., Walter, J., Bock, C., 2011. BiQ Analyzer HT: Locus-specific analysis of DNA methylation by high-throughput bisulfite sequencing. Nucleic Acids Res. 39, 551–556. doi:10.1093/nar/gkr312

Ritchie, M.E., Phipson, B., Wu, D., Hu, Y., Law, C.W., Shi, W., Smyth, G.K., 2015. limma powers differential expression analyses for RNA-sequencing and microarray studies. Nucleic Acids Res. 43, e47. doi:10.1093/nar/gkv007

Subramanian, A., Tamayo, P., Mootha, V.K., Mukherjee, S., Ebert, B.L., Gillette, M. a, Paulovich, A., Pomeroy, S.L., Golub, T.R., Lander, E.S., Mesirov, J.P., 2005. Gene set enrichment analysis: a knowledge-based approach for interpreting genome-wide expression profiles. Proc. Natl. Acad. Sci. U. S. A. 102, 15545–50. doi:10.1073/pnas.0506580102

Yeom, Y.I., Fuhrmann, G., Ovitt, C.E., Brehm, a, Ohbo, K., Gross, M., Hübner, K., Schöler, H.R., Hubner, K., Scholer, H.R., 1996. Germline regulatory element of Oct-4 specific for the totipotent cycle of embryonal cells. Development 122, 881–894.
