# Supplementary material for: Blockage of the Epithelial-to-Mesenchymal Transition Is Required for Embryonic Stem Cell Derivation
Source: Stem Cell Reports. 2017 Sep 14;9(4):1275–90. doi: 10.1016/j.stemcr.2017.08.006 (PMC5639184; doi:10.1016/j.stemcr.2017.08.006)
Supplement: Document S2. Article plus Supplemental Information [file mmc5.pdf]

Stem Cell Reports
Article

OPEN ACCESS

Blockage of the Epithelial-to-Mesenchymal Transition Is Required for Embryonic Stem Cell Derivation

Mehdi Totonchi,[1,2,3,10] Seyedeh-Nafiseh Hassani,[1,3,10] Ali Sharifi-Zarchi,[1,4,10] Natalia Tapia,[5] Kenjiro Adachi,[6] Julia Arand,[7] Boris Greber,[6,8] Davood Sabour,[1,6] Marcos J. Araúzo-Bravo,[9] Jörn Walter,[7] Mohammad Pakzad,[1] Hamid Gourabi,[2] Hans R. Schöler,[6] and Hossein Baharvand[1,3,*]

[1]Department of Stem Cells and Developmental Biology, Cell Science Research Center, Royan Institute for Stem Cell Biology and Technology, ACECR, Tehran, Iran
[2]Department of Genetics, Reproductive Biomedicine Research Center, Royan Institute for Reproductive Biomedicine, ACECR, Tehran, Iran
[3]Department of Developmental Biology, University of Science and Culture, Tehran, Iran
[4]Chitsaz Lab, Department of Computer Science, Colorado State University, Fort Collins 80523, CO, USA
[5]Institute of Biomedicine of Valencia, Spanish National Research Council, Jaime Roig 11, 46010 Valencia, Spain
[6]Max Planck Institute for Molecular Biomedicine, Röntgenstrasse 20, 48149 Münster, Germany
[7]University of Saarland, FR 8.3, Biological Sciences, Genetics/Epigenetics, Campus A2.4, 66123 Saarbrücken, Germany
[8]Chemical Genomics Centre of the Max Planck Society, Dortmung, Germany
[9]Group of Computational Biology and Systems Biomedicine, Biodonostia Health Research Institute, 20014 San Sebastián, Spain
[10]Co-first author
*Correspondence: baharvand@royaninstitute.org
http://dx.doi.org/10.1016/j.stemcr.2017.08.006

SUMMARY

Pluripotent cells emanate from the inner cell mass (ICM) of the blastocyst and when cultivated under optimal conditions immortalize as embryonic stem cells (ESCs). The fundamental mechanism underlying ESC derivation has, however, remained elusive. Recently, we have devised a highly efficient approach for establishing ESCs, through inhibition of the MEK and TGF-β pathways. This regimen provides a platform for dissecting the molecular mechanism of ESC derivation. Via temporal gene expression analysis, we reveal key genes involved in the ICM to ESC transition. We found that DNA methyltransferases play a pivotal role in efficient ESC generation. We further observed a tight correlation between ESCs and preimplantation epiblast cell-related genes and noticed that fundamental events such as epithelial-to-mesenchymal transition blockage play a key role in launching the ESC self-renewal program. Our study provides a time course transcriptional resource highlighting the dynamics of the gene regulatory network during the ICM to ESC transition.

INTRODUCTION

Pluripotency is initiated in cells of the inner cell mass (ICM) and lapses shortly after implantation, coincident with the rise in lineage commitment. *In vitro* culture of ICM permits the generation of stable self-renewing pluripotent embryonic stem cells (ESCs) (Evans and Kaufman, 1981). However, ESC derivation is largely dependent upon the culture conditions. Under conventional medium, containing fetal calf serum and either feeder cells or leukemia inhibitory factor (LIF), only embryos from 129/Sv strain can efficiently give rise to ESCs and most strains of mice are refractory to ESC generation (Brook and Gardner, 1997). It is shown that the 129/Sv strain has intrinsically more preimplantation epiblast (preEpi) cells than primitive endoderm (PE) cells when compared with refractory strains such as C57BL/6 or CBA (Batlle-Morera et al., 2008). So, preventing the formation of PE cells by induction of embryonic diapause (Brook and Gardner, 1997) or use of chemical substances that inhibit Fgf4 signaling (Ying et al., 2008) led to the formation of preEpi cells with efficient capability to generate ESCs even in serum- and feeder-free culture conditions. Also, when pre-blastocyst embryos have been used for ESC derivation, it can be assumed that these embryonic stages develop mainly into preEpi cells that subsequently develop into ESCs (Nichols and Smith, 2011). But, as pluripotent preEpi cells do not exhibit self-renewability, per se, the mechanism underlying this *in vivo* to *in vitro* conversion remains controversial (Loh et al., 2015).

To address the mechanisms underlying ICM to ESC conversion in the conventional culture condition, single-cell RNA sequencing (RNA-seq) analysis showed dramatic transcriptional and epigenetic gene expression changes during ICM to ESC transition (Tang et al., 2010). These changes include the simultaneous downregulation of *Pramel5/6/7*, *Gata6*, and *Cdx2* and upregulation of *Dnmt3a* and *Hdac5*, reflecting the higher expression of epigenetic modifiers during this cell transition process (Tang et al., 2010). This study suggested that reprogramming is a major event in the cell fate conversion of ICM to ESCs. However, this mechanism may be overshadowed by the low efficiency of generating ESCs from refractory mouse strains under serum-based culture conditions, as well as the tendency of the generated cells to undergo differentiation and their propensity to heterogeneously express key naive pluripotency transcription factors (TFs) (Hassani et al., 2012).

Recent single-cell transcriptome analyses of mouse early embryos have demonstrated a high similarity between

© 2017 The Author(s). This is an open access article under the CC BY license (http://creativecommons.org/licenses/by/4.0/).
CrossMark

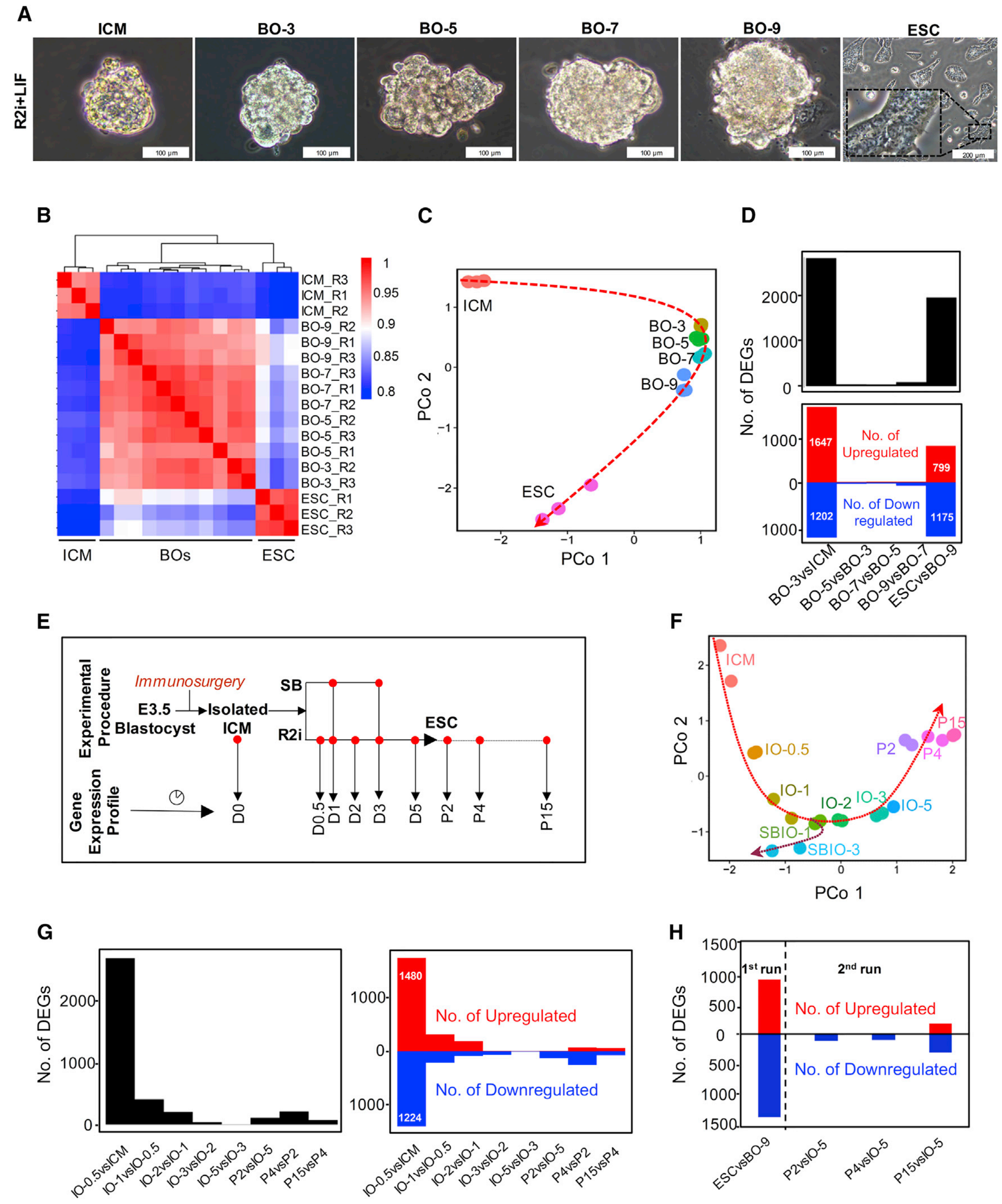

Figure 1. High-Resolution Transcriptome Trajectories from ICM to ESCs

(A) Isolated ICM and whole-blastocyst outgrowths (BOs) on days 3, 5, 7, and 9, and ESCs (in passage 20).

(B) Unsupervised hierarchical clustering of time course gene expression profiles for ESC derivation. Three independent biological replicates (nearly 20–30 ICMs and BOs for each replicate) were used except for BO-3 with two biological replicates. Transcript levels are mean-centered $\log_2$ scale values.

(C) Principal coordinate analysis (PCoA) of time course gene expression profiles. The red arrow shows the trajectory of cell expression profiles during ESC derivation.

(D) The number of differentially expressed genes (DEGs) between each of the consecutive time points. Red and blue bars indicate up- and downregulated genes, respectively.

(legend continued on next page)

preEpi cells and ESCs grown in 2i (chemical inhibitors of MEK and GSK3) (Boroviak et al., 2015). The 2i condition supports the ground state of pluripotency, and triggers the efficient derivation and long-term maintenance of transcriptionally homogeneous ESCs. It has thus been suggested that 2i-grown ESCs are the actual self-renewing counterpart of naive preEpi cells, and that all preEpi cells are probably capable of becoming ESCs under 2i treatment (Boroviak et al., 2015).

We have recently introduced another efficient method for establishing ESCs from different refractory and non-permissive mouse strains by using PD0325901 and SB431542, chemical inhibitors of MEK, and transforming growth factor β (TGF-β) signaling pathways, respectively, which we named R2i (Hassani et al., 2014b). We showed that in the serum-free condition, R2i could support the ground state of pluripotency. Also, as well as from blastocysts, R2i could support the highly efficient ESC derivation from a wider developmental window, from single blastomeres of two to eight cell-stage mouse embryos with an efficiency of more than 2-fold higher than the 2i condition (Hassani et al., 2014a). Moreover, when cells underwent long-term passaging, they exhibited more genomic stability with R2i support compared with 2i (Hassani et al., 2014b). These salient features of R2i prompted us to evaluate the molecular mechanism(s) that underlie ICM to ESC transition. By conducting a gene expression profile at serial time points during the transition from ICM to ESCs, we discovered key regulatory genes and cellular events that are involved in this process. Besides the expression of preEpi-related genes, we found that the upregulation of DNA methyltransferases and an epithelial-to-mesenchymal transition (EMT) blockage probably have major impacts on the efficient generation of ESCs. This study reveals mechanistic insight into how transient pluripotent cells from the preimplantation embryo can perpetuate as self-renewing ESCs.

RESULTS

Sampling Strategy for Gene Expression Profiling during the Transition from ICM to ESCs

To establish a gene expression signature throughout the course of ESC derivation, we initially collected samples from immunosurgically isolated ICMs from blastocysts at embryonic day (E) 3.5 and whole-blastocyst outgrowths (BOs) on days 3, 5, 7, and 9 post-plating, as well as from ESCs growing under R2i + LIF (R2i) conditions (Figure 1A). The mRNA extracted from these cells was pre-amplified and used for whole-genome analysis using the Illumina platform. Pairwise Pearson coefficient of the gene expression profiles of samples indicated a significant difference between the ICM cells and ESCs. Hierarchical clustering revealed three major clusters of ICMs, BOs, and ESCs (Figure 1B). Dimension reduction of gene expression data by principal coordinate analysis (PCoA) depicted a molecular trajectory that reflected the transitional cascades from ICM cells to ESCs (Figure 1C). Next, we examined the differentially expressed genes (DEGs) at the indicated time points. We found 2,849 DEGs that indicated alterations (1,647 up- and 1,202 downregulated) between ICM cells and BO-3, and 1,974 DEGs (799 up- and 1,175 downregulated) between BO-9 and ESCs ($\geq$2-fold expression change, adjusted p $\leq$ 0.05) (Figure 1D). We performed an unsupervised time course of gene clustering followed by gene set analysis using the Enrichr web tool (http://amp.pharm.mssm.edu/Enrichr/; Figure S1A). We found evidence for pluripotency and the establishment of self-renewal in ESCs, as indicated by related functional annotation such as PluriNetWork and mitotic cell cycle in clusters IV and V, respectively. However, our data documented an alteration in gene expression between two major phases of the ICM to ESC transition. The first phase was detectable in the early stage of the process, between ICM cells and BO-3, whereas the second phase occurred in the late stage, between BO-9 and ESCs (Figures 1B and 1C).

We assumed that a 3-day gap between ICM cells and the first collected BOs almost hindered accurate interpretation of the extracted data. Furthermore, even though trophectoderm cells were not expanded (Figure 1A), the few such cells that existed probably resulted in gene alterations from BO-9 to ESCs. This result convinced us that a higher-resolution time course analysis from isolated ICM outgrowths (IOs) would give us better insight into the molecular cascades occurring during the cellular transition. Therefore, we collected immunosurgically isolated ICM cells and IOs on days 0.5, 1, 2, 3, and 5 post-plating

(E) Schematic illustration of the overall sample collection time points for gene expression profiling of ESC line derivation for the second run of microarray analysis. Two culture conditions were used: treatment (R2i) and control (SB). The samples (two biological replicates) included immunosurgically isolated ICMs (day 0), ICM outgrowths (IOs) on days 0.5, 1, 2, 3, and 5, and ESCs of early (P2 and P4) and late (P15) passages.
(F) PCoA of the time course transcriptome profiles. The red dotted arrows show the cell-state trajectories and the bifurcation point between R2i and SB groups.
(G) The number of DEGs during the indicated time points is shown on the left and significantly upregulated (red bars) and downregulated (blue bars) genes between each pair of consecutive time points are shown on the right.
(H) Comparison of the number of DEGs in the first and second run of microarray for BOs versus ESCs (in the first run), and for IOs versus ESCs (in the second run).

A

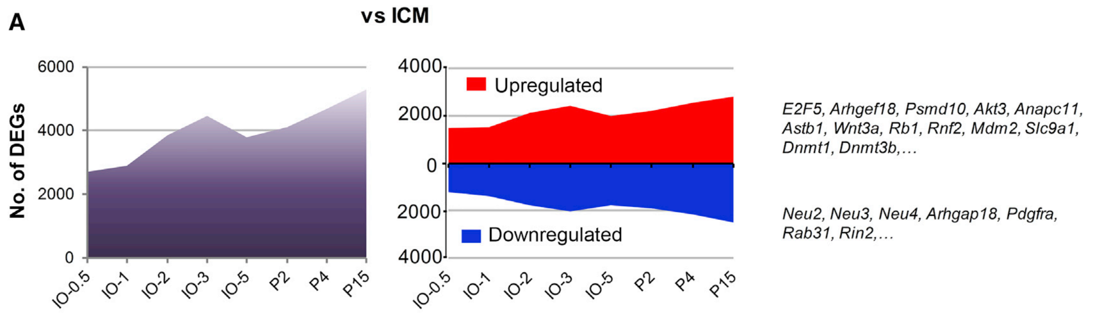

B

	IO-0.5 vs. ICM			Other stages vs. ICM					
	Rank	Functional annotation	*p* value	Functional annotation	Rank(*p* value)				
					IO-1	IO-2	IO-3	IO-5	ESCs (P2)
Upregulated	1	GO terms<br>- Mitotic cell cycle	5.76E-06	GO terms<br>- Mitotic cell cycle	1(2.95E-06)	1(6.42E-09)	1(6.99E-09)	1(1.59E-09)	1(7.57E-11)
Upregulated	2	KEGG pathway<br>- Small cell lung cancer	7.03E-03	KEGG pathway<br>- Small cell lung cancer	2(1.00E-03)	1(8.99E-05)	1(6.49E-06)	6(9.55E-05)	3(1.38E-04)
Downregulated	1	GO terms<br>- Membrane lipid catabolic process	1.26E-04	GO terms<br>- Membrane lipid catabolic process	6(1.09E-03)	2(1.06E-03)	12(2.58E-03)	7(1.20E-03)	2(4.07E-04)
Downregulated	1<br>5	KEGG pathway<br>- Other glycan degradation<br>- Metabolic pathways	2.89E-04<br>8.26E-03	KEGG pathway<br>- Other glycan degradation<br>- Metabolic pathways	3(2.07E-03)<br>5(6.91E-03)	3(1.97E-03)<br>1(6.97E-03)	3(4.36E-03)<br>1(1.22E-04)	4(2.26E-03)<br>1(9.19E-06)	5(2.89E-03)<br>1(2.00E-04)

C

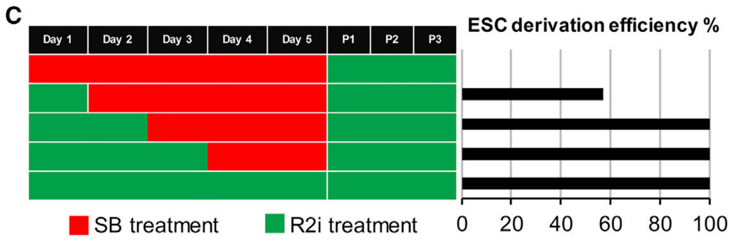

D

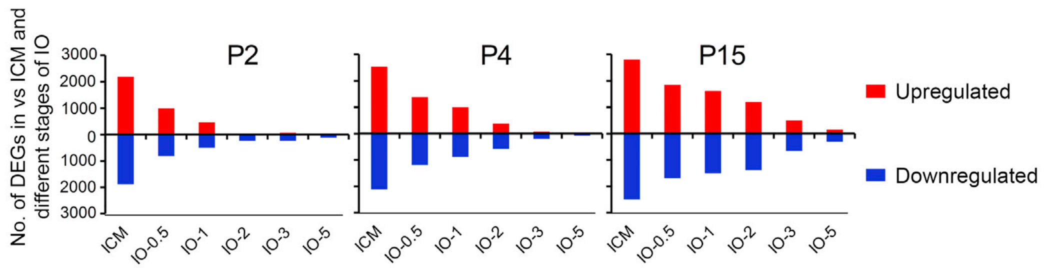

E

Upregulated

P15 vs	GO terms	*Rank*(*p* value)
ICM	- mitotic cell cycle	1(4.99E-11)
IO-0.5	- regulation of cellular response to stress<br>- DNA repair	1(1.12E-06)<br>2(1.03E-06)
IO-1	- DNA repair<br>- mitotic cell cycle	1(1.27E-07)<br>2(2.86E-07)
IO-2	- organelle fission<br>- nuclear division	1(5.22E-11)<br>2(8.28E-11)
IO-3	- positive regulation of macroautophagy	1(4.17E-04)
IO-5	- organelle fission<br>- nuclear division	1(5.7E04)<br>2(3.09E-04)

Downregulated

P15 vs	GO terms	*Rank*(*p* value)
ICM	- translation<br>- gene expression	1(2.77E-08)<br>2(4.57E-06)
IO-0.5	- translation<br>- gene expression	1(4.37E-14)<br>2(4.64E-10)
IO-1	- translation<br>- gene expression	1(2.25E-12)<br>2(8.34E-10)
IO-2	- translation<br>- gene expression	1(1.27E-08)<br>2(3.69E-08)
IO-3	- gene expression	1(1.86E-05)
IO-5	- regulation of epithelial cell migration	1(3.63E-08)

(legend on next page)

(Figure 1E). In these analyses, we limited the period of study to day 5 due to a high similarity between the different time points of BO samples in the first run of the microarray. In addition, we used ESCs of different passage numbers (P2, P4, and P15) in our analyses. The negative control group for these experiments was the cultured IOs under SB + LIF (SB) conditions on days 1 and 3 post-plating (SBIO-1 and SBIO-3, respectively; Figure 1E). This control group was based on our previous observation that each component of R2i (PD or SB) in combination with LIF supports the long-term maintenance of ESCs. However, the efficiency of deriving ESCs under SB + LIF conditions was negligible or low, whereas it approximated 60% under PD + LIF (Hassani et al., 2014b). PCoA of the time course data depicted a parabolic trajectory of the ICM to ESC derivation process that bifurcated under SB conditions (Figure 1F). This analysis showed that the profile in SBIO-1 was close to the ICM to ESC trajectory, whereas SBIO-3 significantly differed from IO-3. We observed the highest number of DEGs in the sequential time points between IO-0.5 and ICM cells, at 2,704 altered genes (1,480 upregulated and 1,224 downregulated; adjusted $p < 0.01$; fold change >2; Figure 1G). This tangible gene alteration between ICM cells and IO-0.5 was reminiscent of the high number of DEGs between the ICM cells and BO-3 in the first run of our experiments. We observed that the alternations among consecutive samples are biologically relevant rather than a fluctuation among replicates (Figure S1B). Therefore, our study denoted a significant expression fluctuation from *in vivo* to *in vitro*, even in the short time period after this cultivation. However, the number of DEGs between late-stage outgrowths and ESCs of different passage numbers was dramatically dropped in the second set of experiments (Figure 1H). Overall, this new design for sampling provided a convenient platform for assessing the key events involved in the generation of ESCs from ICM cells.

A Gradual Molecular Transition from ICM to ESCs

Our first priority was to uncover the reason for the tremendous difference in the number of DEGs between the ICM cells and IO-0.5. Gene set analysis showed the reasonable enrichment score for genes involved in the mitotic cell cycle and membrane lipid catabolism for up- and downregulated genes, respectively (Figures 2A, 2B, and S2A). We sought to determine whether the gene expression alteration in the first interval could cause the establishment of ESCs, or only represented a stochastic fluctuation of gene expression during *in vivo* to *in vitro* transition. Therefore, we compared the expression profile of IOs from the other designated time points with the ICM cells (Figure 2A). This analysis revealed an upward trend in the number of DEGs for IOs with an increased time point interval and the ICM cells (Figure 2A). However, most of the altered genes revealed the same functional annotation identified in the comparison between ICM cells and IO-0.5, as well as pathways for small-cell lung cancer and relevant metabolism with regard to the up- and downregulated genes, respectively (Figures 2B and S2A). This representation of biological processes during the ICM to ESC transition appeared to be consistent with the acquisition of ESC self-renewal capability.

As the early evidence of self-renewal capability was found during ICM to IO-0.5 transition (Figures 2A and 2B), we sought to determine whether ESC identity was acquired quickly after ICM expansion *in vitro*. Therefore, we examined the temporal dependency on R2i culture conditions to determine the minimal time required for the efficient derivation of ESCs. We found that 1-day treatment with R2i led to the derivation of ESCs at approximately 60% efficiency, and that 2-day treatment induced ESCs at maximal derivation efficiency (Figure 2C). This finding revealed that the gene expression changes taking place during the first days of ICM culture supported the efficient establishment of ESCs. In the next step, we compared the expression profile of ESCs of different passages (P2, P4, and P15) with ICM cells and all the IOs (Figures 2D and 2E). The results showed that, with increasing passage number, ESCs are closer to late-stage IOs than to early-stage IOs (Figures 2D and 2E). Functional analysis of DEGs between IOs and ESCs at different passage numbers indicated the upregulation of nuclear division-related genes and the downregulation of genes involved in translation and gene expression processes. However, these differences declined as the interval between ESCs and IOs was reduced (Figures 2E and S2B–S2D). Nearly similar functional annotations were enriched by analyzing the differences in gene expression between ESCs of different passages (Figure S2E). Despite the high similarity between ESCs and late-stage

Figure 2. Transition from ICM to ESCs Is a Gradual Process

(A) The representation of DEGs between different stages of IOs and ESCs versus ICM.

(B) Functional annotation of up- and downregulated genes between IOs and ESCs of different stages versus ICM.

(C) Experimental schematic and the results of time course dependency of the derived ESCs under R2i culture conditions. For each experiment (rows), the red and green bars indicate the duration of time the cells were cultured in the negative control and R2i media, respectively. The efficiency of deriving ESCs is based on the number of Nanog-positive colonies derived from ten isolated ICM.

(D) The number of up- and downregulated genes between ESCs and R2i-treated IOs.

(E) Functional annotation of up- and downregulated genes between P15 and IOs of different stages.

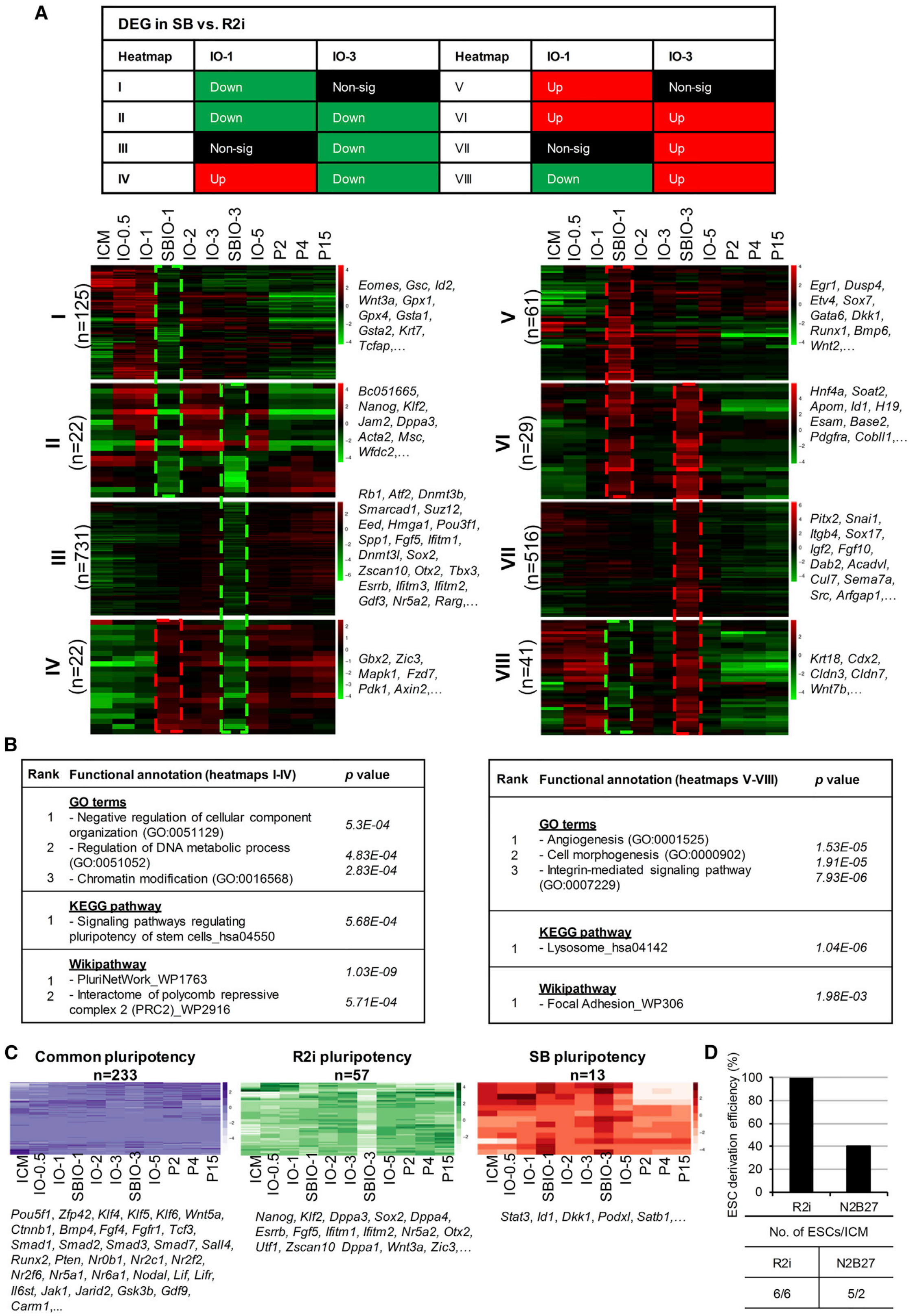

(legend on next page)

IOs, functional analysis for downregulated genes highlighted the importance of pathways related to epithelial cell differentiation, organ formation, extracellular matrix organization, and regulation of epithelial cell migration (Figures S2B–S2D and 2E). This result suggested that preventing the differentiation or migration of epithelial cells should be considered for successfully establishing an immortal ESC line. Therefore, it appeared that, although key changes in gene expression occurred quite quickly during the conversion of ICMs to ESCs, cells had assumed an ESC identity rather gradually. These findings suggested the existence of a distinct core regulatory circuitry for the establishment of ESCs. This circuitry would form and be active promptly during the *in vitro* ICM culture on one hand, but would induce cells to acquire ESC identity gradually on the other hand.

Identification of Transcriptional Signature in IOs

To identify the genes that play a key role in the ICM to ESC transition process, we categorized DEGs in both the R2i and control (SB) groups. We generated the gene expression profile of the SB group at two serial time points (SBIO-1 and SBIO-3) and subsequently compared their DEGs with the R2i group at the same days (IO-1 and IO-3). The outcome consisted of eight heatmaps (Figure 3A, Table S1). Heatmaps I–IV showed the list of genes that were downregulated in at least one of the aforementioned time points in the SB versus R2i groups, while heatmaps V–VIII showed the upregulated genes (Figure 3A). Pathway enrichment analysis indicated that most genes in heatmaps I–IV were associated with signaling pathways that regulated pluripotency, chromatin modification, and DNA metabolism. Specifically, heatmap III contained the largest number of DEGs, which revealed several pluripotency markers such as *Esrrb, Sox2*, *Tbx3*, and *Rarg*, and epigenetic modifiers including *Wdr3*, *Suz12*, *Ezh2*, *Eed*, *Nr5a2*, *Dnmt3b*, and *Dnmt3l* (Figures 3A, 3B, and S3A). Thereafter, we denoted the list of genes in heatmaps I–IV as R2i-specific and downregulated in SB groups, suggesting that these genes play an important role on the road to pluripotency. In contrast, heatmaps V–VIII (SB-specific gene list) highlighted mainly developmental pathways that included cell morphogenesis, cell-cell adhesion, and protein processing. In these gene lists, the greatest numbers of DEGs represented in heatmap VII contained some key upregulated factors such as *Pitx2*, *Snai1*, *Itgb4*, *Sox17*, *Igf2*, *Fgf10*, and *Dab2*, suggesting that the SB-treated IOs exited from the path of pluripotency (Figures 3A, 3B, and S3A).

Interestingly, our result depicted that the upregulation of some relevant pluripotency-related markers was observed in the SB-specific gene list, which included *Stat3*, *Dusp9*, and *Id1*, downstream of LIF and BMP4, respectively. This finding raised the question of whether TGF-β inhibition could sustain the expression of some pluripotency-related genes during the ICM to ESC transition. To address this question, we classified the expression profile of a comprehensive list of pluripotency-related genes (366 genes extracted from the PluriNetWork and the literature; Table S2) into common pluripotency, R2i-specific pluripotency, and SB-specific pluripotency groups (Figure 3C). A total of 233 well-known TFs that regulated pluripotency was put in the common pluripotency list, which included *Pou5f1* (*Oct4*), *Zfp42* (*Rex1*), *Klf4*, *Ctnnb1* (β-*Catenin*), and *Sall4*. In addition, 13 of 366 genes were in the SB-specific pluripotency list, which included *Stat3*, *Id1*, *Dkk1*, and *Podxl*. This analysis demonstrated that TGF-β inhibition, like fibroblast growth factor (FGF) signaling inhibition, plays a pivotal role in supporting the dynamic expression of pluripotency-related markers, while SB alone was insufficient for ESC derivation. It has been proposed that some of the pluripotency factors induce differentiation and play a central role in the determination of different cell states by orchestrating different gene expression profiles (Loh et al., 2015). Consistent with this notion, co-occupation of various sites with Smad2/3 and different master TFs has been found to direct different responses in ESCs, myoblasts, and B cells (Mullen et al., 2011). In contrast, R2i-specific pluripotency markers, such as *Nanog*, *Klf2*, *Dppa3*, *Sox2*, *Esrrb*, *Utf1*, and *Nr5a2*, have unveiled key regulators in the path to efficiently deriving ESCs from ICM. We showed that 5 (*Esrrb*, *Gbx2*, *Sox2*, *Klf2*, and *Nanog*) of 12 essential TFs for naive pluripotency (Dunn et al., 2014)

Figure 3. Transcriptome Signature of IOs

(A) Categorization of DEGs for IOs on days 1 and 3 between the R2i and SB groups (IO-1 versus IOSB-1 and IO-3 versus IOSB-3). The upper panels show the pattern of this classification for the below heatmaps. Heatmaps I–IV represent the pattern of genes upregulated in R2i. Heatmaps V–VIII represent the pattern of genes downregulated in R2i versus SB. The related genes for each cluster are shown. Green and red dashes show significant down- and upregulated genes in SB versus R2i, respectively.

(B) Functional annotation of heatmaps I–IV (R2i-specific genes) and V–VIII (SB-specific genes). The high ranking of gene ontology (GO) analysis, overrepresented KEGG pathways and WikiPathway are shown.

(C) A set of pluripotency-related genes shared between R2i- and SB-treated IOs (common pluripotency) or specific to R2i pluripotency and SB pluripotency.

(D) Efficiency of deriving ESCs upon *Nanog* overexpression. A Tet-On Nanog-inducible 3.5-day blastocyst from F1 hybrid × OG2 mice was used for this study.

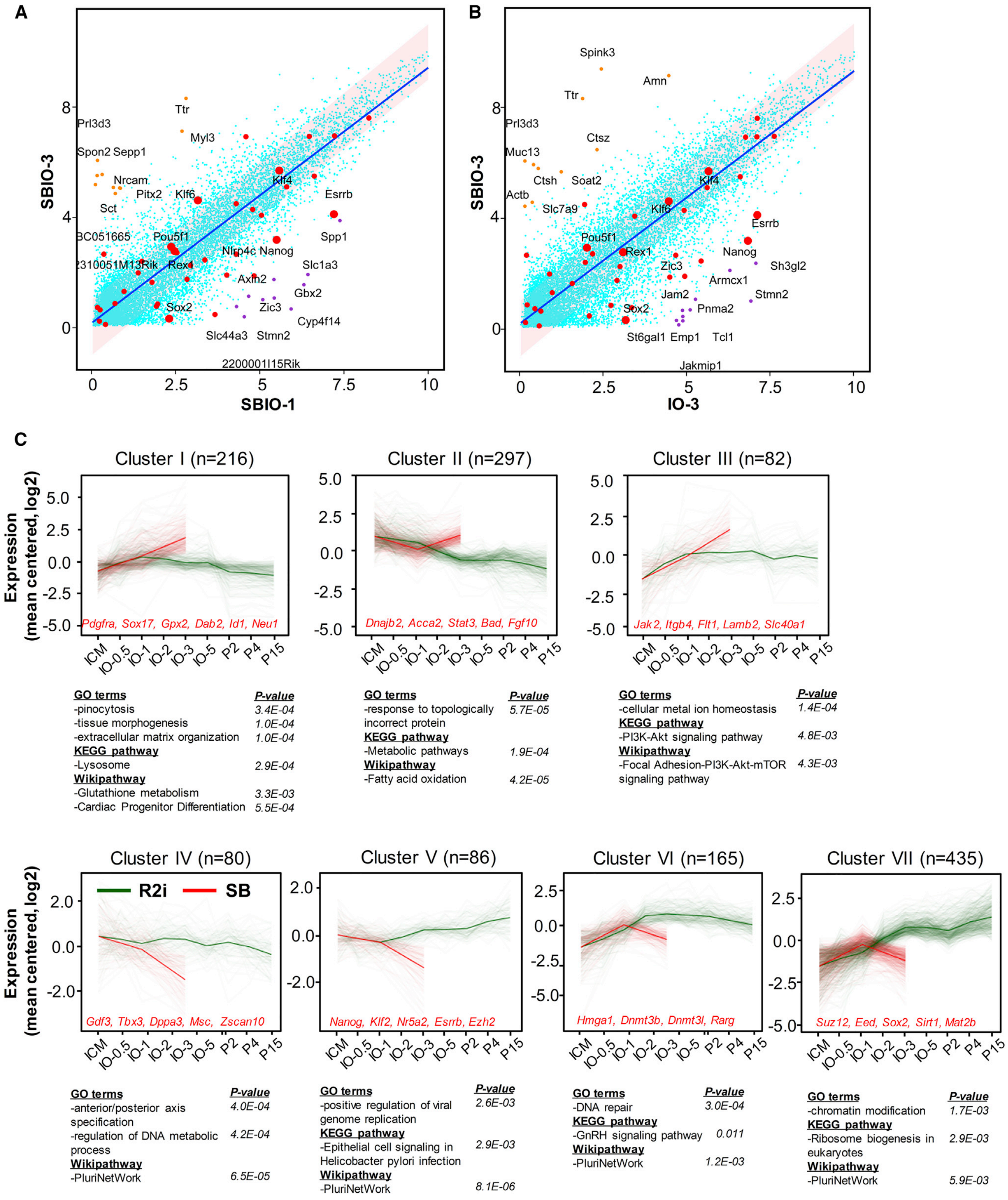

(legend on next page)

were in the R2i-specific pluripotency list, suggesting that they play key roles during ESC derivation (Figure S3B). To test this finding, we overexpressed *Nanog* in the ICM cells of E3.5 blastocysts obtained from *Nanog*-inducible mice in order to generate ESCs in N2B27 medium in the absence of SB or PD, resulting in 40% ESC derivation efficiency (Figure 3D). These data indicated that our strategy to classify the expression of pluripotency markers during the expansion of ICM could point to the key players involved in ESC derivation.

Transition in DNA Methylation Level during the ICM to ESC Conversion

As R2i-specific pluripotency markers significantly presented in day 3 (Figures 4A and 4B), we sought to classify the gene expression patterns by performing an unsupervised time course clustering on DEGs between SBIO-3 and IO-3 (Figure 4C). Clusters I–III represented the upregulated genes in SBIO-3 compared with IO-3, which exhibited different expression patterns in the R2i-treated group. Gene ontology analysis highlighted the increased activities of several biological processes that were associated mainly with tissue morphogenesis, metabolic pathways, and focal adhesion (Figure 4C). In contrast, clusters IV–VII represented downregulated genes in the SBIO-3. Gene enrichment analysis revealed that the genes in clusters IV–VII were associated with PluriNetWork, the DNA metabolic process, and chromatin modification. Clusters IV (80 genes) and V (86 genes) contained some key TFs for pluripotency (*Tbx3*, *Esrrb*, and *Nanog*), which were most likely expressed in a steady-state pattern from ICM cells to ESCs. Clusters VI (165 genes) and VII (435 genes) consisted of parabolic and gradually upregulated patterns of genes from ICM cells to ESCs, which included some epigenetic modifiers such as *Dnmt3b*, *Dnmt3l*, *Chd8*, *Mtss1*, *Suz12*, *Eed*, *Wdr3*, and *Mat2b* (Figure 4C). To better understand the expression dynamics of epigenetic modifier genes during the derivation of ESCs, we conducted qRT-PCR analysis to quantify the expression of *Dnmt1*, *Dnmt3b*, *Dnmt3l*, *Sirt1*, *Ezh2*, *Suz12*, and *Mat2b* (Figure 5A). This experiment confirmed that the expression of *Dnmt1*, *Dnmt3b*, and their partners, was upregulated in this cell transition process. To examine the DNA methylation pattern, we measured the methylation level of the individual CpG sites for three classes of repetitive elements by deep hairpin-bisulfite sequencing. As approximately 40% of the genome consists of repetitive elements, we selected major Satellites (mSat), the 5′ UTR of L1Md_Tf (L1), and a class of LTR-retrotransposons (IAP-LTR1) (Arand et al., 2012). Our data showed that the DNA hyper-methylation during ESC derivation was due mainly to increased amounts of hemi-methylation at CpG positions (Figure 5B). We observed the highest amount of non-CpG methylation at mSat in R2i-treated IOs (Figure S4; Table S3). Non-CpG methylation depended on the presence of Dnmt1 mediated by Dnmt3a/3b in ESCs under serum conditions (Arand et al., 2012). This finding showed that early changes in DNA methylation probably occurred in tandem with an increased activity in *de novo* methyltransferases.

Next, we sought to determine the impact of increased global DNA methylation levels on the transition between the *in vivo* to *in vitro* pluripotent cell states. Inhibition of DNA methyltransferases by RG108 led to reduced efficiency of deriving ESCs under R2i conditions, but had no adverse effect under 2i conditions (Figure 5C). It had previously been reported that 2i could induce global hypomethylation (Ficz et al., 2013; Leitch et al., 2013); however, our results indicated the upregulation of *de novo* methyltransferases for the efficient establishment of ESCs in R2i. Unexpectedly, this finding implied that blockage of the ERK pathway, in parallel with TGF-β inhibition, led to a high expression of epigenetic modifiers and DNA methylation-related genes in the transition from ICM to ESCs. This analysis revealed a major difference between 2i- and R2i-grown ESCs, suggesting the existence of a different pluripotency circuitry of these two naive representative conditions.

Blocking EMT Is Required for ESC Derivation

Our gene expression profile analysis highlighted a difference between ESCs and IOs in epithelial differentiation and migration (Figures 2E and S2B–S2D). Interestingly, a gene set enrichment analysis between DEGs from R2i- and SB-treated IOs displayed an enrichment of EMT-related genes (Figure S5A). Thus, we sought to assess whether EMT might play a critical role in ESC derivation. To this end, the expression levels of genes involved in EMT and mesenchymal-to-epithelial transition (MET), such as *Snail*, *Eomes*, *Dab2*, *Cdh1*, and *Klf4*, as well as key pluripotency genes, were analyzed in R2i-treated IOs using qRT-PCR, and serum + LIF-treated IOs were used as a negative control (Figure 6A). Our results showed an upregulation of epithelial

Figure 4. Day 3 Outgrowths Represented the Highest Difference between R2i and SB Groups

(A and B) Scatterplots showing the gene expression profiles of (A) SBIO-3 versus SBIO-1 and (B) SBIO-3 versus IO-3. The trend is shown as a blue line. The pink ribbon shows genes that have less than 2-fold expression changes between the two samples ($p < 0.05$).

(C) Unsupervised clustering of gene expression for DEGs for IOs on day 3 between treatment (R2i) and control (SB) groups. Clusters I–III represent the pattern of genes that are downregulated in IO-3 versus SBIO-3. Clusters IV–VII represent the pattern of genes that are upregulated in IO-3 versus SBIO-3.

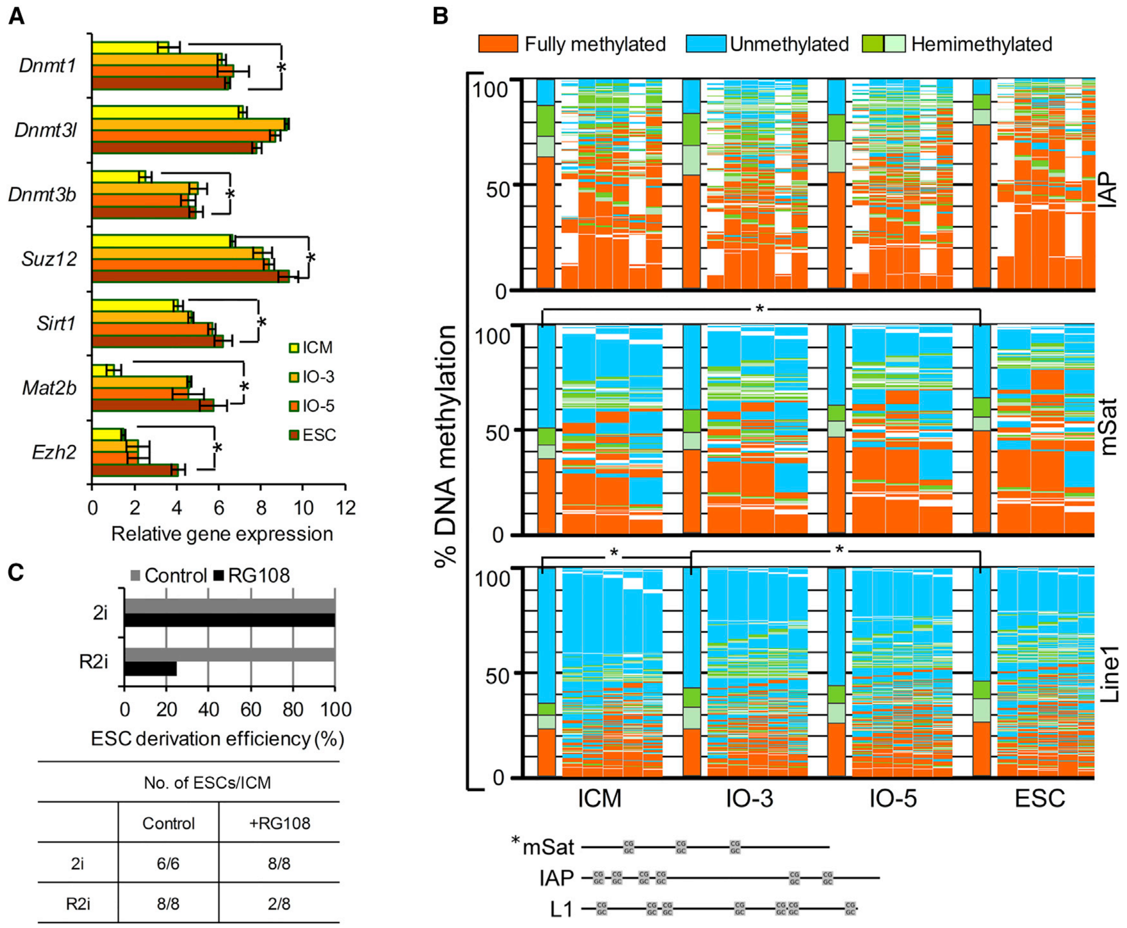

No. of ESCs/ICM		
	Control	+RG108
2i	6/6	8/8
R2i	8/8	2/8

Figure 5. DNA Methylation Changes during ESC Derivation

(A) Time course expression of epigenetic modifier genes during ESC derivation using qRT-PCR. Three biological replicates were used for this experiment. Error bars represent the SD. Here, * represents significant DEGs between ICM and ESCs, $p \leq 0.01$. Statistical analysis was performed by one-way ANOVA and *post hoc* Tukey test.

(B) Hairpin-bisulfite amplicon sequencing of CpG dyads at retrotransposable elements (L1Md_tf, IAP-LTR1, and mSat). The bars sum up the DNA methylation status of all CpG dyads. The map next to the bar represents the distribution of methylated sites. Each column shows neighboring CpG dyads and each line represents one sequence read. The reads in the map are sorted first by fully methylated sites, then by hemi-mCpG dyads. Red, fully methylated; light green and dark green, hemi-mCpG; blue, unmethylated CpG. Two biological replicates for IAP-LTR1 and mSat and one biological replicate for L1Md_tf were used and * represents significant difference of DNA methylation between ICM, IOs, and ESCs, $p \leq 0.01$. The numbers of reads and methylation state are shown in Table S3.

(C) Efficiency of ESC derivation upon inhibition of DNA methyltransferases by using RG108 in R2i- and 2i-treated IOs.

markers and a downregulation of mesenchymal markers in R2i-treated IOs compared with the negative control. Indeed, *Snail*, *Eomes*, and *Dab2* exhibited higher expression levels in the presence of serum, correlating with a decrease in ESC derivation efficiency and an increase in differentiating IOs (Figure S5B). Next, we decided to ascertain whether blocking EMT is required for ESC derivation. To this end, we overexpressed *Snail*-2A-Tdtomato (EMT inducer) and *Klf4*-2A-Tdtomato (MET inducer) in R2i- and SB-treated IOs. In the presence of Snail, IOs started to differentiate and ESCs could not be derived under R2i conditions (Figures 6B and 6C). In contrast, 14 of 14 ESC lines were established from R2i-treated IOs overexpressing Tdtomato alone as a control. Conversely, *Klf4* overexpression led to a 30% efficiency in ESC derivation in SB-treated IOs (Figures 6D, 6E, and S5C), whereas no ESC lines were derived in the Tdtomato control under the same conditions. Finally, we noticed that supplementation of 2i with TGF-β1 impeded the transition from ICM cells to ESCs (Figure 6F). Taken together, these findings demonstrate the crucial role of EMT blockage for the ICM to ESC transition. Contrary to what was expected, we showed that TGF-β inhibition induced EMT, while with the synergistic effect of MEK inhibition blocked EMT. Overall, our data

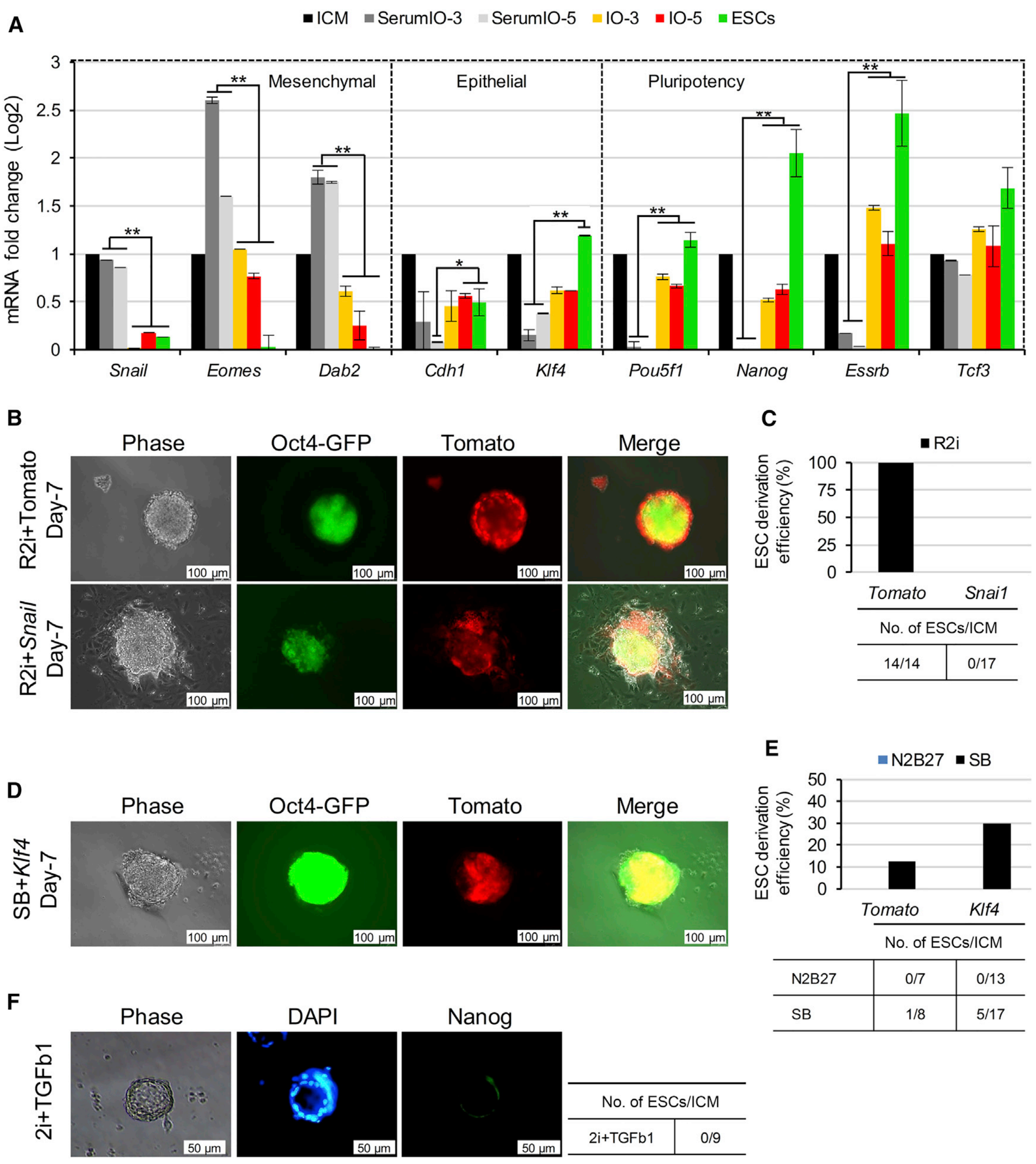

Figure 6. Blocking EMT Is Required for ESC Derivation

(A) Time course expression of EMT and MET gene markers was measured by qRT-PCR in R2i- and serum + LIF-treated IOs. All data were calibrated to ICM, which is considered 1. Error bars reflect SD of results derived from three biological replicates. Here, * represents significant deferentially expressed genes in R2i- and serum + LIF-treated IOs as well as ESCs. *$p \leq 0.05$, **$p \leq 0.01$, error bar, ± SD.

(B) Overexpression of *Snail*-2A-tdTomato in OG2 ICM, which contain an *Oct4*-GFP reporter transgene, with R2i treatment. A construct with tdTomato alone was used as a negative control. Induction of EMT through the forced expression of *Snail* impaired ICM to ESC transition. tdTomato expression correlates with the transduction rate and expression of the gene of interest.

(C) Efficiency of ESC derivation upon *Snail* overexpression.

(D) Overexpression of *Klf4*-2A-tdTomato on E3.5 ICM (B6 × C3H) F1 in N2B27 supplemented with SB + LIF prevents EMT and supports the derivation of ESC lines.

(E) Efficiency of ESC derivation upon *Klf4* overexpression on SB-treated IOs.

(F) The impact of TGF-β1 on the generation of ESCs was assessed in 2i-treated IOs after NANOG immunostaining. Blue, DAPI.

demonstrate that blocking EMT is an essential step in the ESC derivation process. When EMT blocking occurs, a TGF-β inhibitor (SB) combined with LIF could support the long-term maintenance of ESC self-renewal (Hassani et al., 2014b).

R2i Could Maintain preEpi Cell Identity in IOs

We also conducted a parallel transcriptional study on the development of ICM cells in both *in vitro* and *in vivo* conditions. Recently, Boroviak et al. (2015) reported single-cell RNA-seq data for the lineage-specific profile of naive pluripotency in early embryogenesis. However, those authors had adopted their protocol to small group of cells, instead of single cells, to be able to detect scarce transcripts, which provided a good substrate for comparison with our bulk transcriptome data. We observed a range of different early embryo lineage-feature gene expressions, from the ICM to post-implantation cells in R2i-treated IOs (Figure 7A; Table S4). Our study revealed a maximum similarity of different IOs and early-passage ESCs (P2 and P4) to E4.5 preEpi cells. Late-passage ESCs (P15) showed slightly less similarity to the E4.5 preEpi cells (Figure 7B), while functional analysis of up- and downregulated genes in this comparison highlighted the similar pathways (Figure S6A) that were enriched between P15 ESCs and different IOs (Figures 2E and S2D).

A comparison of R2i and SB treatment revealed that the number of ICM-related genes decreased in R2i- versus SB-treated IOs, while the number of gene markers related to preEpi and postEpi cells increased (Figure S6B). This analysis indicated the exclusive expression of diapaused-specific markers, such as *Tcfap2c*, *Gbx2*, *Utf1*, *Sox2*, *Esrrb*, *Tbx3*, *Klf2*, *Nanog*, *Dppa3*, and *Msc*, in R2i-specific clusters (Figures S6B and S6C). We also found that the expression of nearly one-third of the genes in heatmap II in Figure 3A were comparable with preEpi- and diapaused-related indicators, suggesting that the expression of these genes is important throughout the entire process of ESC derivation (Figure 7C).

We then compared our finding with a previously reported dynamic single-cell transcriptional dataset for identifying markers during the preEpi and PE bifurcation of the ICM (Gerovska and Arauzo-Bravo, 2016). We found that R2i-treated IOs noticeably represented the expression profile of E4.5 preEpi cells, while SB-treated IOs showed the different expressed genes such as *Sox17*, *Sox7*, and *Gata6*, which indicated an expression pattern of E4.5 PE (Figures S6D and S6E). In particular, one-third of genes in cluster VI, which were downregulated in R2i versus SB on both days 1 and 3, represented the PE transcriptome profile. A comparison of different staged IOs and early-stage embryos revealed significantly expressed PGC markers, which included *Ifitm3*/*Fragilis*, *Dppa3*/*Stella*, *Zp3*, *Ifitm1*, and *Ifitm2* (Boroviak et al., 2015; Gerovska and Arauzo-Bravo, 2016), as well as *Tex11*, *Tex14*, *Piwil2*, and *Dppa1* (Figure 7D). This observation not only elucidated the preEpi cell origin of ESCs but also magnified the role of PGC-related genes during the ICM to ESC transition. Overall, this dataset depicted that ICM-derived ESCs proceeded through the preEpi stage, and that the successful derivation of ESCs relied on FGF inhibition. However, epigenetic modifications and EMT blockage were required for the perpetual self-renewal capability of preEpi cells.

DISCUSSION

An optimized regimen is required for dissecting the molecular mechanism of ESC derivation. Dual inhibition of FGF and TGF-β creates high genomic stability and an efficient ICM to ESC transition (Hassani et al., 2014b). We have employed this system to produce a temporal transcriptome profile. In addition, a negative control group (SB), which not only maintains self-renewal but also has similar components with R2i, helped us to develop an appropriate strategy for detecting regulatory genes that play a key role in ESC derivation.

This analysis has identified a group of significantly expressed TFs and epigenetic modifiers in the ICM, different IOs, and ESCs. Our result was consistent with previously reported ICM-specific genes such as *Pramel4*, *Pramel5*, and *Pramel7* (Tang et al., 2010). In a systemic data analysis, we observed approximately 3,000 DEGs between the ICM and day 0.5 (Figure 2A). Instead of a fluctuated gene expression stemming from the *in vivo* to *in vitro* transition, we observed that these large numbers of DEGs could establish the path toward the generation of ESCs. We demonstrated that only 2 days were needed for deriving ESCs at maximal efficiency under R2i conditions (Figure 2C). However, we have suggested that ICM cells do not suddenly assume an ESC identity; the longer the cultivation period, the more likely it is that the cells will acquire ESC characteristics. We observed that, while the number of DEGs decreased between the late-stage IOs and established ESCs, ESCs showed downregulation of the EMT biological process compared with these IOs (Figure 2E). Despite the expression of common pluripotency factors, SB-treated IOs exited from pluripotency likely because they followed the EMT program. Our results are consistent with this notion, and have suggested that the ectopic expression of *Snail*, an EMT inducer, could impede the establishment of ESCs (Figure 6B). Normally, the corresponding protein presents quickly after the onset of gastrulation (Hemavathy et al., 1997). However, *Snail* surprisingly presents in SB-treated IOs as a downstream protein of the TGF-β signaling pathway. This data has illustrated that inhibition of TGF-β without

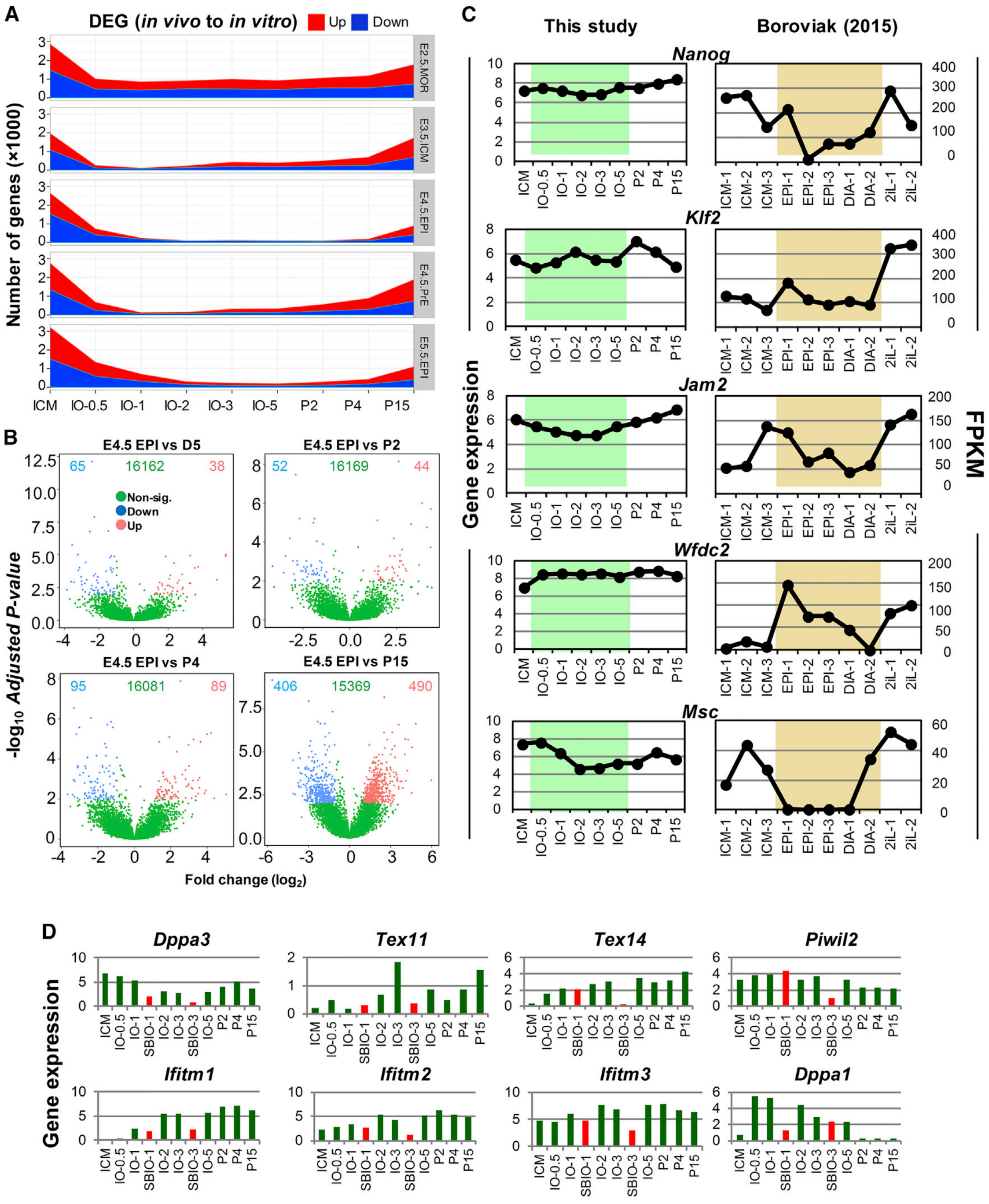

Figure 7. R2i-Treated IOs Exhibited preEpi Cell Characteristics

(A) DEGs between *in vivo* embryonic samples (Boroviak et al., 2015) and ICM to ESC time course samples for this study ($\geq$2-fold expression change, adjusted p $\leq$ 0.01).

(B) Volcano plots show fold change in gene expression (x axis, $\log_2$ fold change, adjusted p $\leq$ 0.01) between E4.5 preEpi cells (Boroviak et al., 2015), IO-5, and ESCs of different passages (this study). The y axis is the negative $\log_{10}$ of Benjamini-Hochberg adjusted p value. Blue, green, and red dots represent downregulated, non-significant, and upregulated genes, respectively.

(C) The comparability of the gene expression for some heatmap II-specific genes (from Figure 3) between *in vitro* IOs (this study) and single-preEpi cells (EPI1-3) or single-diapaused cells (DIA1-2; Boroviak et al., 2015).

(D) The expression of PGC-related genes in R2i- and SB-treated IOs.

suppression of FGF signaling could not block EMT and allow embryonic cells to exit from pluripotency. It has been reported that MEK suppression activates Klf4 through a phosphorylation pathway (Kim et al., 2012) and consequently induces MET (Chen et al., 2011). Accordingly, we observed that forced expression of *Klf4* in SB-treated IOs led to the generation of ESCs (Figure 6D). We also detected the downregulation of epithelial markers (*Cdh1* and *Klf4*) and the upregulation of mesenchymal markers (*Snail*, *Eomes*, and *Dab2*) under conventional conditions, i.e., serum + LIF (Figure 6A). Therefore, we concluded that the lack of EMT blockage was the main reason for the inefficient generation of ESCs from a refractory cell strain under serum or SB culture conditions. When EMT blockage occurs, SB or serum could support the long-term maintenance of ESC self-renewal.

We believe that the strategies developed in this study can help identify the most important gene regulators for deriving pluripotent ESCs. Based on significant changes in gene expression patterns and morphological changes between treatment (R2i) and the control (SB) groups on day 3 (Figure 4), we postulated that DEGs between these two groups was crucial for ICM to ESC transition. The data showed that pluripotency factors such as *Pou5f1*, *Zfp42*, *Klf4*, *Sall4*, *Ctnnb1*, and *Klf6* were not significantly altered between the treatment and control groups up to the third day of ESC derivation. We classified these 233 pluripotency markers as common pluripotency factors. However, the pluripotency markers that were significantly upregulated in R2i compared with SB, including *Nanog*, *Sox2*, *Klf2*, *Esrrb*, *Gbx2*, *Jam2*, *Dppa3*, *Msc*, and *Wfdc2*, could be the genes driving the establishment of ESCs (Figure 3C). Of all the TFs that play an essential role in maintaining pluripotency (Dunn et al., 2014), half were included in the R2i-specific gene list (Figure S3B). Therefore, we proposed that the overexpression of these genes, such as *Nanog*, would undermine the necessity of a special regimen, such as R2i and 2i (Figure 3D).

In this study, we also found a dramatic difference in the DNA methylation patterns between ICM cells and ESCs. The passive and active demethylation of maternal and paternal genomes resulted in a hypomethylated epigenetic landscape for ICM cells (Liao et al., 2015). It has been shown that ESCs have higher DNA methylation levels than ICM cells under conventional conditions, as opposed to 2i conditions (Leitch et al., 2013). DNA methylation and DNA methyltransferases expression have been broadly implicated in early embryo development and long-term maintenance of pluripotent cells (Arand et al., 2012; Ficz et al., 2013; Habibi et al., 2013; Marks et al., 2012; Smith et al., 2012), but the exact epigenetic events that occur during the derivation of ESCs have remained elusive (Tang et al., 2010). Here, we have observed that the expression of epigenetic modifier genes, such as *Dnmt3b*, *Dnmt3l*, *Chd8*, *Mtss1*, *Suz12*, *Eed*, *Wdr3*, and *Mat2b*, was significantly increased in the intermediate stages of ESC derivation, and followed an upward trend during the course of the culture (Figure 4C). Next, we measured the level of DNA methylation in the CpG dyads of IAP-LTR1 and L1Md_tf retrotransposable elements and mSat. Consistent with the results of previous studies (Ficz et al., 2013; Habibi et al., 2013; Smith et al., 2012), this entire dataset has highlighted the role of epigenetic modifiers and DNA methylation, pointing toward a reconstruction process that shapes a different epigenetic landscape for ESCs. In contrast with previous observations (Ficz et al., 2013; Okashita et al., 2014), blockage of the ERK pathway was probably required for a high expression of epigenetic modifiers and DNA methylation-related genes during ICM to ESC transition. Interestingly, we found that RG108-mediated inhibition of DNA methylation impeded the efficient derivation of ESCs in the presence of R2i, yet had no adverse effects under 2i conditions. This analysis highlighted a major difference between the R2i and 2i conditions in the regulation of pluripotency. It has previously been demonstrated that the augmented BMP4 signaling pathway plays a key role in R2i pluripotency, whereas it does not make a difference in the 2i condition (Gomes Fernandes et al., 2016; Hassani et al., 2014b). Despite the high degree of similarity between the R2i and 2i conditions, as well as the strength of R2i in supporting pluripotency, i.e., in establishing and maintaining ESCs from single blastomeres (Hassani et al., 2014a) and embryonic germ cells from the PGCs of mice and rats (Attari et al., 2014; Mohammadi et al., 2015), these two conditions work in different ways.

To determine the state of R2i pluripotent cells in comparison with *in vivo* pluripotent cells, we compared our transcriptome data with that of early embryonic cells (Figure 7). Although a considerable number of preEpi- and PE-specific markers demonstrated concurrent expression patterns in both the R2i- and SB-related clusters, the enrichment of substantial preEpi- and PE-specific genes in the R2i and SB groups, would suggest a specified path for the generation of ESCs from ICM cells. We observed that R2i treatment could support the expression of diapause- and PGC-related genes, which were associated with the preEpi cell state. A comparison of 2i-grown cells with early embryonic cells indicated that there was greater similarity with them and E4.5 preEpi cells (Boroviak et al., 2015). In this study, we also demonstrated that R2i could sustain the preEpi-specific gene expression during the process of establishing ESCs from the ICM. IOs and early-passage ESCs were more similar to preEpi cells than were P15 ESCs. This was consistent with data by Boroviak et al. Therefore, despite certain differences between 2i and R2i for maintaining pluripotency, both conditions produced ESCs that

exhibited high similarity to E4.5 preEpi cells. However, our results demonstrated that the establishment of ESCs requires a reprogramming phenomenon such as DNA methylation regulation and EMT blockage.

To the best of our knowledge, this is the first study reporting that EMT prevention is required for the derivation of ESCs from the ICM. In addition to the maintenance of preEpi-specific marker gene expression, prevention of TGF-β and MAPK under R2i conditions tended to inhibit the EMT process in normal development of the early embryo and to maintain pluripotency *in vitro*. From the perspective of human ESCs, it would be of tremendous interest to investigate the role of EMT or cell adhesion in the derivation of primed and naive human ESCs.

EXPERIMENTAL PROCEDURES

ESC Derivation and Sample Collection

For ESC derivation, blastocysts/isolated ICMs were prepared as described in the Supplemental Experimental Procedures. The isolated ICMs were washed twice in PBS, and then selected for microarray analysis. ESCs were derived by transferring blastocysts/isolated ICMs on gelatin-coated plates (0.1%, Sigma-Aldrich) that contained N2B27 defined medium supplemented with R2i (which included 1 μM PD0325901 [Stemgent] and 10 μM SB431542 [Sigma-Aldrich]) and 1,000 U/mL LIF (ESGRO, Millipore). This time was designated as day 0. For the first microarray analysis, the samples included isolated ICMs (day 0), BOs on days 3, 5, 7, and 9 (BO3–9) after plating, and ESCs on passage 20, in three biological replicates. We chose and pooled approximately 20–30 isolated ICMs and BOs for each biological replicate.

For the second microarray analysis, we collected new samples in a time resolution experiment that included immunosurgically isolated ICM, IOs, on days 0.5, 1, 2, 3, and 5 (IO0.5–5), and passages 2, 4, and 15 of ESCs (P2–15). In addition, IOs which were cultivated in N2B27 supplemented with SB431542 + LIF on days 1 (SBIO-1) and 3 (SBIO-3) considered as the negative controls. Approximately 30–40 ICM or IOs were picked and pooled in two biological replicates.

ACCESSION NUMBERS

Gene profiling data was submitted to the GEO repository with accession number GEO: GSE87793.

SUPPLEMENTAL INFORMATION

Supplemental Information includes Supplemental Experimental Procedures, six figures, and four tables and can be found with this article online at http://dx.doi.org/10.1016/j.stemcr.2017.08.006.

AUTHOR CONTRIBUTIONS

M.T., S.N.-H., and A.S. developed the study. N.T. performed some of the EMT experiments and discussed the EMT results. K.A. discussed the EMT results. J.A. and J.W. performed the DNA methylation experiments. B.G. discussed the gene expression results. D.S. assisted with animal and embryo manipulations. M.A. analyzed the microarray data. M.P. performed the time course derivation and supplemental experiments. H.G. supervised the project, provided financial support, and discussed the results. H.S. designed the experiments, discussed the results, and approved the manuscript. H.B. provided financial support, designed and analyzed experiments, discussed the results, and approved the manuscript.

ACKNOWLEDGMENTS

We are grateful to Martina Sinn and Azam Samadian for qRT-PCR, Mahdi Hesaraki for viral production, Razieh Karamzadeh for discussion, Areti Malapetsas for final proofreading, Adeleh Taei for discussion, and Behrouz Asgari for assisting with animal and blastocyst manipulation. M.J.A.-B. was supported for this work by grants from the Ministry of Economy and Competitivity, Spain, MINECO grant BFU 2016-7798-P, and DFG10/15, DFG15/15, and DFG141/16 from Diputación Foral de Gipuzkoa, Spain. This work was also supported by the Royan Institute, the Iranian Council of Stem Cell Research and Technology, the Iran National Science Foundation (INSF), and the Iran Science Elites Federation (to H.B.).

Received: January 28, 2017
Revised: August 12, 2017
Accepted: August 14, 2017
Published: September 14, 2017

REFERENCES

Arand, J., Spieler, D., Karius, T., Branco, M.R., Meilinger, D., Meissner, A., Jenuwein, T., Xu, G., Leonhardt, H., Wolf, V., et al. (2012). In vivo control of CpG and non-CpG DNA methylation by DNA methyltransferases. PLoS Genet. *8*, e1002750.

Attari, F., Sepehri, H., Ansari, H., Hassani, S.N., Esfandiari, F., Asgari, B., Shahverdi, A., and Baharvand, H. (2014). Efficient induction of pluripotency in primordial germ cells by dual inhibition of TGF-beta and ERK signaling pathways. Stem Cells Dev. *23*, 1050–1061.

Batlle-Morera, L., Smith, A., and Nichols, J. (2008). Parameters influencing derivation of embryonic stem cells from murine embryos. Genesis *46*, 758–767.

Boroviak, T., Loos, R., Lombard, P., Okahara, J., Behr, R., Sasaki, E., Nichols, J., Smith, A., and Bertone, P. (2015). Lineage-specific profiling delineates the emergence and progression of naive pluripotency in mammalian embryogenesis. Dev. Cell *35*, 366–382.

Brook, F.A., and Gardner, R.L. (1997). The origin and efficient derivation of embryonic stem cells in the mouse. Proc. Natl. Acad. Sci. USA *94*, 5709–5712.

Chen, J., Liu, J., Yang, J., Chen, Y., Ni, S., Song, H., Zeng, L., Ding, K., and Pei, D. (2011). BMPs functionally replace Klf4 and support efficient reprogramming of mouse fibroblasts by Oct4 alone. Cell Res. *21*, 205–212.

Dunn, S.J., Martello, G., Yordanov, B., Emmott, S., and Smith, A.G. (2014). Defining an essential transcription factor program for naive pluripotency. Science *344*, 1156–1160.

Evans, M.J., and Kaufman, M.H. (1981). Establishment in culture of pluripotential cells from mouse embryos. Nature *292*, 154–156.

Ficz, G., Hore, T.A., Santos, F., Lee, H.J., Dean, W., Arand, J., Krueger, F., Oxley, D., Paul, Y.L., Walter, J., et al. (2013). FGF signaling inhibition in ESCs drives rapid genome-wide demethylation to the epigenetic ground state of pluripotency. Cell Stem Cell *13*, 351–359.

Gerovska, D., and Arauzo-Bravo, M.J. (2016). Does mouse embryo primordial germ cell activation start before implantation as suggested by single-cell transcriptomics dynamics? Mol. Hum. Reprod. *22*, 208–225.

Gomes Fernandes, M., Dries, R., Roost, M.S., Semrau, S., de Melo Bernardo, A., Davis, R.P., Ramakrishnan, R., Szuhai, K., Maas, E., Umans, L., et al. (2016). BMP-SMAD signaling regulates lineage priming, but is dispensable for self-renewal in mouse embryonic stem cells. Stem Cell Reports *6*, 85–94.

Habibi, E., Brinkman, A.B., Arand, J., Kroeze, L.I., Kerstens, H.H., Matarese, F., Lepikhov, K., Gut, M., Brun-Heath, I., Hubner, N.C., et al. (2013). Whole-genome bisulfite sequencing of two distinct interconvertible DNA methylomes of mouse embryonic stem cells. Cell Stem Cell *13*, 360–369.

Hassani, S.N., Totonchi, M., Farrokhi, A., Taei, A., Larijani, M.R., Gourabi, H., and Baharvand, H. (2012). Simultaneous suppression of TGF-beta and ERK signaling contributes to the highly efficient and reproducible generation of mouse embryonic stem cells from previously considered refractory and non-permissive strains. Stem Cell Rev. *8*, 472–481.

Hassani, S.N., Pakzad, M., Asgari, B., Taei, A., and Baharvand, H. (2014a). Suppression of transforming growth factor beta signaling promotes ground state pluripotency from single blastomeres. Hum. Reprod. *29*, 1739–1748.

Hassani, S.N., Totonchi, M., Sharifi-Zarchi, A., Mollamohammadi, S., Pakzad, M., Moradi, S., Samadian, A., Masoudi, N., Mirshahvaladi, S., Farrokhi, A., et al. (2014b). Inhibition of TGFbeta signaling promotes ground state pluripotency. Stem Cell Rev. *10*, 16–30.

Hemavathy, K., Meng, X., and Ip, Y.T. (1997). Differential regulation of gastrulation and neuroectodermal gene expression by Snail in the Drosophila embryo. Development *124*, 3683–3691.

Kim, M.O., Kim, S.H., Cho, Y.Y., Nadas, J., Jeong, C.H., Yao, K., Kim, D.J., Yu, D.H., Keum, Y.S., Lee, K.Y., et al. (2012). ERK1 and ERK2 regulate embryonic stem cell self-renewal through phosphorylation of Klf4. Nat. Struct. Mol. Biol. *19*, 283–290.

Leitch, H.G., McEwen, K.R., Turp, A., Encheva, V., Carroll, T., Grabole, N., Mansfield, W., Nashun, B., Knezovich, J.G., Smith, A., et al. (2013). Naive pluripotency is associated with global DNA hypomethylation. Nat. Struct. Mol. Biol. *20*, 311–316.

Liao, J., Karnik, R., Gu, H., Ziller, M.J., Clement, K., Tsankov, A.M., Akopian, V., Gifford, C.A., Donaghey, J., Galonska, C., et al. (2015). Targeted disruption of DNMT1, DNMT3A and DNMT3B in human embryonic stem cells. Nat. Genet. *47*, 469–478.

Loh, K.M., Lim, B., and Ang, L.T. (2015). Ex uno plures: molecular designs for embryonic pluripotency. Physiol. Rev. *95*, 245–295.

Marks, H., Kalkan, T., Menafra, R., Denissov, S., Jones, K., Hofemeister, H., Nichols, J., Kranz, A., Stewart, A.F., Smith, A., et al. (2012). The transcriptional and epigenomic foundations of ground state pluripotency. Cell *149*, 590–604.

Mohammadi, A., Attari, F., Babapour, V., Hassani, S.N., Masoudi, N., Shahverdi, A., and Baharvand, H. (2015). Generation of rat embryonic germ cells via inhibition of TGFbeta and MEK pathways. Cell J. *17*, 288–295.

Mullen, A.C., Orlando, D.A., Newman, J.J., Loven, J., Kumar, R.M., Bilodeau, S., Reddy, J., Guenther, M.G., DeKoter, R.P., and Young, R.A. (2011). Master transcription factors determine cell-type-specific responses to TGF-beta signaling. Cell *147*, 565–576.

Nichols, J., and Smith, A. (2011). The origin and identity of embryonic stem cells. Development *138*, 3–8.

Okashita, N., Kumaki, Y., Ebi, K., Nishi, M., Okamoto, Y., Nakayama, M., Hashimoto, S., Nakamura, T., Sugasawa, K., Kojima, N., et al. (2014). PRDM14 promotes active DNA demethylation through the ten-eleven translocation (TET)-mediated base excision repair pathway in embryonic stem cells. Development *141*, 269–280.

Smith, Z.D., Chan, M.M., Mikkelsen, T.S., Gu, H., Gnirke, A., Regev, A., and Meissner, A. (2012). A unique regulatory phase of DNA methylation in the early mammalian embryo. Nature *484*, 339–344.

Tang, F., Barbacioru, C., Bao, S., Lee, C., Nordman, E., Wang, X., Lao, K., and Surani, M.A. (2010). Tracing the derivation of embryonic stem cells from the inner cell mass by single-cell RNA-seq analysis. Cell Stem Cell *6*, 468–478.

Ying, Q.L., Wray, J., Nichols, J., Batlle-Morera, L., Doble, B., Woodgett, J., Cohen, P., and Smith, A. (2008). The ground state of embryonic stem cell self-renewal. Nature *453*, 519–523.

Stem Cell Reports, Volume *9*

Supplemental Information

Blockage of the Epithelial-to-Mesenchymal Transition Is Required for Embryonic Stem Cell Derivation

Mehdi Totonchi, Seyedeh-Nafiseh Hassani, Ali Sharifi-Zarchi, Natalia Tapia, Kenjiro Adachi, Julia Arand, Boris Greber, Davood Sabour, Marcos J. Araúzo-Bravo, Jörn Walter, Mohammad Pakzad, Hamid Gourabi, Hans R. Schöler, and Hossein Baharvand

Supplemental Information

Blockage of the Epithelial-to-Mesenchymal Transition Is Required for Embryonic Stem Cell Derivation

Mehdi Totonchi, Seyedeh-Nafiseh Hassani, Ali Sharifi-Zarchi, Natalia Tapia, Kenjiro Adachi, Julia Arand, Boris Greber, Davood Sabour, Marcos J. Araúzo-Bravo, Jörn Walter, Mohammad Pakzad, Hamid Gourabi, Hans R. Schöler, Hossein Baharvand

INVENTORY OF SUPPLEMENTARY INFORMATION

I. Supplementary Data

Figure S1, related Figure 1.

Figure S2, related Figure 2.

Figure S3, related Figure 3.

Figure S4, related Figure 5.

Figure S5, related Figure 6.

Figure S6, related Figure 7

Table S1, related Figure 3

Table S2, related Figure 3

Table S3, related Figure 5

Table S4, related Figure 7

II. Supplemental Experimental Procedure

III. Supplemental References

Supplemental Figures

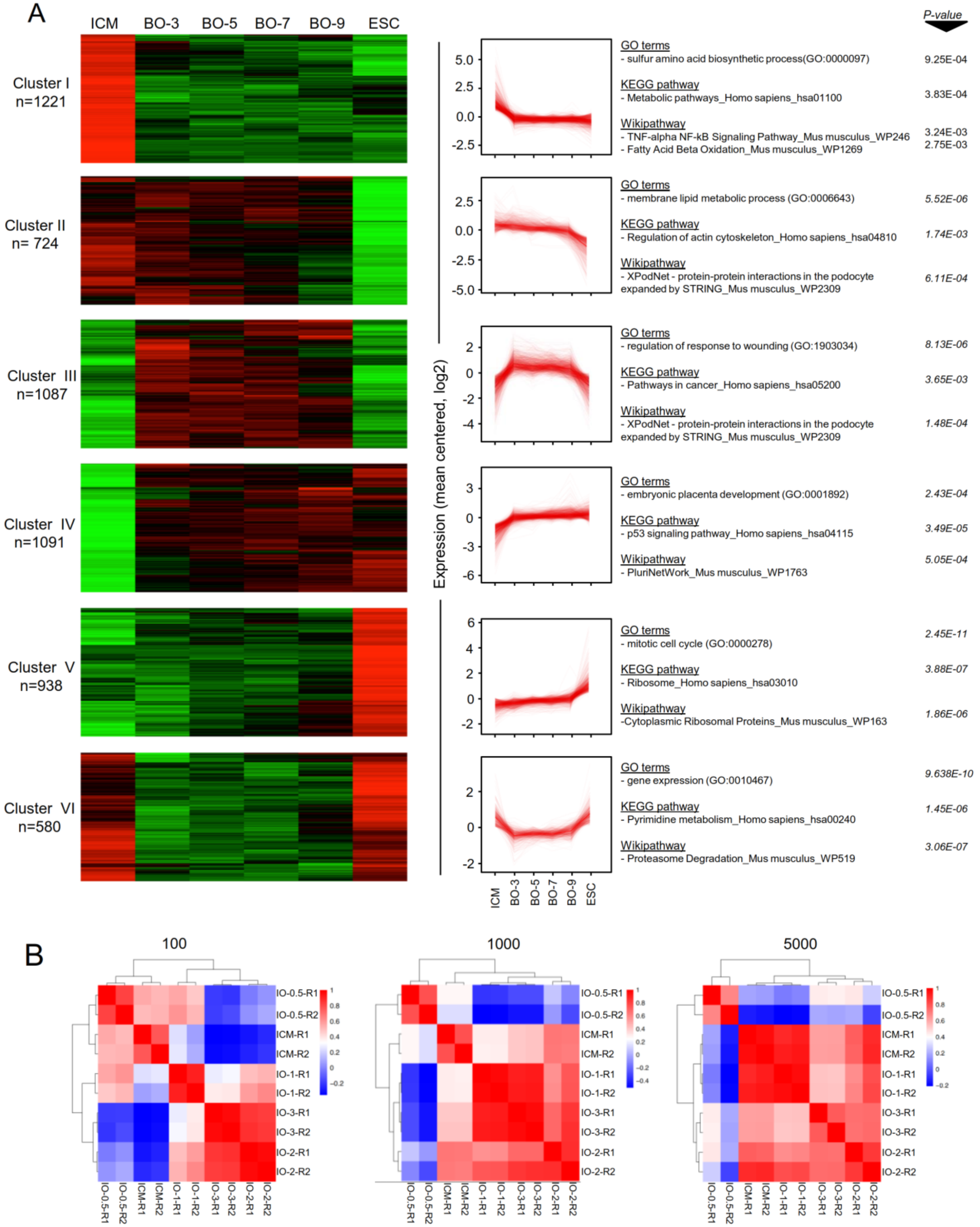

Figure S1. Temporal morphology and time-course transcriptome profiling of ESC derivation, related to Figure 1.

(A) Unsupervised hierarchical clustering and functional annotation of time-course gene expression profiles for ESC derivation. We used three independent biological replicates, except for BO-3, which consisted of two biological replicates. Between 20 and 30 ICMs were used for each replicate. Transcript levels are mean-centered log2 scaled values.

(B) Spearman correlation heatmap analysis among the first timepoints (ICM, IO-0.5, IO-1, IO-2 and IO-3) to measure the differences among replicates vs. timepoints. Since the expression levels of a minority of the genes have significant alternations among samples, this analysis was limited to 100, 1000 and 5000 microarray probes that had the maximum expression variance among these samples. The biological replicates are clustered together in this analysis, which showed the differences among consecutive samples are larger than the observed fluctuation among replicates. This shows that the alternations among consecutive samples are biologically relevant.

vs ICM

A

Up-regulated

IO-0.5

GO Term	P-val.
mitotic cell cycle (GO:0000278)	5.76E-06
**KEGG pathway**	**P-val.**
Ubiquitin mediated proteolysis_Homo sapiens_hsa04120	5.98E-04
Adrenergic signaling in cardiomyocytes_Homo sapiens_hsa04261	3.09E-03
TNF signaling pathway_Homo sapiens_hsa04668	4.13E-03
Glutathione metabolism_Homo sapiens_hsa00480	4.05E-03
Hepatitis C_Homo sapiens_hsa05160	4.82E-03
Cell cycle_Homo sapiens_hsa04110	5.41E-03
Small cell lung cancer_Homo sapiens_hsa05222	7.03E-03

IO-1

GO Term	P-val.
mitotic cell cycle (GO:0000278)	2.95E-06
Ras protein signal transduction (GO:0007265)	4.56E-06
**KEGG pathway**	**P-val.**
Cysteine and methionine metabolism_Homo sapiens_hsa00270	9.34E-04
Small cell lung cancer_Homo sapiens_hsa05222	1.00E-03

IO-2

GO Term	P-val.
mitotic cell cycle (GO:0000278)	6.41E-09
Ras protein signal transduction (GO:0007265)	4.44E-07
**KEGG pathway**	**P-val.**
Small cell lung cancer_Homo sapiens_hsa05222	8.99E-05

IO-3

GO Term	P-val.
mitotic cell cycle (GO:0000278)	6.99E-09
Ras protein signal transduction (GO:0007265)	1.50E-06
negative regulation of cell cycle (GO:0045786)	1.24E-05
**KEGG pathway**	**P-val.**
Small cell lung cancer_Homo sapiens_hsa05222	6.49E-06
Pancreatic cancer_Homo sapiens_hsa05212	1.77E-05

IO-5

GO Term	P-val.
mitotic cell cycle (GO:0000278)	1.58E-09
negative regulation of cell cycle (GO:0045786)	1.60E-06
response to ionizing radiation (GO:0010212)	2.89E-06
Ras protein signal transduction (GO:0007265)	3.43E-06
response to radiation (GO:0009314)	1.19E-05
transforming growth factor beta receptor signaling pathway (GO:0007179)	7.50E-06
**KEGG pathway**	**P-val.**
p53 signaling pathway_Homo sapiens_hsa04115	1.63E-05
Cell cycle_Homo sapiens_hsa04110	1.36E-05
FoxO signaling pathway_Homo sapiens_hsa04068	4.27E-05
Pancreatic cancer_Homo sapiens_hsa05212	7.69E-05
Ubiquitin mediated proteolysis_Homo sapiens_hsa04120	3.03E-05
Small cell lung cancer_Homo sapiens_hsa05222	9.55E-05

P2

GO Term	P-val.
mitotic cell cycle (GO:0000278)	7.57E-11
**KEGG pathway**	**P-val.**
Ubiquitin mediated proteolysis_Homo sapiens_hsa04120	2.74E-06
Neurotrophin signaling pathway_Homo sapiens_hsa04722	9.22E-05
Small cell lung cancer_Homo sapiens_hsa05222	1.38E-04

Down-regulated

IO-0.5

GO Term	P-val.
membrane lipid catabolic process (GO:0046466)	1.26E-04
glycolipid catabolic process (GO:0019377)	1.93E-04
**KEGG pathway**	**P-val.**
Other glycan degradation_Homo sapiens_hsa00511	2.89E-04
Glutamatergic synapse_Homo sapiens_hsa04724	4.53E-03
Glycine, serine and threonine metabolism_Homo sapiens_hsa00260	4.17E-03
Protein digestion and absorption_Homo sapiens_hsa04974	3.97E-03
Metabolic pathways_Homo sapiens_hsa01100	8.26E-03

IO-1

GO Term	P-val.
hexose biosynthetic process (GO:0019319)	3.69E-05
regulation of uterine smooth muscle contraction (GO:0070472)	1.48E-04
monosaccharide biosynthetic process (GO:0046364)	9.99E-05
regulation of blood circulation (GO:1903522)	2.31E-04
response to isoquinoline alkaloid (GO:0014072)	4.58E-04
membrane lipid catabolic process (GO:0046466)	1.09E-03
**KEGG pathway**	**P-val.**
Glycine, serine and threonine metabolism_Homo sapiens_hsa00260	1.82E-03
Glutamatergic synapse_Homo sapiens_hsa04724	3.51E-03
Other glycan degradation_Homo sapiens_hsa00511	2.07E-03
Protein digestion and absorption_Homo sapiens_hsa04974	2.7E-03
Metabolic pathways_Homo sapiens_hsa01100	6.91E-03

IO-2

GO Term	P-val.
cellular amino acid metabolic process (GO:0006520)	3.22E-05
membrane lipid catabolic process (GO:0046466)	1.06E-03
glycolipid catabolic process (GO:0019377)	1.05E-03
**KEGG pathway**	**P-val.**
Metabolic pathways_Homo sapiens_hsa01100	6.97E-03
Sphingolipid metabolism_Homo sapiens_hsa00600	9.62E-03
Other glycan degradation_Homo sapiens_hsa00511	1.97E-03

IO-3

GO Term	P-val.
pyrimidine nucleobase metabolic process (GO:0006206)	1.52E-03
cellular amino acid metabolic process (GO:0006520)	7.45E-04
cellular component disassembly (GO:0022411)	4.65E-04
T cell proliferation (GO:0042098)	8.61E-04
protein targeting (GO:0006605)	1.48E-03
single-organism carbohydrate catabolic process (GO:0044724)	1.15E-03
hexose biosynthetic process (GO:0019319)	1.34E-03
cellular amino acid biosynthetic process (GO:0008652)	5.55E-04
glial cell differentiation (GO:0010001)	9.61E-04
mononuclear cell proliferation (GO:0032943)	1.11E-03
lymphocyte proliferation (GO:0046651)	8.72E-04
membrane lipid catabolic process (GO:0046466)	2.58E-03
**KEGG pathway**	**P-val.**
Metabolic pathways_Homo sapiens_hsa01100	1.22E-04
Amino sugar and nucleotide sugar metabolism_Homo sapiens_hsa00520	4.85E-03
Other glycan degradation_Homo sapiens_hsa00511	4.36E-03

IO-5

GO Term	P-val.
cellular component disassembly (GO:0022411)	7.94E-05
hexose biosynthetic process (GO:0019319)	1.39E-04
protein targeting (GO:0006605)	2.52E-04
pyrimidine nucleobase metabolic process (GO:0006206)	6.05E-04
monosaccharide biosynthetic process (GO:0046364)	3.75E-04
single-organism carbohydrate catabolic process (GO:0044724)	5.41E-04
membrane lipid catabolic process (GO:0046466)	1.2E-03
**KEGG pathway**	**P-val.**
Metabolic pathways_Homo sapiens_hsa01100	9.19E-06
Ribosome_Homo sapiens_hsa03010	6.52E-04
Glycine, serine and threonine metabolism_Homo sapiens_hsa00260	1.5E-03
Other glycan degradation_Homo sapiens_hsa00511	2.26E-03

P2

GO Term	P-val.
glycolipid catabolic process (GO:0019377)	2.88E-04
membrane lipid catabolic process (GO:0046466)	4.07E-04
**KEGG pathway**	**P-val.**
Metabolic pathways_Homo sapiens_hsa01100	2.0E-04
Glycine, serine and threonine metabolism_Homo sapiens_hsa00260	2.12E-03
Glycerophospholipid metabolism_Homo sapiens_hsa00564	1.84E-03
Protein digestion and absorption_Homo sapiens_hsa04974	2.4E-03
Other glycan degradation_Homo sapiens_hsa00511	2.89E-03

B

P2 vs

Up-regulated

	GO term	*P-val.*
ICM	mitotic cell cycle	1.98E-10
IO-0.5	regulation of protein homooligomerization	3.41E-05
IO-1	renal tubule morphogenesis	5.42E-04
	regulation of coenzyme metabolic process	4.35E-04
IO-2	positive regulation of acute inflammatory response	9.14E-04
	intrinsic apoptotic signaling pathway in response to endoplasmic reticulum stress	8.49E-04
IO-3	intrinsic apoptotic signaling pathway in response to endoplasmic reticulum stress	2.12E-04
	regulation of gene expression, epigenetic	1.87E-04

Down-regulated

	GO term	*P-val.*
ICM	cellular amino acid metabolic process	5.41E-05
IO-0.5	lipid biosynthetic process	2.01E-06
	phospholipid metabolic process	2.51E-06
IO-1	organic acid transport	1.13E-06
	carboxylic acid transport	9.94E-07
	extracellular matrix organization	3.21E-06
IO-2	epithelial cell differentiation	2.18E-06
	organic acid transport	2.27E-06
	carboxylic acid transport	2.06E-06
	smooth muscle cell differentiation	3.19E-05
IO-3	epithelial cell differentiation	6.75E-08
	smooth muscle cell differentiation	3E-05
	definitive hemopoiesis	3.58E-05
	organ formation	6.83E-05
IO-5	organ formation	4.99E-06
	epithelial cell differentiation	1.27E-06
	smooth muscle cell differentiation	3.25E-05
	Angiogenesis	8.62E-06
	regulation of epithelial cell migration	1.05E-05

C

P4 vs

Up-regulated

	GO term	*P-val.*
ICM	mitotic cell cycle	3.05E-13
	negative regulation of cell cycle	4.01E-08
	cell cycle phase transition	1.47E-08
	mitotic cell cycle phase transition	4.24E-08
IO-0.5	regulation of response to DNA damage stimulus	6.78E-08
	regulation of cellular response to stress	4.42E-07
	DNA repair	3.76E-06
	regulation of DNA metabolic process	4.18E-06
IO-1	intraciliary transport	2.96E-04
	positive regulation of histone methylation	1.89E-03
	regulation of cellular response to stress	3.96E-04
	negative regulation of cell cycle	2.02E-03
IO-2	endosome organization	1.74E-03
IO-3	male gonad development	1.85E-05
	reproductive structure development	6.82E-05
	gonad development	7.68E-05

Down-regulated

	GO term	*P-val.*
ICM	cellular amino acid metabolic process	2.62E-05
	membrane lipid catabolic process	3.5E-04
IO-0.5	mannosylation	2.1E-04
	glutathione derivative metabolic process	6.99E-05
IO-1	mannosylation	7.11E-06
	endocytosis	1.6E-05
	glutathione derivative metabolic process	7.68E-05
	glutathione derivative biosynthetic process	7.68E-05
	post-translational protein modification	1.89E-05
	Glycosylation	6.55E-05
IO-2	extracellular matrix organization	5.42E-06
	extracellular structure organization	5.69E-06
	protein N-linked glycosylation via asparagine	2.12E-05
	peptidyl-asparagine modification	2.34E-05
	protein N-linked glycosylation	4.52E-05
IO-3	extracellular matrix organization	1.72E-08
	extracellular structure organization	1.79E-08
	regulation of epithelial cell migration	2.1E-06
	epithelial cell differentiation	6.59E-06
	smooth muscle cell differentiation	2.65E-05
	tissue morphogenesis	9.43E-06
IO-5	extracellular matrix organization	1.5E-07
	extracellular structure organization	1.54E-07
	response to decreased oxygen levels	2.96E-06
	response to hypoxia	2.6E-06
	response to oxygen levels	4.59E-06
	trabecula formation	8.71E-06
	epithelial cell differentiation	2.03E-06
	regulation of epithelial cell migration	3.55E-06

D

P15 vs

Up-regulated

	GO term	*P-val.*
ICM	mitotic cell cycle	4.99E-11
	negative regulation of cell cycle	3.44E-07
	organelle fission	1.96E-07
	nuclear division	1.2E-07
	proteolysis involved in cellular protein catabolic process	7.58E-07
	modification-dependent macromolecule catabolic process	8.07E-07
	modification-dependent protein catabolic process	9.66E-07
	cell cycle phase transition	7.01E-07
IO-0.5	regulation of cellular response to stress	1.12E-06
	DNA repair	1.03E-06
	regulation of DNA metabolic process	6.44E-07
	mitotic cell cycle	5.78E-07
	chromatin modification	4.07E-06
	regulation of response to DNA damage stimulus	4.57E-06
	organelle fission	1.32E-05
	nuclear division	1.18E-05
	cell cycle phase transition	2.12E-05
IO-1	DNA repair	1.27E-07
	mitotic cell cycle	2.86E-07
	organelle fission	1.00E-06
	nuclear division	2.35E-06
	chromatin modification	4.74E-06
	cell cycle checkpoint	5.84E-06
	regulation of DNA metabolic process	1.53E-05
IO-2	organelle fission	5.22E-11
	nuclear division	8.28E-11
	mitotic cell cycle	4.62E-10
	mitotic nuclear division	3.31E-09
	chromatin modification	8.76E-07
	histone modification	5.8E-06
	covalent chromatin modification	7.15E-06
IO-3	positive regulation of macroautophagy	4.17E-04
	proteolysis involved in cellular protein catabolic process	2.74E-04
	modification-dependent macromolecule catabolic process	1.25E-04
	modification-dependent protein catabolic process	2.9E-04
	ubiquitin-dependent protein catabolic process	5.55E-04
	organelle fission	5.29E-04
	nuclear division	5.49E-04
	mitotic cell cycle	4.15E-04
IO-5	organelle fission	5.7E-04
	nuclear division	3.09E-04

Dow-regulated

	GO term	*P-val.*
ICM	translation	2.77E-08
	gene expression	4.57E-06
	protein localization to membrane	3.18E-06
	negative regulation of protein modification process	1.51E-05
	single-organism cellular localization	1.35E-05
	cellular component disassembly	2.12E-05
	protein targeting	2.75E-05
IO-0.5	translation	4.37E-14
	gene expression	4.64E-10
	translational initiation	4.70E-07
	electron transport chain	2.86E-07
	respiratory electron transport chain	5.13E-07
	protein targeting to ER	1.25E-06
	protein localization to endoplasmic reticulum	1.27E-06
IO-1	translation	2.25E-12
	gene expression	8.34E-10
	protein localization to endoplasmic reticulum	8.17E-07
	protein localization to membrane	2.33E-05
	translational initiation	1.24E-05
	protein targeting to ER	6.89E-06
	protein N-linked glycosylation via asparagine	1.92E-05
IO-2	translation	1.27E-08
	gene expression	3.69E-08
	negative regulation of transferase activity	9.38E-05
	protein N-linked glycosylation via asparagine	6.57E-05
	peptidyl-asparagine modification	7.44E-05
IO-3	gene expression	1.86E-05
	establishment or maintenance of epithelial cell apical/basal polarity	5.96E-04
	smooth muscle cell differentiation	8.39E-04
	epithelial cell differentiation	7.46E-05
IO-5	regulation of epithelial cell migration	3.63E-08
	smooth muscle cell differentiation	1.29E-05
	muscle cell differentiation	2.71E-05
	organ formation	2.53E-04
	positive regulation of locomotion	1.11E-04
	response to hypoxia	1.38E-04
	response to decreased oxygen levels	1.62E-04
	positive regulation of cell migration	1.64E-04
	tissue morphogenesis	6.06E-05
	positive regulation of epithelial cell migration	5.2E-05

E

P4 vs P2

Up-regulated

GO term	*P-val.*
endosome organization	2.01E-03

Dow-regulated

GO term	*P-val.*
regulation of translational elongation	8.15E-04

P15 vs P4

Up-regulated

GO term	*P-val.*
chromosome organization involved in meiosis	5.257E-05

Dow-regulated

GO term	*P-val.*
lysosome localization	4.67E-04

Figure S2. Functional annotation for up- and down-regulated genes during the transition from ICM cells to ESCs, related to Figure 2.

(A) Functional annotation for DEG in the ICM versus different IOs/ESCs. The pathways significantly enriched by the GO term and KEGG pathways are shown. The analysis has been performed by the Enrichr web tool.

(B-D) Functional annotation for DEG in ESCs of different passages (P) versus the ICM. Tables show the statistically significant enriched pathways for ESCs of P2 **(B)**, P4 **(C)**, and P15 **(D)** versus ICM/different IOs.

(E) Functional annotation of DEG between ESCs of different passages.

A

Heatmap I

GO Term	P-val.
regulation of fat cell differentiation (GO:0045598)	1.9E-04
glutathione metabolic process (GO:0006749)	2.46E-04
skeletal muscle cell differentiation (GO:0035914)	2.12E-04
**KEGG pathway**	**P-val.**
Glutathione metabolism_Homo sapiens_hsa00480	5.5E-04
**Wikipathway**	**P-val.**
Glutathione metabolism_Homo sapiens_WP100	5.5E-04

Heatmap II

GO Term	P-val.
anion transport (GO:0006820)	1.35E-04
**Wikipathway**	**P-val.**
Preimplantation Embryo_Homo sapiens_WP3527	3.52E-03

Heatmap III

GO Term	P-val.
chromatin modification (GO:0016568)	7.49E-06
DNA repair (GO:0006281)	2.49E-05
DNA catabolic process (GO:0006308)	1.92E-05
regulation of DNA metabolic process (GO:0051052)	1.38E-04
DNA-templated transcription, initiation (GO:0006352)	8.91E-05
mitotic cell cycle (GO:0000278)	1.41E-04
transcription initiation from RNA polymerase II promoter (GO:0006367)	1.03E-04
chromatin remodeling (GO:0006338)	8.18E-05
**KEGG pathway**	**P-val.**
Ribosome biogenesis in eukaryotes_Homo sapiens_hsa03008	1.44E-03
Melanoma_Homo sapiens_hsa05218	4.19E-03
RNA transport_Homo sapiens_hsa03013	7.72E-03
**Wikipathway**	**P-val.**
PluriNetWork_Mus musculus_WP1763	8.23E-06

Heatmap V

GO Term	P-val.
regulation of cofactor metabolic process (GO:0051193)	1.22E-04
**KEGG pathway**	**P-val.**
Signaling pathways regulating pluripotency of stem cells_Homo sapiens_hsa04550	2.08E-05
**Wikipathway**	**P-val.**
PluriNetWork_Mus musculus_WP1763	8.80E-04

Heatmap V

GO Term	P-val.
endoderm formation (GO:0001706)	2.18E-05
**Wikipathway**	**P-val.**
Endoderm Differentiation_Homo sapiens_WP2853	1.4E-04

Heatmap VI

GO Term	P-val.
lipid homeostasis (GO:0055088)	1.01E-05
plasma lipoprotein particle assembly (GO:0034377)	2.81E-04
sterol homeostasis (GO:0055092)	9.7E-05
protein destabilization (GO:0031648)	4.63E-04
cholesterol homeostasis (GO:0042632)	9.26E-05
**KEGG pathway**	**P-val.**
Rap1 signaling pathway_Homo sapiens_hsa04015	7.91E-03
**Wikipathway**	**P-val.**
Cardiac Progenitor Differentiation_Homo sapiens_WP2406	1.22E-03

Heatmap VII

GO Term	P-val.
response to topologically incorrect protein (GO:0035966)	3.69E-07
integrin-mediated signaling pathway (GO:0007229)	6.26E-07
response to unfolded protein (GO:0006986)	8.46E-07
regulation of cell morphogenesis (GO:0022604)	2.33E-05
cellular response to topologically incorrect protein (GO:0035967)	8.35E-06
angiogenesis (GO:0001525)	1.6E-05
ER-nucleus signaling pathway (GO:0006984)	1.02E-05
endoplasmic reticulum unfolded protein response (GO:0030968)	1.73E-05
cellular response to unfolded protein (GO:0034620)	2.34E-05
activation of signaling protein activity involved in unfolded protein response (GO:0006987)	6.69E-05
vasculogenesis (GO:0001570)	9.21E-05
positive regulation of nuclease activity (GO:0032075)	8.29E-05
**KEGG pathway**	**P-val.**
Lysosome_Homo sapiens_hsa04142	1.69E-07
Amino sugar and nucleotide sugar metabolism_Homo sapiens_hsa00520	4.17E-05
**Wikipathway**	**P-val.**
Fatty acid oxidation_Mus musculus_WP2318	4.1E-04
Glutathione metabolism_Mus musculus_WP164	2.11E-03
TGF-beta Signaling Pathway_Homo sapiens_WP366	1.54E-03

Heatmap VIII

GO Term	P-val.
hepatocyte apoptotic process (GO:0097284)	2.01E-04
calcium-independent cell-cell adhesion via plasma membrane cell-adhesion molecules (GO:0016338)	9.95E-04
**KEGG pathway**	**P-val.**
Alzheimer's disease_Homo sapiens_hsa05010	8.82E-03

Heatmaps I-V

GO terms	P-val.
negative regulation of cellular component organization (GO:0051129)	5.3E-04
regulation of DNA metabolic process (GO:0051052)	4.84E-04
chromatin modification (GO:0016568)	2.84E-04
pattern specification process (GO:0007389)	5.21E-04
regulation of collateral sprouting (GO:0048670)	6.85E-04
negative regulation of cell development (GO:0010721)	6.72E-04
negative regulation of cell-substrate adhesion (GO:0010812)	6.88E-04

KEGG pathway	P-val.
Signaling pathways regulating pluripotency of stem cells_Homo sapiens_hsa04550	5.68E-04
B cell receptor signaling pathway_Homo sapiens_hsa04662	5.33E-03
Melanoma_Homo sapiens_hsa05218	4.52E-03
Ribosome biogenesis in eukaryotes_Homo sapiens_hsa03008	6.13E-03
Dorso-ventral axis formation_Homo sapiens_hsa04320	8.3E-03

Wikipathway	P-val.
PluriNetWork_Mus musculus_WP1763	1.03E-09
Interactome of polycomb repressive complex 2 (PRC2) _Homo sapiens_WP2916	5.71E-04
Preimplantation Embryo_Homo sapiens_WP3527	2.12E-03

Heatmaps V-VIII

GO terms	P-val.
angiogenesis (GO:0001525)	1.53E-05
cell morphogenesis (GO:0000902)	1.91E-05
integrin-mediated signaling pathway (GO:0007229)	7.93E-06
response to topologically incorrect protein (GO:0035966)	8.42E-06
response to unfolded protein (GO:0006986)	1.6E-05
vasculogenesis (GO:0001570)	2.42E-05

KEGG pathway	P-val.
Lysosome_Homo sapiens_hsa04142	1.04E-06
Amino sugar and nucleotide sugar metabolism_Homo sapiens_hsa00520	2.19E-04
Protein processing in endoplasmic reticulum_Homo sapiens_hsa04141	1.35E-03

Wikipathway	P-val.
Focal Adhesion_Homo sapiens_WP306	1.97E-03
Focal Adhesion_Mus musculus_WP85	1.45E-03
Primary Focal Segmental Glomerulosclerosis FSGS_Homo sapiens_WP2572	1.75E-03
Fatty acid oxidation_Mus musculus_WP2318	1.04E-03

B

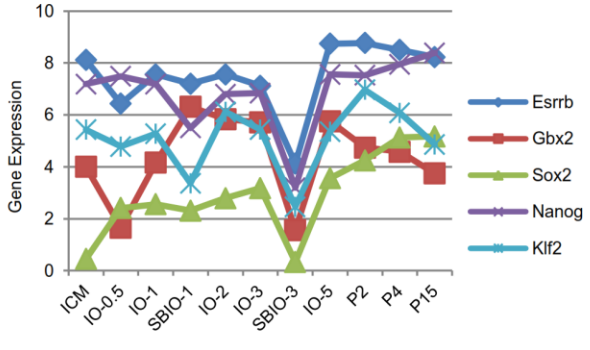

Figure S3. Transcriptome signature of R2i-treated IOs, related to Figure 3.

(A) Functional annotation for the represented heatmaps in Figure 3. Heatmaps I-IV show the up-regulated genes in R2i versus SB in IO-1 and IO-3, and we refer to these genes as R2i specific. Heatmaps V-VIII represent the down-regulated genes in R2i versus SB on days 1 and 3, and we consider these genes as SB specific.

(B) The expression patterns of 5 of 12 essential transcription factors for naïve pluripotency showed significant differences between IO-3 and SBIO-3. The expression of the other genes was not significantly different between R2i- and SB-treated IOs.

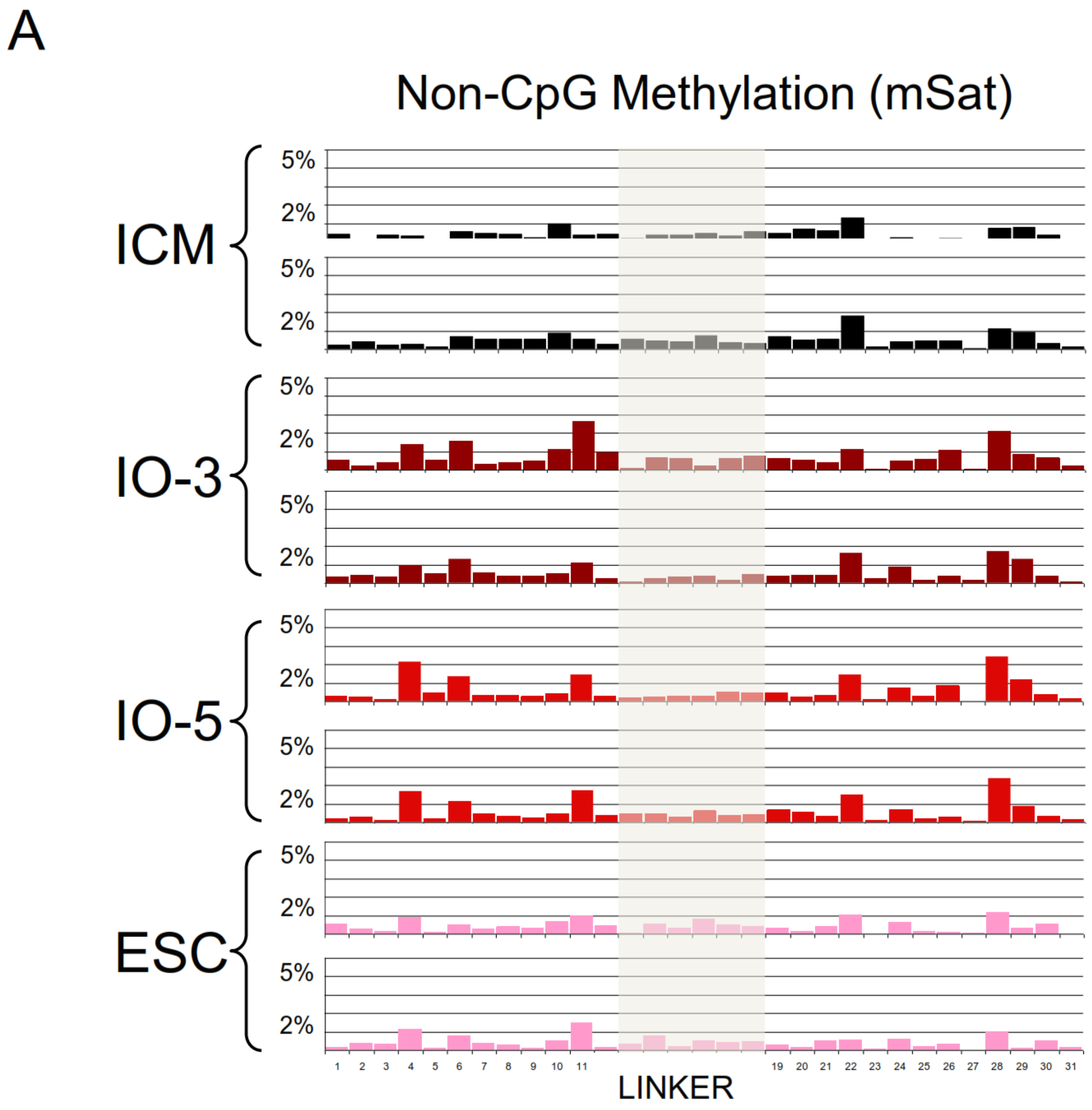

Figure S4. DNA methylation changes during the ICM to ESC transition, related to Figure 5.

Hairpin-bisulfite amplicon sequencing of non-CpG methylation of mSat. The bars sum up the DNA methylation status of all non-CpG positions.

A

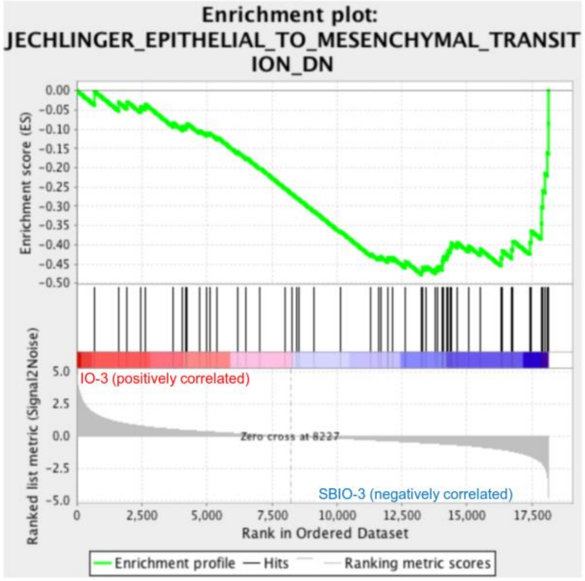

B

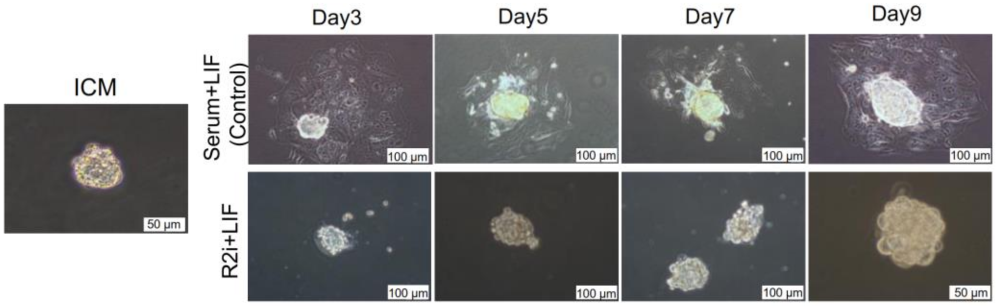

C

Passage 2, *Klf4*+SB+Lif

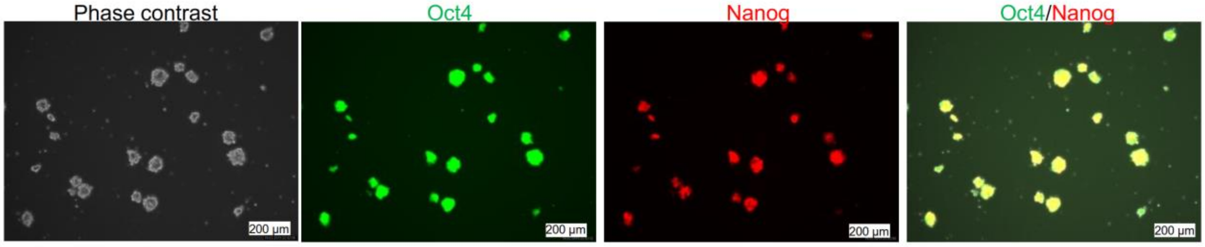

Figure S5. EMT blockage is required for the establishment of ESCs, related to Figure 6.

(A) GSEA indicated that SB-related genes were enriched in the EMT biological process.

(B) Morphological comparison between serum/LIF- and R2i-treated IOs. Note the expanded cell migration around the serum/LIF-treated IOs.

(C) Phenotype of established ESCs after *Klf4* induction in SB-treated IOs.

A

P15 vs E4.5 preEpi

Up-regulated

GO Term	P-val.
male meiosis I (GO:0007141)	5.05E-03
**KEGG pathway**	**P-val.**
Vasopressin-regulated water reabsorption_Homo sapiens_hsa04962	0.01
Proteoglycans in cancer_Homo sapiens_hsa05205	0.01
FoxO signaling pathway_Homo sapiens_hsa04068	0.01
p53 signaling pathway_Homo sapiens_hsa04115	9.97E-03
**Wikipathway**	**P-val.**
TOR Signaling_Homo sapiens_WP1471	4.94E-03
Alzheimers Disease_Mus musculus_WP2075	0.01
p53 signaling_Mus musculus_WP2902	6.83E-03

Dow-regulated

GO Term	P-val.
translation (GO:0006412)	2.69E-20
gene expression (GO:0010467)	2.78E-15
**KEGG pathway**	**P-val.**
Ribosome_Homo sapiens_hsa03010	4.6E-10
Oxidative phosphorylation_Homo sapiens_hsa00190	7.13E-07
**Wikipathway**	**P-val.**
Electron Transport Chain_Mus musculus_WP295	3.68E-07

B

	Heatmaps	E3.5 ICM	E3.5 ICM/E4.5 EPI	E4.5 EPI	E4.5 EPI/E5.5 EPI	E5.5 EPI	Diapaused
	Non-Sig	204	97	67	113	123	17
R2i	I	11	3	0	0	2	1
R2i	II	1	3	0	1	0	4
R2i	III	9	18	21	26	29	6
R2i	IV	2	0	1	3	1	1
SB	V	2	3	3	1	2	0
SB	VI	3	0	2	0	0	0
SB	VII	43	15	3	5	7	0
SB	VIII	3	0	1	0	3	0
	Total	278	139	98	149	167	29
	Boroviak (2015)	335	169	135	190	207	30

D

	Clusters	E3.5 EPI	E3.5-E4.5 EPI	E4.5 EPI	E3.5 PE	E3.5-E4.5 PE	E4.5 PE
	0	6	3	133	10	10	174
R2i	I	2	1	2	0	0	1
R2i	II	1	2	2	0	0	1
R2i	III	3	1	56	1	0	2
R2i	IV	0	0	2	0	0	0
SB	V	0	0	3	1	3	10
SB	VI	0	0	0	1	1	10
SB	VII	0	0	2	0	3	56
SB	VIII	0	0	2	0	0	3
	Total	12	7	202	13	17	257
	Gerovska (2016)	16	8	278	17	19	359

C

	ICM	ICM/Pre Epi 4.5	Pre Epi 4.5	Pre Epi/Post Epi	Post Epi	Diapaused
I	Eomes,Gsc,2610528J11Rik,Sri,Lgals4,Gm2a,Psap,AA467197,Serpinb6c	Zfhx2,Ostf1,Gpx1			Fxyd6,Cmtm8	Tcfap2c
II	BC053393	Jam2,Nanog,Klf2		Wfdc2		Klf2,Nanog,Dppa3,Msc
III	Upp1,Uhrf1,Serpina3m,Tuba4a,Ly6g6e,Mgmt,Fzd5,Arhgef19,Rfx2	Itgb7,Spp1,Ephx2,Tdgf1,Socs2,Ly6e,Aoah,Gdf3,Syt9,Sh3gl2,Oas1g,Vegfc,Zfp819,Tcl1,Ddx58,Stat4,Mybl2	Fez1,8430410A17Rik,Trh,Laptm5,Zfp428,Gsta4,Dnmt3l,Gpc4,Fstl1,Lrrc34,Tubb2b,Enox1,2610305D13Rik,Smtnl2,Pdcl,Hibadh,Dnd1,Esrrb,D14Ertd668e,Csrnp2,Cdc42ep3	Utf1,Sox2,Slc7a3,Zscan10,L1td1,Eml4,Nup210,Etv5,Sec14l1,Prdx1,Vim,Tmem40,Stmn2,Ifitm1,Pla2g1b,Bhlhb9,Ifitm3,Dek,Tpd52l1,Ak1,Ifitm2,Pipox,Zfp532,Psme4,Thy1,Tcea3	Igfbp2,Irf1,Hmga1,Cbr3,Pdk3,Rab34,Crabp2,Ina,Cd81,Pim2,Col18a1,Dab1,Fgf5,Phc1,Myb,Acot1,Sort1,Dtx1,Elmo1,Pou3f1,Klk8,Dnmt3b,Sall2,Enpp2,Cd276,Armcx2,Plekha1,2810417H13Rik,Otx2	Utf1,Sox2,Gdf3,Tdgf1,Esrrb,Tbx3
IV	Gna14,Axin2		Robo4	Zic3,Aire,Armcx1	Fzd7	Gbx2
V	Pnliprp2,Scmh1	Egr1,Dkk1,Col4a1	Zfp36l1,Htra1,Etv4	6330407J23Rik	Podxl,Emb	
VI	Aqp8,Pdgfra,Hnf4a				H19	
VII	Fhod1,Junb,Gpx2,Cpt2,Fgf10,Zyx,Zdhhc12,Rhou,Egfl7,Gss,Sgpl1,Acaa2,Polg,Serpinb6a,Ly6a,Nuak1,Ralb,Fbxo2,Amdhd2,Pvrl2,Ppap2c,Erp29,Elovl1,Cap1,Grina,Txndc12,Hexa,Gas2l1,Xist,Commd3,Adam15,Mfsd1,Gabarapl1,Tcf23,Myd88,Ap1m2,Stat3,Pdia5,Gdpd5,Plekhf1,Fbxo6,Tmbim1,Inadl	Serpinh1,Plod2,Mfge8,Rhox5,Rhoc,Col4a2,Apoc1,Retsat,Ext2,Pde1b,Srgn,Amn,Pga5,Ctgf,Cst3	Wdr1,S100a6,Ddah1	Obsl1,Bex2,Itm2b,Amfr,Txndc5	Car4,Cul7,Pxdn,Dusp9,Efs,2310021P13Rik	
VIII	Cldn3,Arpc1b,Wnt7b		Ndufs4		Tmem54,Tpm2,Lzts2	

E

	E3.5 EPI	E3.5 & E4.5 EPI	E4.5 EPI	E3.5 PE	E3.5 & E4.5 PE	E4.5 PE
I	Tcfap2c,Spic	Cldn4	Lgals3,AU018091			Serpinb6c
II	Slc1a1	Nanog,Klf2	Wfdc2,Pla2g10			Pdzk1
III	Sgk1,Ubxn2a,Liph	Zp3	Hmga1,Gsta4,Sox2,Slc7a3,Angptl4,Pim2,Smpdl3b,Rfx2,Sap30,Cdyl,Col18a1,Mkrn1,Socs2,Sirt1,Trh,Gtsf1l,Vegfc,Nptx2,Phc1,Stmn2,8430410A17Rik,Gprasp1,Zfp428,Tcea3,Mtss1,Tmem40,Otx2,Plekha4,Cth,Rcc2,Klhl7,Etv5,Dppa4,Capsl,Lrp11,Fntb,Nkrf,A830080D01Rik,Zfp532,Pcyt1b,Ddx58,Enox1,Esrrb,Ankrd6,Ulk1,Mbtd1,Gdf3,Bcat1,Tdgf1,Notum,Skil,Igfbp2,Mybl2,Trim37,Mical1,Btbd11	Dnmt3l		Sgk3,Pls3
IV			Spred1,Axin2			
V			Etv4,6330407J23Rik,Bcl2l11	Dusp4	Cpn1,Dkk1,Col4a1	Emb,Htra1,Tcfec,Rcn3,Gata6,Col4a1,Dnajc10,AA986860,Bmp6,P4ha1
VI				Tfpi	Pdgfra	Spink3,Aqp8,Ctsh,Pla2g12b,Ctdspl,Hnf4a,Pdgfra,Klb,Cobll1,Soat2
VII			Acaa2,Fbp2		P4ha2,Sox17,Serpinh1	Txndc12,Plod3,Cotl1,Tmed3,Cryab,Serping1,Plod2,Neu1,Tmem218,Zyx,Srgn,Atp6v0a1,Ctsz,Amn,Galnt10,Kdelr3,Pcbd1,Timd2,Rhox6,Mttp,Gaa,Nus1,Dab2,Pga5,Sox17,Polg,Hs3st1,Txndc5,Col4a2,Gdpd5,BC017158,Elovl1,Tmem144,Lrpap1,Galnt2,Agpat4,0610007C21Rik,2310021P13Rik,Gpr137b,Commd3,Dusp9,Myo6,Slc9a6,Ralb,Leprel1,Grina,Uap1l1,Xist,Nostrin,Lgmn,Cited1,Pdzd3,Gba,Myo5b,Coq2,Fut8
VIII			Ldoc1,Lasp1			Plac1,Krt8,Cldn7

Figure S6. R2i-treated IO indicated preEpi cell characteristics, related to Figure 7.

(A) Functional annotation for up- and down-regulated genes in ESCs of P15 versus E4.5 preEpi cells (Boroviak et al.).

(B) A comparison of gene expression shown in R2i- and SB-specific heatmaps (in Figure 3A) and those identified by Boroviak et al., which were associated with different lineages of the early mouse embryo.

(C) List of genes related to (B).

(D). A comparison of gene expression shown in R2i- and SB-specific heatmaps (in Figure 3A) and those genes identified by Gerovska and Arauzo-Bravo as associated with the epiblast (EPI) and primitive endoderm (PE).

(E) List of genes related to (D).

Supplemental Experimental Procedures

Mouse strains, E3.5 blastocyst collection

All mice were maintained on a 12-hour light/dark schedule. We used the following mice and embryo strains in this study: BALB/c (for the first run of the microarray and DNA methylation sequencing), NMRI (For RG108 treatment and R2i time-dependency experiment), F1 hybrids (B6 × C3H), (C57BL/6 × C3H) F1 female × CD1 male mice, and OG2 × F1 hybrids (B6 × C3H) (for the second run of the microarray and EMT/MET induction). All E3.5 blastocysts were collected by flushing the uterus after superovulation according to a previously described protocol (Hassani et al., 2012).

OG2 are transgenic mice in which GFP is expressed under the control of a Pou5F1 promoter and distal enhancer. This GFP reporter is expressed in the ICM of blastocysts on E3.5, the epiblast on E4.5, and in ESCs (Yeom et al., 1996). We used the Tet-On Nanog inducible blastocyst from F1 (C57BL/6 × C3H) × OG2 (Fischedick et al., 2014).

All procedures that used animals were in strict accordance with the approval of the Royan Institutional Review Board and Institutional Ethical Committee.

Culture medium

Serum-free N2B27 supplemented medium was the base medium for all experiments except where otherwise indicated. N2B27 supplemented medium consisted of DMEM/F12 (Invitrogen) and neurobasal (Invitrogen) in a 1:1 ratio, 1% N2 supplement (Invitrogen), 1% B27 supplement (Invitrogen), 1% nonessential amino acids (Invitrogen), 2 mM L-glutamine (Invitrogen), 100 U/ml penicillin and 100 mg/ml streptomycin (Invitrogen), 0.1 mM β-mercaptoethanol (Sigma-Aldrich), and 5 mg/mL BSA (Sigma-Aldrich). Mouse ESC (serum) medium consisted of

knockout Dulbecco's modified Eagle's medium (Invitrogen), 15% fetal bovine serum (FBS, HyClone), 1% nonessential amino acids, 2 mM L-glutamine, 100 U/ml penicillin, 100 mg/ml streptomycin, 0.1 mM β-mercaptoethanol, and 1000 U/ml mouse LIF.

Derivation of ESCs and sample collection

For isolation of the ICM, the zona of E3.5 blastocysts was removed using acidic Tyrode's solution (pH=2.2), and blastocysts were incubated with mouse trophoblast antibody for 40 min. Next, blastocysts were treated with guinea pig complement in 50 μl droplets under oil for 10 min. After bubbling and lysis of the trophectoderm cells, ICM cells were removed by pipetting. The isolated ICMs were washed twice in PBS, and then selected for microarray analysis. The procedure of deriving ESCs from early-stage mouse embryos was described previously in detail (Hassani et al., 2014b). Briefly, zona-free E3.5 blastocysts/isolated ICMs were plated on gelatin-coated plates (0.1%, Sigma-Aldrich) containing N2B27 defined medium supplemented with R2i (consisting of 1 μM PD0325901 [Stemgent] and 10 μM SB431542 [Sigma-Aldrich]) as well as 1000 U/ml LIF (ESGRO, Millipore). The day that E3.5 blastocysts/isolated ICMs were transferred into the ESC culture was designated as day 0. For the first microarray analysis, the samples included isolated ICMs (day 0), blastocyst outgrowths (BOs) on days 3, 5, 7, and 9 (BO3-9) after plating, and ESCs of passage 20, in three biological replicates. We chose and pooled approximately 20 to 30 isolated ICMs and BOs for each biological replicate.

For the second microarray analysis, we collected new samples in a time resolution experiment that included immunosurgically isolated ICMs, IOs on days 0.5, 1, 2, 3, and 5 (IO 0.5-5), and ESCs of passages 2, 4, and 15 (P2-15). In addition, we collected IOs cultivated in N2B27

supplemented with SB431542+LIF on days 1 (SBIO-1) and 3 (SBIO-3) as the negative controls. Approximately 30 to 40 ICMs or IOs were picked and pooled in two biological replicates.

RNA extraction, cDNA pre-amplification, and microarray profiling

Isolated ICMs, IOs, and ESCs were collected and preserved at -80°C until RNA extraction. Total RNA was isolated using the AllPrep DNA/RNA Micro Kit (QIAGEN). Integrity and quality of RNA samples was checked using a RNA 2100 Bioanalyzer (Agilent). RNA samples (RIN > 9) were subjected to a two-round amplification performed using the TargetAmp 2-Round Biotin-aRNA Amplification Kit 3.0 (Epicentre) according to the manufacturer's instructions. Purified and labeled cRNA was used for each hybridization reaction onto BeadChip Array Mouse WG-6 and MouseRef-8 v2.0 (Illumina, San Diego, CA, USA), and scanning was performed using the iScan reader (Illumina, San Diego, CA, USA).

Real-time PCR

Total RNA was isolated using the AllPrep DNA/RNA Micro Kit (QIAGEN). For quantitative RT-PCR, cDNA was generated using QuantiTect Whole Transcriptome Kit (QIAGEN) according to the manufacturer's protocol. All amplified cDNA samples were diluted 1 to 50, and 2 μl of each mixture was used for each qRT-PCR reaction. Gene expression experiments were performed using the SYBR green master mix and 7900HT Sequence Detection System (Life Science) in triplicate and two independent biological replicates. The amount of mRNA was normalized to the *Gapdh* and *Actb* housekeeping genes. Relative quantification of gene expression was calculated using the ΔΔCt method. All the primer sequences are listed as below:

Gene Symbol	Sequence	Size (bp)	Annealing Temperature (°C)	Accession number
Pou5f1(Oct4)	F: 5' gcg ttc tct ttg gaa agg tg 3'<br>R: 5' cgg ttc tca atg cta gtt cg 3'	204	61	NM_013633.2
Nanog	F: 5' ctg att ctt cta cca gtc cca 3'<br>R: 5' aaa cca ggt ctt aac ctg ctt at 3'	235	61	NM_028016.2
Esrrb	F: 5' agg ctc tca ttt ggg cct agc 3'<br>R: 5' atc ctt gcc tgc cac ctg tt 3'	102	61	NM_001159500.1
Tcf3	F: 5' cag cag tga cca gaa cag t 3'<br>R: 5' gaa gcc agc ctg act caa g 3'	195	61	NM_001164147.1
Klf4	F: 5' tgt gtc gga gga aga gga agc 3'<br>R: 5' acg act cac caa gca cca tca 3'	76	61	NM_010637
Cdh1	F: 5' cgg ata acc aga aca aag acc a 3'<br>R: 5' agc agg atc aga atc agc ag 3'	168	61	NM_009864.2
Dab2	F: 5' caa caa agc aga aga gaa tgg aag 3'<br>R:5' act att tag gtc agg agg tgt aga 3'	126	61	NM_023118.5
Eomes	F:5' gct tca aca taa acg gac tca 3'<br>R:5' cat ctt att gcc ctg cat gtt att 3'	145	61	NM_010136
Snail	F:5' tct gaa gat gca cat ccg aa 3'<br>R:5' act ggt atc tct tca cat ccg a 3'	208	61	NM_011427
Dnmt1	F:5' gga tga gag gga gga gaa gag 3'<br>R:5' cag gtt agg gtc gtc tag gt 3'	171	62	NM_001199433
Dnmt3l	F:5' cat cca gca ttac gtc ctc at 3'<br>R:5' tct cca ggt cca agg ttt caa 3'	159	61	NM_019448.4
Dnmt3b	F: 5' gtt tat atg agg gca cag gaa 3'<br>R: 5' gcc aca aca ttc tcg aac at 3'	114	62	NM_001003961
Suz12	F: 5' gcc ttt gag aaa cca aca cag 3'<br>R: 5' cag gac ttc cag ggt aac ag 3'	296	61	NM_199196
Sirt1	F: 5' gca cta att cca agt tct ata ccc 3'<br>R: 5' cac cac cta gcc tat gac ac 3'	141	61	NM_019812
Mat2b	F: 5' ggc aga gca gtt tac aaa gag 3'<br>R: 5' cac tat gac atg agg ctg ga 3'	152	61	NM_001199274
Ezh2	F: 5' tgc ttc cta cat ccc ttc ca 3'<br>R: 5' gtg gtg tct tta tac gct cag 3'	159	61	NM_007971

DNA methylation analysis by pyrosequencing

Genomic DNA from the ICM, ICM outgrowths on days 3 and 5, and ESCs was extracted using an AllPrep DNA/RNA Micro Kit (QIAGEN). The protocol for deep hairpin-bisulfite sequencing (DHBS) was previously described (Arand et al., 2012). Briefly, DNA was digested with an element-specific restriction enzyme followed by ligation of a hairpin oligonucleotide linker for major Satellites (mSat), the 5´ untranslated region of L1Md_Tf (L1), and a class of LTR-

retrotransposons (IAP-LTR1). Then, bisulfite-treated DNA was amplified by an element-specific PCR and sequenced with the standard 454-sequencer. BiQAnalyzerHT (Lutsik et al., 2011) was used to determine the methylation status of the CpG dyads and CNG positions.

Viral vectors

We examined the impact of EMT/MET induction on the derivation of ESCs by transducing isolated ICMs with a lentivirus that encoded mesenchymal or epithelial-related genes. To generate viral particles, we cloned *Snail-*, *Klf4-*, *Cdh1-2a-Tomato,* and *ShCdh1* into a pLVTHM (Han et al., 2011) vector backbone. As the control, we used a vector that encoded *Tomato*. Gene constructs were then transformed into DH5α bacteria, and plasmid DNA was extracted with a HiSpeed Plasmid Maxi Kit (QIAGEN). Next, plasmids were transfected into 293T packaging cells using the Fugene6 transfection reagent. After 6 hours, the medium was replaced by ESC medium. Supernatants were harvested for ICM infection after 24 hours. ICMs were cultured and transfected in the N2B27 base medium supplemented with 1 µM PD0325901 (Stemgent), 10 µM SB431542 (Sigma-Aldrich), and 1000 U/ml LIF (ESGRO®, Millipore) with 1 µg/ml polybrene (Sigma-Aldrich). The ICM cells were washed and replated after 24 hours. During this time, the medium was renewed every other day. Five days after ICM infection, ICM outgrowths were picked and disaggregated by dispase (1 mg/ml, Gibco). The dissociated cells were cultured in R2i. Once ESC colonies emerged, they were expanded for three passages to calculate the efficiency of deriving ESCs.

Immunofluorescence analysis

For immunofluorescence analysis, ICM outgrowths and ESCs were fixed in 4% paraformaldehyde (Sigma-Aldrich) for 20 minutes and then permeabilized with 0.2% Triton X-100 for 30 minutes. The procedure was followed by blocking the cells in 10% goat serum in PBS for 1 hour at room temperature and an incubating them overnight with primary antibodies at 4°C. Next, ICM outgrowths and ESCs were washed and incubated with NANOG secondary antibodies. Counterstaining of the nuclei was performed using 2 µg/ml DAPI (Sigma-Aldrich). A fluorescent microscope (Olympus, Japan) was used to visualize the cells.

Statistical and bioinformatics analysis

RAW expression values were extracted from Illumina GenomeStudio. Data was background corrected and quantile normalized by the "neqc" function of the limma R/Bioconductor package (Ritchie et al., 2015). Differentially expressed genes were identified by the Empirical Bayesian method of the limma package. We used the custom R program for visualization and data analysis. The pairwise correlation heatmaps for samples were generated based on Pearson Correlation Coefficients (PCC) using the R/Bioconductor package pheatmap. Principal Coordinates Analysis (PCA) was performed with the Multidimensional Scaling (MDS) method of package limma, and visualized using the package ggplot2. The numbers of differentially expressed genes between consecutive time points were calculated by 1 and 0.01 as the cutoffs for the absolute log2 fold change and Benjamini-Hochbert adjusted *p*-values, respectively. The same criteria were used for other differential expression analyses between our samples. The Gene Ontology (GO) and pathway analysis of differentially expressed genes were performed using Enrichr (Chen et al., 2013). Represented heatmaps of the genes in Figure 3A were created using the differential expression analysis of each gene for IO-1 versus SBIO-1 as well as for IO-3

versus SBIO-3. The clustering of the genes in Figure 4C was done using the K-means unsupervised clustering algorithm, as implemented in R. We used Gene Set Enrichment Analysis (GSEA) to analyze the enrichment of the curated gene sets from Molecular Signature Database (MSigDB) v5.2 among differentially expressed genes (Subramanian et al., 2005). The gene expression profiles of the early mouse embryo were obtained from a published study (Boroviak et al., 2015). We merged the expression values for the genes in that study with our samples based on their Entrez IDs. We used the ComBat algorithm of the R/Bioconductor package “sva” for Batch-effect removal (Leek et al., 2012). The values of 1 and 0.01 were the absolute log2-fold change and FDR thresholds for differential expression analyses, respectively, between the *in vivo* (early embryo samples) and *in vitro* (our samples).

Accession numbers

Raw and analyzed microarray data have been deposited in the NCBI Gene Expression Omnibus (GEO) as accession number GSE87793.

Supplemental Tables

Table S1. The list of genes and the expression value of represented heatmaps shown in Figure 3A, related to Figure 3.

Table S2. The list of common pluripotency, R2i pluripotency, and SB pluripotency–related genes based on published data, related to Figure 3.

Table S3. The number of reads and pattern of DNA methylation state between ICM, IOs, and ESC.

Table S4. Comparison of the expressed genes for the *in vivo* embryonic sample (Boroviak et al. 2015) and *in vitro*–cultured ICM-to-ESC samples of this study. Up: Up-regulated; Down: Down-regulated; NS: Non-significant, related to Figure 7.

Table S3. The number of reads and pattern of DNA methylation state between ICM, IOs, and ESC								
Sample	Amplicon	#reads	#CpGs	#mCpG/ mCpG	#mCpG/ CpG	#CpG/ mCpG	#CpG/ CpG	Conversion rate (linker)
ICM	IAPLTR	284	1303	820	129	191	163	0.993
IO-3	IAPLTR	564	2611	1416	373	397	425	0.996
IO-5	IAPLTR	492	2297	1280	344	288	385	0.994
ESC	IAPLTR	510	2329	1815	172	167	175	0.994
ICM	mSat	2514	6809	2443	448	568	3350	0.982
ICM	mSat	2465	6683	2340	369	422	3552	0.976
IO-3	mSat	1624	4393	1761	360	474	1798	0.967
IO-3	mSat	1967	5387	2315	551	493	2028	0.965
IO-5	mSat	2069	5623	2582	437	436	2168	0.967
IO-5	mSat	2338	6307	2932	416	517	2442	0.976
ESC	mSat	2151	5792	2838	378	537	2039	0.969
ESC	mSat	1907	5152	2476	329	464	1883	0.959
ICM	L1	1692	7960	1825	517	460	5158	0.986
ICM	L1	2431	11655	2446	776	546	7887	0.986
IO-3	L1	2177	10444	2354	1077	987	6026	0.984
IO-3	L1	793	3640	907	411	363	1959	0.97
IO-5	L1	2565	12285	3140	1187	1027	6931	0.985
IO-5	L1	1022	4903	1192	509	389	2813	0.988
ESC	L1	1777	8483	2195	942	724	4622	0.985
ESC	L1	2383	11411	2912	1223	1034	6242	0.984

Supplemental References

Arand, J., Spieler, D., Karius, T., Branco, M.R., Meilinger, D., Meissner, A., Jenuwein, T., Xu, G., Leonhardt, H., Wolf, V., Walter, J., 2012. In vivo control of CpG and non-CpG DNA methylation by DNA methyltransferases. PLoS Genet. 8. doi:10.1371/journal.pgen.1002750

Boroviak, T., Loos, R., Lombard, P., Okahara, J., Behr, R., Sasaki, E., Nichols, J., Smith, A., Bertone, P., 2015. Lineage-Specific Profiling Delineates the Emergence and Progression of Naive Pluripotency in Mammalian Embryogenesis. Dev. Cell 35, 366–382. doi:10.1016/j.devcel.2015.10.011

Chen, E.Y., Tan, C.M., Kou, Y., Duan, Q., Wang, Z., Meirelles, G.V., Clark, N.R., Ma'ayan, A., 2013. Enrichr: interactive and collaborative HTML5 gene list enrichment analysis tool. BMC Bioinformatics 14, 128. doi:10.1186/1471-2105-14-128

Fischedick, G., Wu, G., Adachi, K., Araúzo-Bravo, M.J., Greber, B., Radstaak, M., Köhler, G., Tapia, N., Iacone, R., Anastassiadis, K., Schöler, H.R., Zaehres, H., 2014. Nanog induces hyperplasia without initiating tumors. Stem Cell Res. 13, 300–315. doi:10.1016/j.scr.2014.08.001

Han, D.W., Greber, B., Wu, G., Tapia, N., Araúzo-Bravo, M.J., Ko, K., Bernemann, C., Stehling, M., Schöler, H.R., 2011. Direct reprogramming of fibroblasts into epiblast stem cells. Nat. Cell Biol. 13, 66–71. doi:10.1038/ncb2136

Leek, J.T., Johnson, W.E., Parker, H.S., Jaffe, A.E., Storey, J.D., 2012. The SVA package for removing batch effects and other unwanted variation in high-throughput experiments. Bioinformatics 28, 882–883. doi:10.1093/bioinformatics/bts034

Lutsik, P., Feuerbach, L., Arand, J., Lengauer, T., Walter, J., Bock, C., 2011. BiQ Analyzer HT: Locus-specific analysis of DNA methylation by high-throughput bisulfite sequencing. Nucleic Acids Res. 39, 551–556. doi:10.1093/nar/gkr312

Ritchie, M.E., Phipson, B., Wu, D., Hu, Y., Law, C.W., Shi, W., Smyth, G.K., 2015. limma powers differential expression analyses for RNA-sequencing and microarray studies. Nucleic Acids Res. 43, e47. doi:10.1093/nar/gkv007

Subramanian, A., Tamayo, P., Mootha, V.K., Mukherjee, S., Ebert, B.L., Gillette, M. a, Paulovich, A., Pomeroy, S.L., Golub, T.R., Lander, E.S., Mesirov, J.P., 2005. Gene set enrichment analysis: a knowledge-based approach for interpreting genome-wide expression profiles. Proc. Natl. Acad. Sci. U. S. A. 102, 15545–50. doi:10.1073/pnas.0506580102

Yeom, Y.I., Fuhrmann, G., Ovitt, C.E., Brehm, a, Ohbo, K., Gross, M., Hübner, K., Schöler, H.R., Hubner, K., Scholer, H.R., 1996. Germline regulatory element of Oct-4 specific for the totipotent cycle of embryonal cells. Development 122, 881–894.
